# Fused Nickel(II) Porphyrins—Sensing of Toxic Anions and Selected Metal Ions Through Supramolecular Interactions

**DOI:** 10.3389/fchem.2020.595177

**Published:** 2020-11-17

**Authors:** Tawseef Ahmad Dar, Muniappan Sankar

**Affiliations:** Department of Chemistry, Indian Institute of Technology, Roorkee, India

**Keywords:** fused porphyrin, sensor, toxic ions, colorimetric, reusability

## Abstract

Ni(II) porphyrins having fused –NH group were synthesized and characterized by various spectroscopic techniques. The fused porphyrins **1** and **2** were used to detect species of opposite polarity. **1** was used to sense toxic anions *viz*. cyanide and fluoride ions whereas **2** was used for detecting some selective metal ions including toxic mercury(II) ions. **1** is having acidic –NH proton, which detects anions *via* hydrogen bonding interactions followed by anion-induced deprotonation. On the other hand, **2** senses the metal species *via* weak charge transfer interactions from oxygen atom of the formyl group to the added metal ions. The limit of detection was calculated in case of **1** as 2.13 and 3.15 ppm for cyanide and fluoride ions, respectively. Similarly, the detection limit was found to be 0.930, 2.231, and 0.718 ppm for Cu(II), Fe(III), and Hg(II) ions, respectively, for probe **2**. The probes were recovered and reused for several cycles.

**Graphical Abstract:**
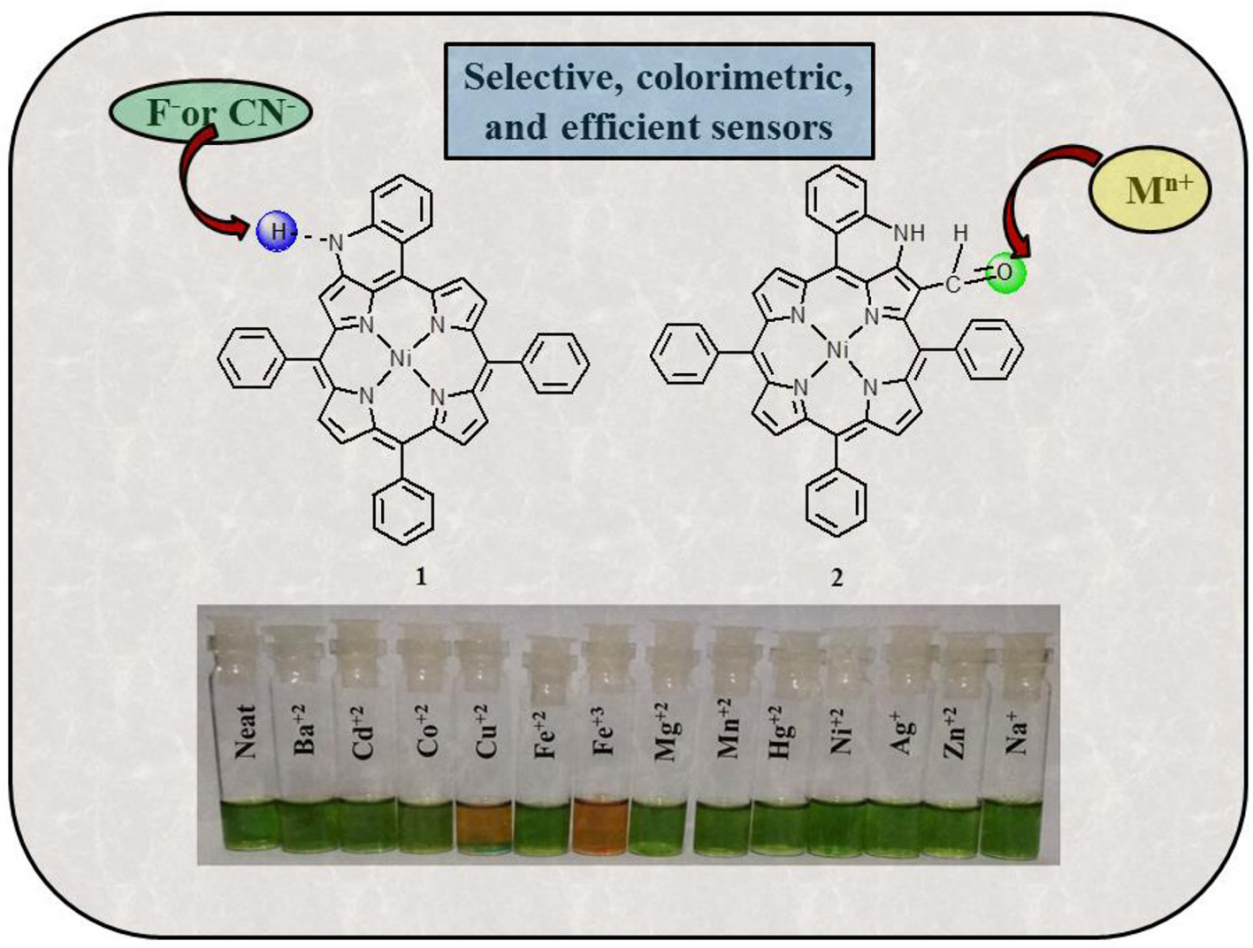
Detecting toxic anions and cations using NH-fused Ni(II) chemosensors.

## Highlights

- Ni(II) porphyrins having fused –NH group (**1**) and along with CHO functionality (**2**) have been synthesized and characterized.
- **1** and **2** act as colorimetric sensors for the detection of toxic ions (cations as well as anions) with low detection limits.
- **1** was utilized for detecting toxic CN^−^ and F^−^ ions *via* anion induced deprotonation.
- **2** was utilized to detect Cu(II), Fe(III), and Hg(II) metal ions through chelation of CHO and NH.
- These sensors are reversible and reusable.

## Introduction

A large number of species have been pumped into our environment because of rapid industrialization that are harmful to living beings including humans (Duan et al., 2015). The detection of such harmful and toxic species is very urgent and has thus emerged as a hot area of research over the past decades. In addition to detecting toxic species, some other species need to be detected such as peptides and proteins (Kubota and Hamachi, 2015), molecular oxygen (Ramamoorthy et al., 2003), CO_2_ (Liu et al., 2010), explosives (Wang et al., 2016), moisture (Han et al., 2017), and so on. Highly sophisticated tools to detect various analytes such as Fourier transform infrared, gas chromatography–mass spectrometry, electrochemical analysis, and absorption spectroscopy have existed in the domain for quite some time (Paolesse et al., 2017). However, these techniques are expensive and also require a high degree of expertise, which makes their widespread use a limited affair. This drawback cleared the way for the development of molecule-based chemical probes. A chemical probe is a molecule/species that on binding to a specific analyte gives rise to a distinct signal, which is noticeable in terms of change in color, change in absorption/emission, change in conformation, and so on (Balaji et al., 2006).

Anions play many significant roles in living systems as well as in industries. For example, fluoride prevents tooth decay, and hypochlorite is used as disinfectant for drinking water (Kirk, 1991). DNA as a polyanion carries genetic information (Beer and Gale, 2001). Many enzymatic cofactors are anionic such as carboxypeptidase A (Christianson and Lipscomb, 1989). Cyanide is used in metallurgical operations for extracting precious metals such as gold and silver (Akcil et al., 2015). However, the mere presence of cyanide and excess amount of fluoride in living tissues becomes a health hazard that can become life threatening if left untreated (Bassin et al., 2006; Bhattacharya and Flora, 2015). The toxicity associated with fluoride and cyanide demands the development of sensors to monitor the food items that we consume in our day-to-day lives. A large number of cyanide and fluoride ion sensors that work through different mechanisms are already known in literature (Xu et al., 2010; Zhou et al., 2014; Marchetti et al., 2018; Montis et al., 2019). Anthraimidazolediones detect cyanide and fluoride ions *via* intramolecular charge transfer, bringing out an easily detectable color change in organic media (Kumari et al., 2011). Contrary to this, anthraimidazolyldione-based sensor detects cyanide and fluoride ions *via* anion-induced deprotonation and breakage of C-Si bond, which begets a red fluorescent response (Mahapatra et al., 2015). Ammonium boranes detect cyanide and fluoride ions *via* complexation interactions on the basis of electronic and steric factors in aqueous media (Hudnall and Gabbaï, 2007). Ferrocene-derivatized moieties detect cyanide and fluoride ions *via* Lewis acid–base interactions inducing colorimetric response in acetonitrile/methanol medium (Broomsgrove et al., 2008).

Metal cations are equally important to living systems as their anionic counterparts (Anastassopoulou and Theophanides, 1995). Mg^+2^ is central to the most important anabolic reaction of photosynthesis in plants (Farhat et al., 2016). Mg^+2^ is also a part of many enzymes and helps in releasing the energy trapped in ATP by stabilizing the transition state during ATP hydrolysis (Williams, 2000; Maguire and Cowan, 2002). Cu^+2^ helps in electron transport and oxygen transport and is a part of enzymes such as dismutases and reductases (Marklund, 1982; Karlin and Tyeklár, 1993). The Na^+^/K^+^ pump is used by animal cells to maintain an ion gradient for performing many specialized functions (Clausen et al., 2017). Ca^+2^ is essential for healthy bones, teeth, cell signaling, nerve transmission, and muscle function (Michael and Whitaker, 2010). Like anions, however, some metal ions are toxic at high concentrations, whereas some others are toxic at all concentrations (Quang and Kim, 2010). Some of the well-known defects associated with metal imbalance are Parkinson disease, Wilson disease, and Alzheimer disease (Bush, 2000; Dusek et al., 2015). The adversities associated with metal cations warrant the need for chemical sensors that can easily and selectively detect the cations at low concentrations. A number of mechanistically different sensors are already available to detect different metal ions (Jeong and Yoon, 2012; Kumar et al., 2017). A fluorescein-based chemosensor has been reported to detect Cu^+2^ ions in aqueous solutions in nanomolar concentrations (Jun et al., 2006). Boronic acid–rhodamine–linked fluorescent, colorimetric sensors have been reported for Cu^+2^ ions on the basis of spirolactam ring opening in acetonitrile medium (Swamy et al., 2008). An oligodeoxyribonucleotide-based sensor is known to detect Hg^+2^ ions in aqueous medium (Ono and Togashi, 2004). A host of other techniques are available to detect mercuric ions in various media (Nolan and Lippard, 2008). Similarly, various mechanisms to detect Fe^+3^ ions are documented in literature (Wu et al., 2010; El-Safty and Shenashen, 2013).

Porphyrins because of their aromaticity, easy functionalization, and high stability have been used for multiple applications (Nam et al., 2003; Senge et al., 2007; Li and Diau, 2013). In the field of sensing and sensor materials, porphyrins have been found to be very useful and go to species (Ding et al., 2017). Porphyrin-based chemosensors have been reported for cyanide (Xu et al., 2010), fluoride (Kubo et al., 2003), nitrite (Yang et al., 2014), azide (Zhang et al., 2012), chloride (Zhang et al., 2005), and phosphate anions (Rodrigues et al., 2014). Similarly, metal cations that have been detected using porphyrin chemosensors include zinc(II) (Zhang et al., 2007), nickel(II) (Malinski et al., 1990), mercury(II) (Zhang et al., 2002; Bai et al., 2020), iron(III) (Vlascici et al., 2012), copper(II) (Gupta et al., 2006), lead(II) (Bozkurt et al., 2009), and so on. Our research group has already reported sensors based on porphyrin analogs for cyanide ions, fluoride ions, and picric acid (Chahal and Sankar, 2017; Dar and Sankar, 2017; Rathi et al., 2017). In our quest to develop sensitive porphyrin chemosensors, we hereby report the use of –NH fused porphyrins for detecting toxic cyanide and fluoride anions and also copper(II), iron(III), and mercury(II) metal cations.

## Experimental Section

### Materials and Methods

Propionic acid, acetic acid, P_2_O_5_, acetic anhydride, sodium sulfate, and sodium acetate were procured from Thomas Baker, India, and used as such. Benzaldehyde, Ni(OAc)_2_. 4H_2_O, NaHCO_3_, and various tetrabutylammonium salts were obtained from HiMedia, India, and used as received. DMF and CH_3_CN were purchased from Merck, India, and used further processing. Pyrrole was purchased from SRL Chemicals, India, and used as received. 1,2-Dichlorobenzene, triethylphosphite, POCl_3_, and metal perchlorate salts were purchased from Alfa Aesar, UK, and used as such. Silica gel (100–200 mesh) was purchased from Rankem, India, and used as received. Solvents used in the current study such as hexane, chloroform, dichloromethane, and methanol were procured from Molychem, India, and used after drying over P_2_O_5_. Copper(II) nitrate was purchased from Avra Synthesis, India, and used without purification. Deuterated solvents for nuclear magnetic resonance (NMR) studies were purchased from Sigma–Aldrich, India.

### Instrumentation

Optical absorption spectral studies were carried out in dry CH_2_Cl_2_ (or CHCl_3_) using an Agilent Cary 100 spectrophotometer using a pair of quartz cells of 1-cm path length and 3.5 mL capacity. ^1^H-NMR spectra were recorded using JEOL ECX 400-MHz spectrometer using DMSO-*d*_6_ and CDCl_3_ as solvents.

### Synthetic Procedures

Nickel(II) porphyrins **1** and **2** were synthesized by reported literature methods (Richeter et al., 2004) and analyzed by different spectral techniques such as absorption spectroscopy and ^1^H-NMR.

#### Nickel β-N-Fused-*meso*-Tetraphenylporphyrin (1)

^1^H-NMR (400 MHz,CDCl_3_) δ (ppm): 9.44, 8.83 (AB quartet, 2H, *J* = 4 Hz, pyrrole), 8.62, 8.57 (2s, 2 + 2H, pyrrole), 8.01 (s, 1H, pyrrole), 8.91 (dd, 1H, *J* = 8 Hz and 1 Hz, cyclized phenyl), 7.98–7.97 (m, 6H, H_ortho_), 7.72–7.64 (m, 12H, 9H_meta+para_ + 3H cyclized phenyl), 9.22 (broad s, 1H, NH). UV/vis (CH_2_Cl_2_): λ_max_ (nm) (log ε): 424(4.85), 552 (3.78), 596 (3.93), 628 (4.18). Anal. Calcd for C_44_H_27_N_5_Ni: C, 77.22; H, 3.98; N, 10.23. Found: C, 76.97; H, 4.08; N, 9.97.

#### Nickel β-N-Fused-β′-Formyl-*meso*-Tetraphenylporphyrin (2)

^1^H-NMR (400 MHz,CDCl_3_) δ (ppm): 9.35, 8.79 (d, 1 + 1H, *J* = 4 Hz, pyrrole), 8.60, 8.59 (AB quartet, 2H, *J* = 4 Hz, pyrrole), 8.52, 8.51 (AB quartet, 2H, *J* = 4 Hz, pyrrole), 8.96 (dd, 1H, *J* = 8 Hz and 1 Hz, cyclized phenyl), 8.04 (d, 1H, *J* = 8 Hz, cyclized phenyl), 7.96–7.94 (m, 6H, H_ortho_), 7.80–7.64 (m, 11H, 9H_meta+para_+ 2H cyclized phenyl), 12.50 (broad s, 1H, NH), 9.07 (s, 1H, CHO). UV/vis (CH_2_Cl_2_): λ_max_ (nm) (log ε) 447(5.08), 557(4.00), 628(4.26). Anal. Calcd for C_45_H_27_N_5_NiO•0.5CHCl_3_: C, 75.87; H, 3.82; N, 9.83. Found: C, 76.01; H, 4.03; N, 9.99.

## Results and Discussion

### Synthesis and Characterization

**Figure 1:**
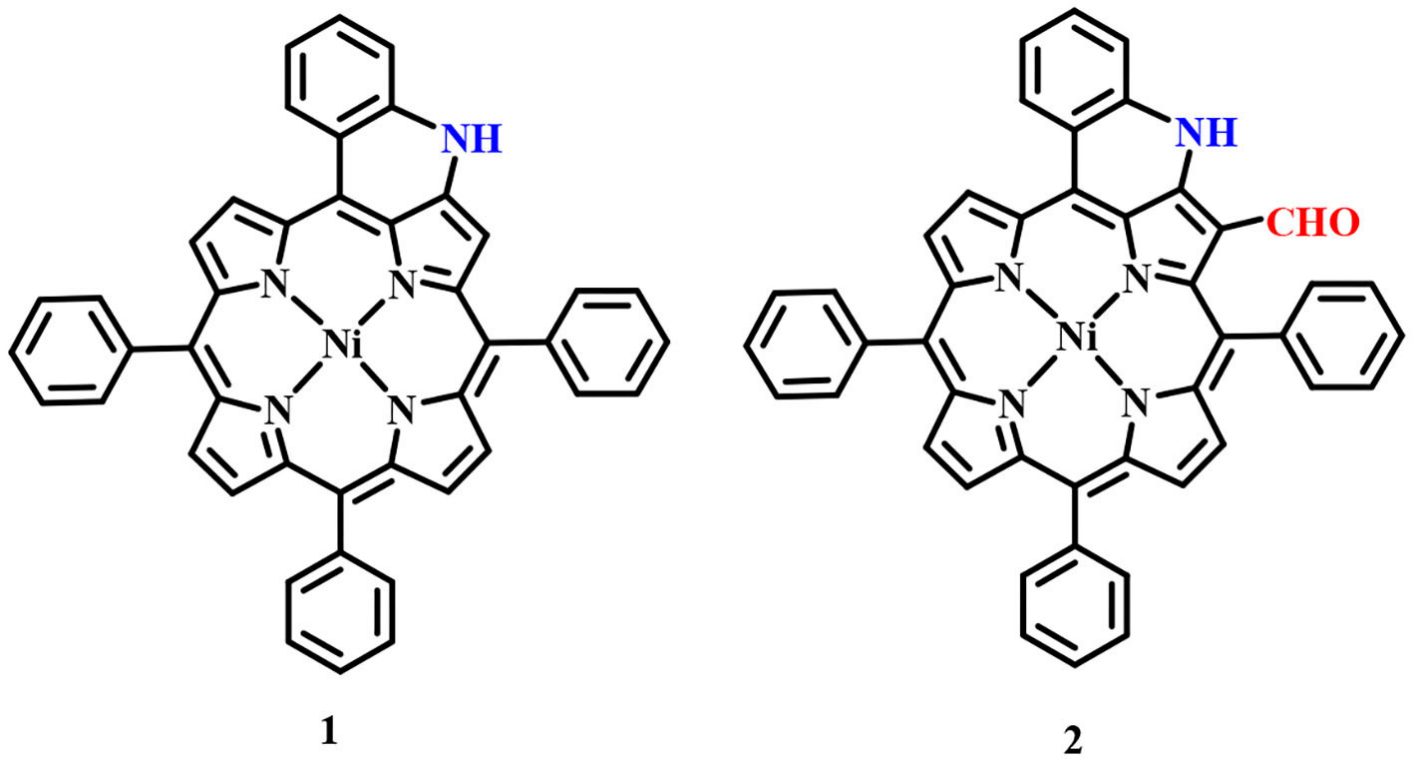
Chemical structures of sensors **1** and **2** used for detecting anions and cations, respectively.

Fused porphyrins **1** and **2** were synthesized by reported literature methods, with H_2_TPP being the precursor molecule (Richeter et al., 2004). Supplementary Figures 1–3 represent the UV-visible spectra and ^1^H-NMR spectra of sensors **1** and **2**. Supplementary Table 1 lists the UV-Vis spectral data of **1** and **2** in the SI. **1** bears fused –NH moieties, and its acidic nature was explored for selectively detecting cyanide and fluoride ions (Xu et al., 2010). **2** has a free –CHO group in addition to a fused –NH group and was found to interact selectively with copper(II), iron(III), and mercury(II) ions among an assortment of metal cations. The molecular structures of sensors **1** and **2** are shown in Figure 1. The interaction of **1** and **2** with their respective analytes induced color changes, making it possible to detect the analytes with naked eyes. The binding interactions were additionally monitored by changes in UV-visible and ^1^H-NMR spectra.

## Anion Sensing by Nickel β-N-Fused-*meso*-Tetraphenylporphyrin (1)

### UV-Visible Spectral Titrations

UV-visible spectral titrations of various anions were performed against a standard solution of **1** in distilled CH_2_Cl_2_ at 298 K. Stock solution of **1** (2.23 × 10^−5^ M) was prepared in distilled CH_2_Cl_2_. Similarly, stock solutions of 10^−2^ M concentration of various anions such as ${\text{ClO}}_{4}^{-}$, Br^−^, Cl^−^, CN^−^, F^−^, H_2_${\text{PO}}_{4}^{-}$, ${\text{HSO}}_{4}^{-}$, I^−^, CH_3_COO^−^, and ${\text{PF}}_{6}^{-}$ were also prepared in distilled CH_2_Cl_2_ (from their tetrabutylammonium salts). UV-visible titrations were performed by adding 3-μL aliquots from the various anion solutions to a 3-mL solution of sensor **1** independently. Majority of the anions viz. Br^−^, Cl^−^, ${\text{ClO}}_{4}^{-}$, H_2_${\text{PO}}_{4}^{-}$, ${\text{HSO}}_{4}^{-}$, I^−^, CH_3_COO^−^, and ${\text{PF}}_{6}^{-}$ failed to bring any noticeable changes in the UV-visible spectrum of **1**. Adding excess amount (50 eq.) of these anions again had no effect on the UV-visible spectrum of **1** (Supplementary Figures 4–11).

**Figure 2:**
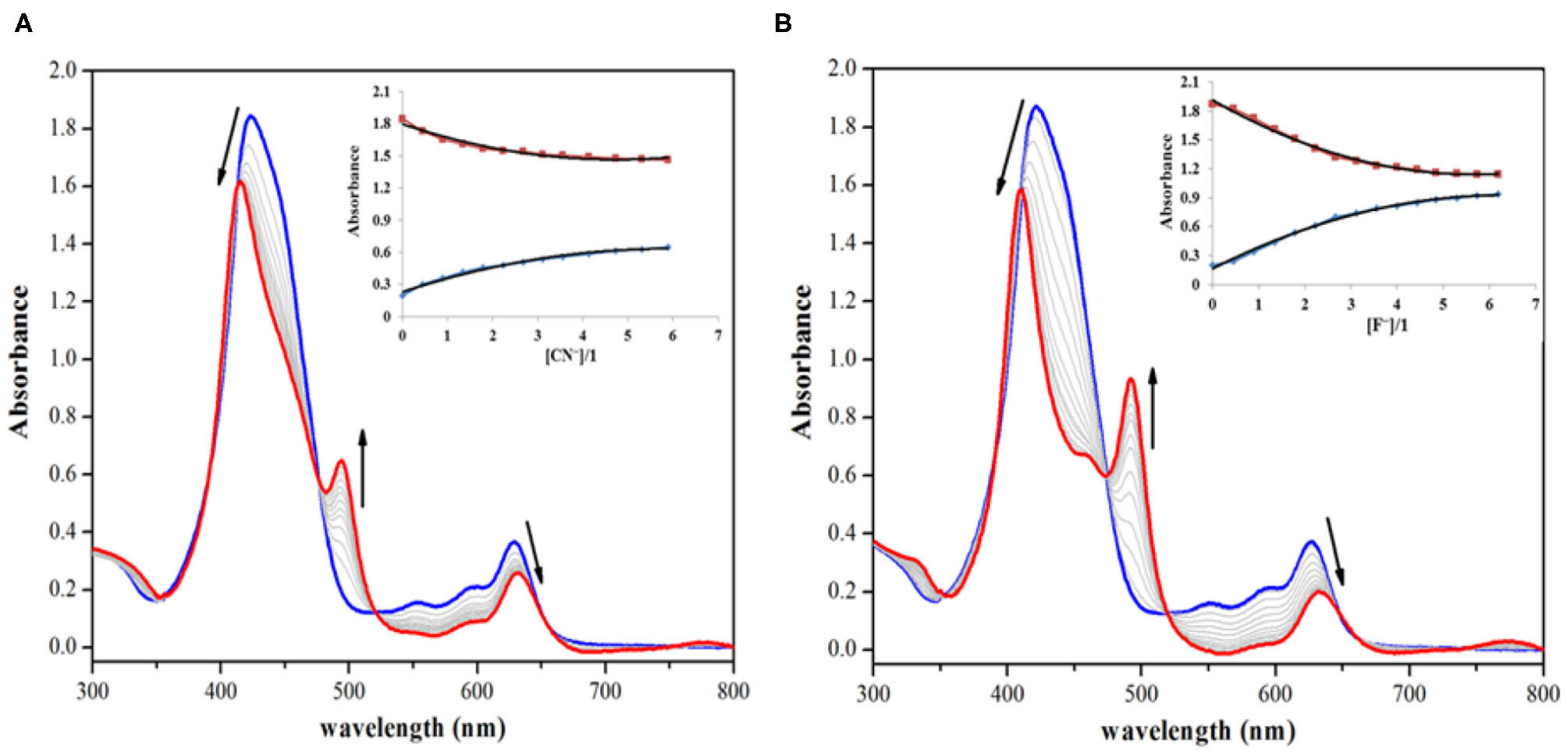
Absorption spectral titrations of **1** on addition of **(A)** 0–1.56 × 10^−4^ M, 7 equiv. of CN^−^ ions and **(B)** 0–1.56 × 10^−4^ M, 7 equiv. of F^−^ ions in distilled CH_2_Cl_2_ at 298 K. Insets show decreasing absorbance at 424 nm and the collateral increasing absorbance at 493 nm against [anion conc./**1** conc.].

On the other hand, addition of CN^−^ and F^−^ ions brought significant changes to the absorption spectrum of **1**. The CN^−^ and F^−^ ions interact specifically with the fused (acidic) –NH proton, which leads to deprotonation causing clear changes in the absorption spectrum of **1** and also inducing color changes from green to reddish yellow. On addition of roughly 7 equiv. of these anions to the clear solution of **1**, the intensity of Soret band at 424 nm decreases, along with a small hypsochromic shift. When CN^−^ ion was added from 0 to 1.56 × 10^−4^ M (7.0 equiv.), the intensity of the Soret band at 424 nm started diminishing along with a small blue shift (Δλ_max_ = 14 nm). Each successive addition further affected the intensity and position of the Soret band until its stabilization at 410 nm along with the appearance of a new band at 493 nm as shown in Figure 2A. The Q-bands at 552 and 596 nm almost disappeared due to CN^−^ addition, whereas the last Q-band at 628 nm was red shifted to 633 nm (Δλ_max_ = 5 nm) (Figure 2A). With further addition of CN^−^ ions, no more variations in the absorption spectrum were observed. Very similar changes were observed in the absorption spectrum of **1** on adding F^−^ ions. On adding 0–1.56 × 10^−4^ M (7.0 equiv.) of F^−^ ions, the Soret band at 424 nm shifted hypsochromically (Δλ_max_ = 14 nm), and a new band rose at 493 nm, which is similar to the pattern observed in case of CN^−^ ions (Figure 2B). The Q-band at 628 nm shifted bathochromically to 633 nm (Δλ_max_ = 5 nm). The shifting of Soret band to 410 nm and the appearance of a new band at 493 nm are due to deprotonation of the fused –NH protons, which is brought about by CN^−^ and F^−^ ions acting as strong bases. Such changes in the absorption spectra of porphyrins due to deprotonation have already been reported in literature (Guo et al., 2006; Shundo et al., 2009).

Deprotonation was responsible for the observed changes in the UV-visible spectrum of **1** (on adding CN^−^ and F^−^ ions) because UV-visible titration of **1** against TBAOH (deprotonating agent) affected much similar changes. When titration was carried out by adding 0–7.80 × 10^−5^ M (3.5 equiv.) of OH^−^ ions to the stock solution of **1**, the final spectral pattern resembled the spectral patterns obtained by addition of CN^−^ and F^−^ ions (Supplementary Figure 12). The absorption spectral changes were observed only in case of CN^−^ and F^−^ ions and not in case of Br^−^, Cl^−^, ${\text{ClO}}_{4}^{-}$, H_2_${\text{PO}}_{4}^{-}$, ${\text{HSO}}_{4}^{-}$, I^−^, CH_3_COO^−^, and ${\text{PF}}_{6}^{-}$; hence, **1** can be selectively used to detect toxic CN^−^ and F^−^ ions, which are highly basic and smaller in size in comparison to other anions. As sensor **1** is non-fluorescent, hence fluorescence titration of **1** with different anions was not possible. The addition of various anions did not enhance the fluorescence of sensor **1**, which continued to remain non-fluorescent.

### ^1^H-NMR Titration

**Figure 3:**
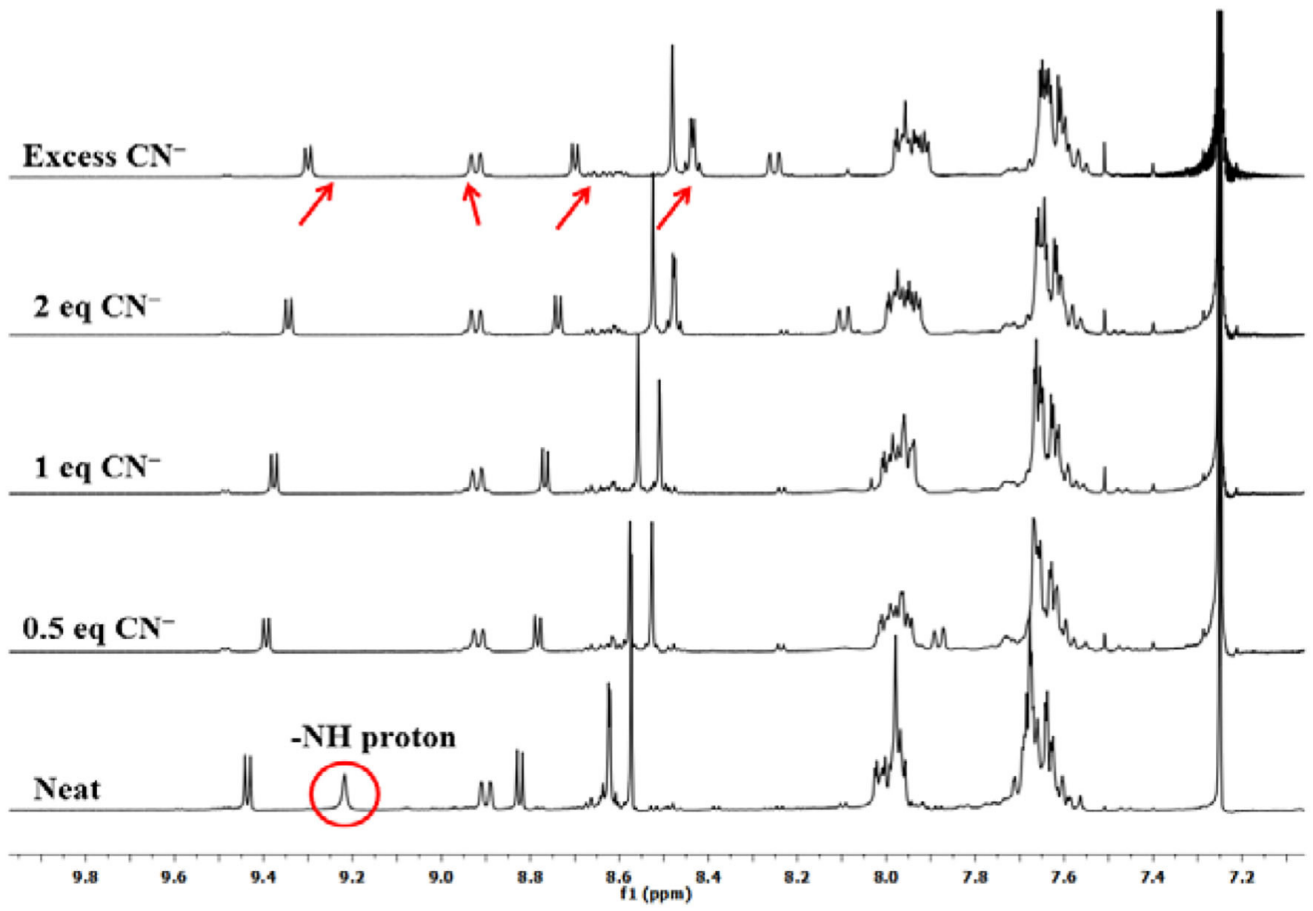
^1^H-NMR titration of **1** with CN^−^ ions in CDCl_3_ at 298 K.

To explain the observed spectral changes, ^1^H-NMR titration of **1** with CN^−^ and F^−^ ions in CDCl_3_ was performed at 298 K. ^1^H-NMR titration of **1** was also carried out with OH^−^ ions under similar conditions (as a control measure). When pure, sensor **1** shows fused –NH protons at 9.22 ppm, and addition of as little as 0.5 equiv. of CN^−^ ions led to vanishing of this peak with minute changes in the position of other peaks. When the amount of added CN^−^ ions was increased to 1 equiv., no more changes in the spectrum were observed. Addition of excess amount of CN^−^ ions (5 equiv.) had no significant effect as a very marginal shift in different NMR peaks was observed, for example, 0.13 ppm (upfield) in pyrrolic β-proton near –NH, 0.02 ppm (downfield) in case of cyclized phenyl proton, 0.12 ppm (upfield) in pyrrolic proton (other side of cyclized phenyl), 0.18 ppm (upfield), and 0.13 ppm (upfield) in case of two sets of pyrrolic protons (two each), 0.02 ppm (upfield) in case of ortho phenyl protons, and 0.07 ppm (upfield) in *meta-* and *para*-phenyl protons as shown in Figure 3. Disappearance of the peak corresponding to fused –NH protons is the only sharp and critical change in the ^1^H-NMR spectrum; hence, it can be concluded that deprotonation is the reason behind the observed absorption spectral changes on anion addition to **1**. It has to be noted that no other reactive atom/group is present in probe **1**, which can potentially interact with the added anions.

Titration with F^−^ and OH^−^ ions affected similar changes in the NMR spectrum of **1** (Supplementary Figures 13, 14). In case of ^1^H-NMR titration of **1** with OH^−^ ions, the fused –NH protons do not disappear instantly, and the –NH signal broadens due to rapid exchange of protons with methanol (commercial TBAOH comes as 0.1 M solution in methanol). However, on adding 2 equiv. OH^−^ ions, the –NH peak disappeared, and marginal shifts were observed for other peaks. These ^1^H-NMR titrations of sensor **1** clearly lead to the conclusion that deprotonation of fused–NH protons is mainly responsible for the exhibited spectral changes. Other anions do not deprotonate fused –NH protons leading to selective distinguishing of highly basic and poisonous CN^−^ and F^−^ ions.

### Colorimetric Studies and Naked-Eye Detection

**Figure 4:**
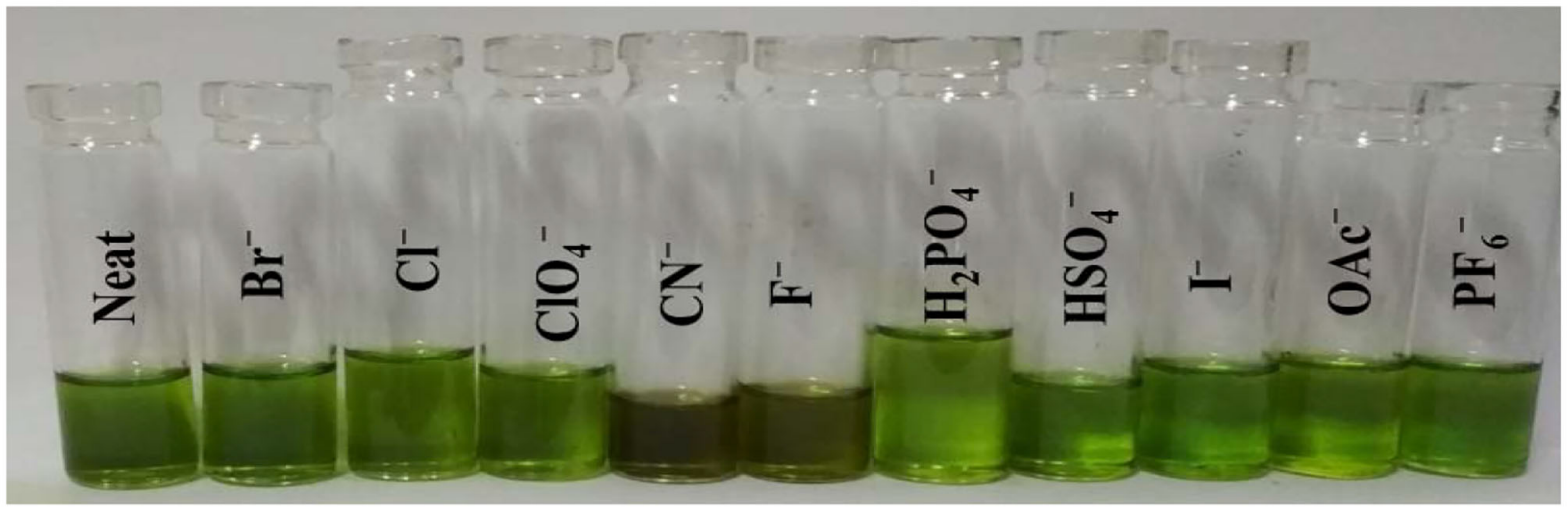
Colorimetric response of **1** using 4 equiv. of various anions in CH_2_Cl_2_ at 298 K.

Colorimetric sensors have gained significant ground in the recent past as they help the observer detect species of importance through naked eyes and without any expertise on behalf of the user. Colorimetric studies were carried out to study how anion addition affects the color of porphyrin sensor **1**. To carry out the colorimetric studies, 15 μM solution of **1** was prepared in distilled CH_2_Cl_2_. Identical sample vials were filled with roughly 1 mL of the prepared solution. One of the solutions was kept pure and to the other sample vials, 4 equivalents of different anions (also prepared in CH_2_Cl_2_) were added. A sharp color change from clear green to reddish yellow was observed in the solutions to which CN^−^ and F^−^ ions were added as shown in Figure 4. The observed color changes support the anion-induced deprotonation of **1** brought about by poisonous CN^−^ and F^−^ ions because of their highly basic character. This anion-induced color change makes it possible to selectively detect CN^−^ and F^−^ ions through naked eyes (Figure 4).

### Reversibility Studies

Fused –NH probe **1** is reversible in nature and can be reused for many cycles when regenerated by washing with distilled water. As detailed above, **1** undergoes a sharp color change from clear green to reddish yellow on addition of CN^−^ and F^−^ ions through anion-induced deprotonation. It was hence expected that water washing would regenerate the sensor **1** because water serves as a protonating agent and also dissolves CN^−^ and F^−^ ions (from their salts).

**Figure 5:**
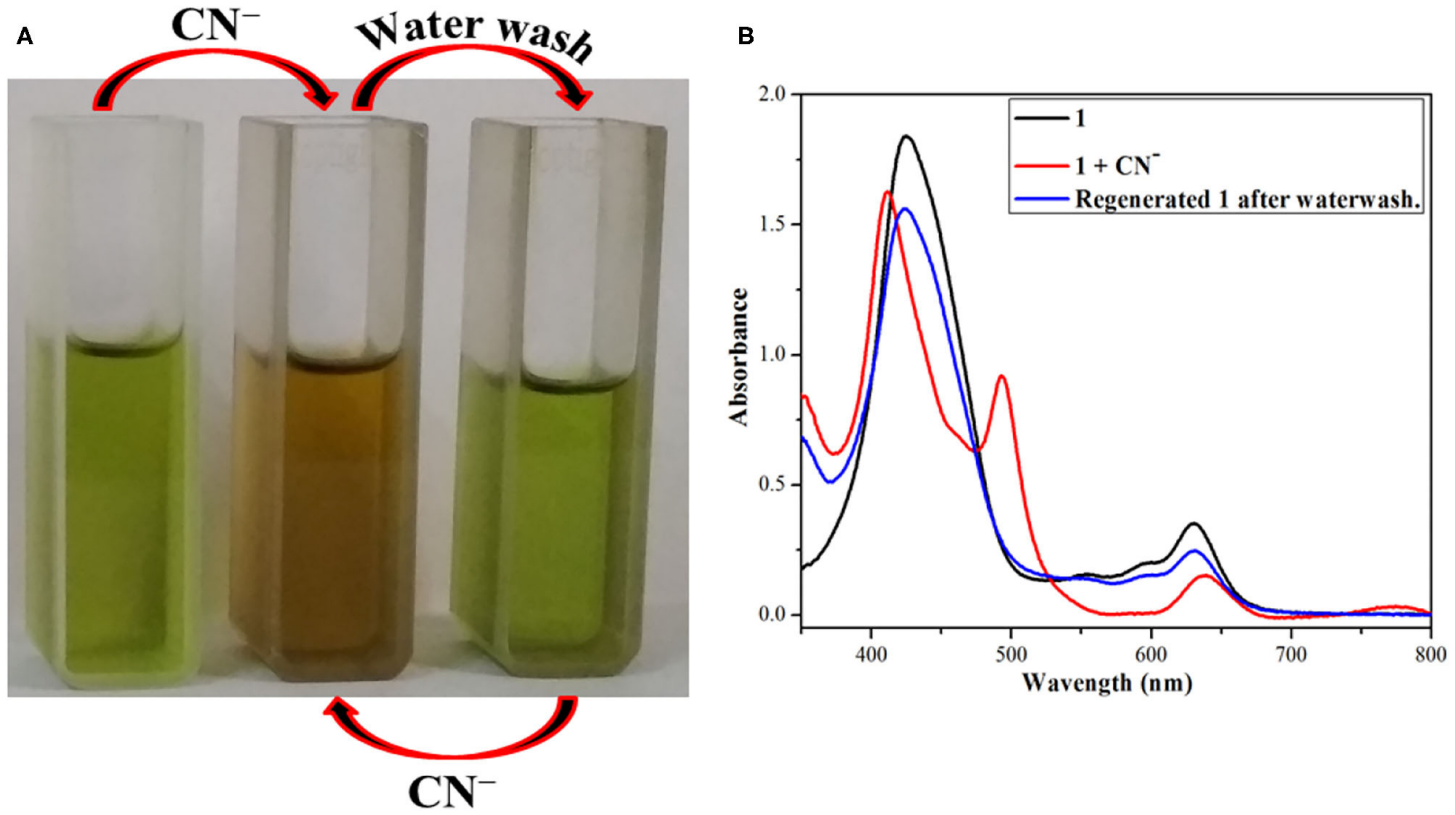
Reversible nature of **1** after addition of CN^−^ ions and followed by water washing **(A)** color changes **(B)** absorption spectral changes using dichloromethane and distilled water at 298 K.

The above changes in color and absorption spectrum of **1** were confirmed after water washing the anion added solution (reddish yellow). Water washing regenerated the original green color of the fused porphyrin solution **1** as shown in Figure 5 and Supplementary Figure 15. The regeneration restored not only the color but also the UV-visible spectrum as the new bands at 410 and 493 nm created due to addition of CN^−^ and F^−^ ions faded away to recreate one single Soret band at 424 nm after washing with water. This regeneration is most certainly due to the fact that deprotonated porphyrin **1**^−^ abstracts a proton from water to regenerate the –NH group and thereby the pure porphyrin **1**. The eminent changes in color and UV-visible spectrum of **1** on adding CN^−^ and F^−^ ions followed by the regeneration of original sensor by water washing make it a reversible probe.

### Computational Studies

B3LYP calculation method along with LANL2DZ basis set was used to optimize the geometry and analyze the electronic structure of **1** and its interaction with CN^−^ and F^−^ ions in CH_2_Cl_2_ (same as the solvent used for experimental studies).

**Figure 6:**
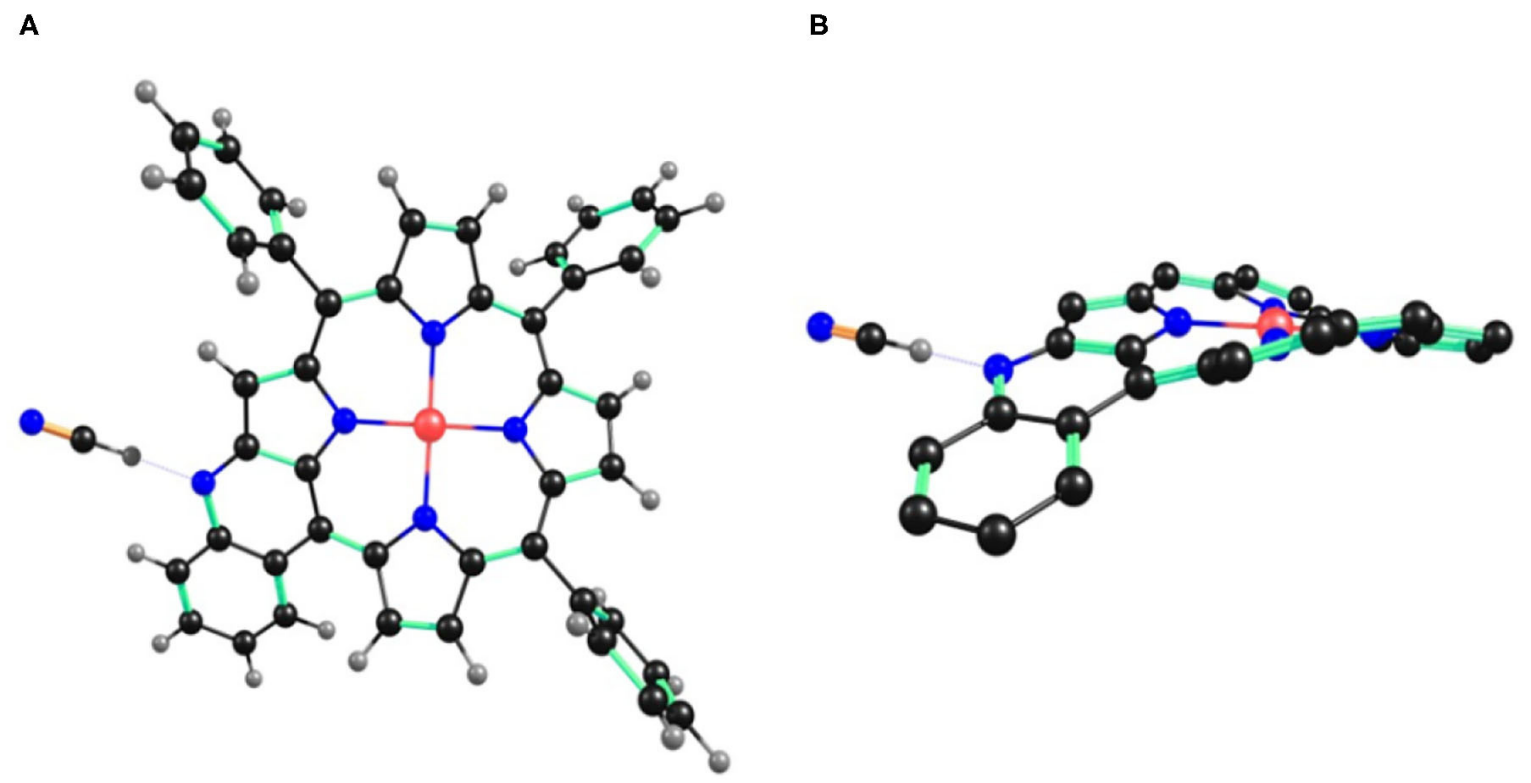
B3LYP/LANL2DZ optimized geometry of **1**•CN^−^ showing **(A)** top view and **(B)** side view. In the side view, all the hydrogen atoms (except fused –NH) and phenyl substituents (except cyclized phenyl) have been removed for clarity.

The optimized structures of **1** revealed that the core is distorted from the mean plane with Δ24 = ±0.2853 Å as shown in Supplementary Figure 16. The core distortion slightly increases in presence of CN^−^ (Δ24 = ±0.2903 Å) and F^−^ (Δ24 = ±0.2893 Å) ions due to aggregation of large negative charge on the fused nitrogen atom (Figure 6 and Supplementary Figure 17). Various optimized structures reveal clear interaction between CN^−^ and F^−^ ions and the fused –NH protons. The fused N-H bond length in **1** is 1.014 Å, which increases to 1.729 and 1.196 Å in presence of CN^−^ and F^−^ ions, respectively.

The increasing N-H bond length in presence of CN^−^ and F^−^ ions is due to interaction of these ions with acidic hydrogen atom of fused –NH, which is finally deprotonated. ΔC_β_ = 0.2044 Å in case of **1** and increases to 0.2122 and 0.2084 Å with CN^−^ and F^−^ ions, respectively. Similarly, Δ${\text{C}}_{\beta^{\prime }}$ = 0.342 Å in case of **1** and increases to 0.394 and 0.369Å with CN^−^ and F^−^ ions, respectively (β′ is the pyrrolic carbon having fused –NH). C_β_-C_β_ bond distance is almost similar in **1** (1.374 Å), **1**•CN^−^ (1.375 Å) and **1**•F^−^ (1.375 Å). However, C_β_-${\text{C}}_{\beta^{\prime }}$ bond distance is highest in case of **1**•CN^−^ (1.407 Å) followed by **1**•F^−^ (1.398 Å) and is shortest in **1** (1.385 Å). These increased bond lengths indicate higher charge density on β′ pyrrolic carbon having fused –NH, which causes more repulsion, thereby increasing the bond length. The frontier molecular orbitals HOMO and LUMO indicate higher electron density in **1**•CN^−^ and **1**•F^−^ than pure probe **1** (Supplementary Figure 18). Thus, DFT studies also support the deprotonation of –NH in case of **1** by CN^−^ and F^−^ ions.

### Sensing Mechanism and Detection Limit

Interaction of CN^−^ and F^−^ ions with porphyrin sensor **1** causes its deprotonation at the fused –NH group, generating a negative charge on the nitrogen atom (–NH peak vanishes in ^1^H-NMR). The negative charge is stabilized *via* conjugation throughout the molecule. This anion-induced deprotonation is responsible for change in color and absorption spectrum of **1**. Deprotonation is the only plausible explanation because no clear alterations in other regions of ^1^H-NMR were noticed and also due to the fact that the changes were reversed on water washing (water is a protic solvent and supplies the necessary proton to the negatively charged nitrogen). On the other hand, anions like CH_3_COO^−^, H_2_${\text{PO}}_{4}^{-}$, Cl^−^, Br^−^, I^−^, ${\text{ClO}}_{4}^{-}$, ${\text{HSO}}_{4}^{-}$, and ${\text{PF}}_{6}^{-}$ do not interact with sensor **1** (or interact weakly) and hence fail to bring about any color or spectral changes. This kind of interaction explains how **1** can preferentially detect toxic CN^−^ and F^−^ ions among the commonly known anions used in this study.

Detection limit was calculated from absorption spectral data on the basis of a reported method (Shortreed et al., 1996). From the performed titration experiments, the absorption data were normalized between highest and lowest absorption values. A linear plot was obtained on the basis of the above data, and the point at which the curve meets the X-axis was taken as the detection limit (Supplementary Figures 19, 20) (Lin et al., 2009). On the basis of this method, **1** exhibited a detection limit of 2.13 and 3.15 ppm for CN^−^ and F^−^ ions, respectively.

### Stoichiometry and Deprotonation Constants

Benesi–Hildebrand plot method was used to predict the stoichiometry and deprotonation constants of **1** with CN^−^ and F^−^ ions from the absorption spectral titration data obtained in distilled CH_2_Cl_2_ at 298 K.

The stoichiometry in case of both CN^−^ and F^−^ ions was found to be 1 on account of linear BH plots obtained from the absorption spectral data (Table 1 and Supplementary Figure 21). The 1:1 stoichiometry was additionally supported by Job's plots (Supplementary Figure 22) obtained in CH_2_Cl_2_ at 298 K.

**Table 1:** Deprotonation constants of **1** with CN^−^ and F^−^ ions in CH_2_Cl_2_ at 298 K.

**Anion**	**log ***β*****	*****β*****	**n^a^**
CN^−^	4.50	3.17 × 10^4^	1
F^−^	2.82	6.71 × 10^2^	1

*n^a^ refers to stoichiometry in CH_2_Cl_2_ at 298 K*.

## Cation Sensing by 2

### UV-Visible Spectral Titration

Absorption spectral titrations of various cations were performed against a standard solution of **2** in distilled CHCl_3_ at 298 K. A stock solution of **2** (1.3 × 10^−5^ M) was prepared in distilled CHCl_3_. Stock solutions of 5.0 × 10^−3^ M concentration of various cations, Ba^+2^, Cd^+2^, Co^+2^, Cu^+2^, Fe^+2^, Fe^+3^, Mg^+2^, Mn^+2^, Hg^+2^, Ni^+2^, Ag^+^, Zn^+2^, and Na^+^, were separately prepared in CH_3_CN (from their perchlorate salts). UV-visible titrations were performed by adding 1-μL aliquots from the various cation solutions to a 3-mL solution of sensor **2** independently. Majority of the cations *viz*. Ba^+2^, Cd^+2^, Co^+2^, Fe^+2^, Mg^+2^, Mn^+2^, Ni^+2^, Ag^+^, Zn^+2^, and Na^+^ failed to bring any noticeable change in the absorption spectrum of **2**. Adding excess amount (10 eq.) of these anions again had no significant bearing on the absorption spectrum of **2** (Supplementary Figures 23–32).

**Figure 7:**
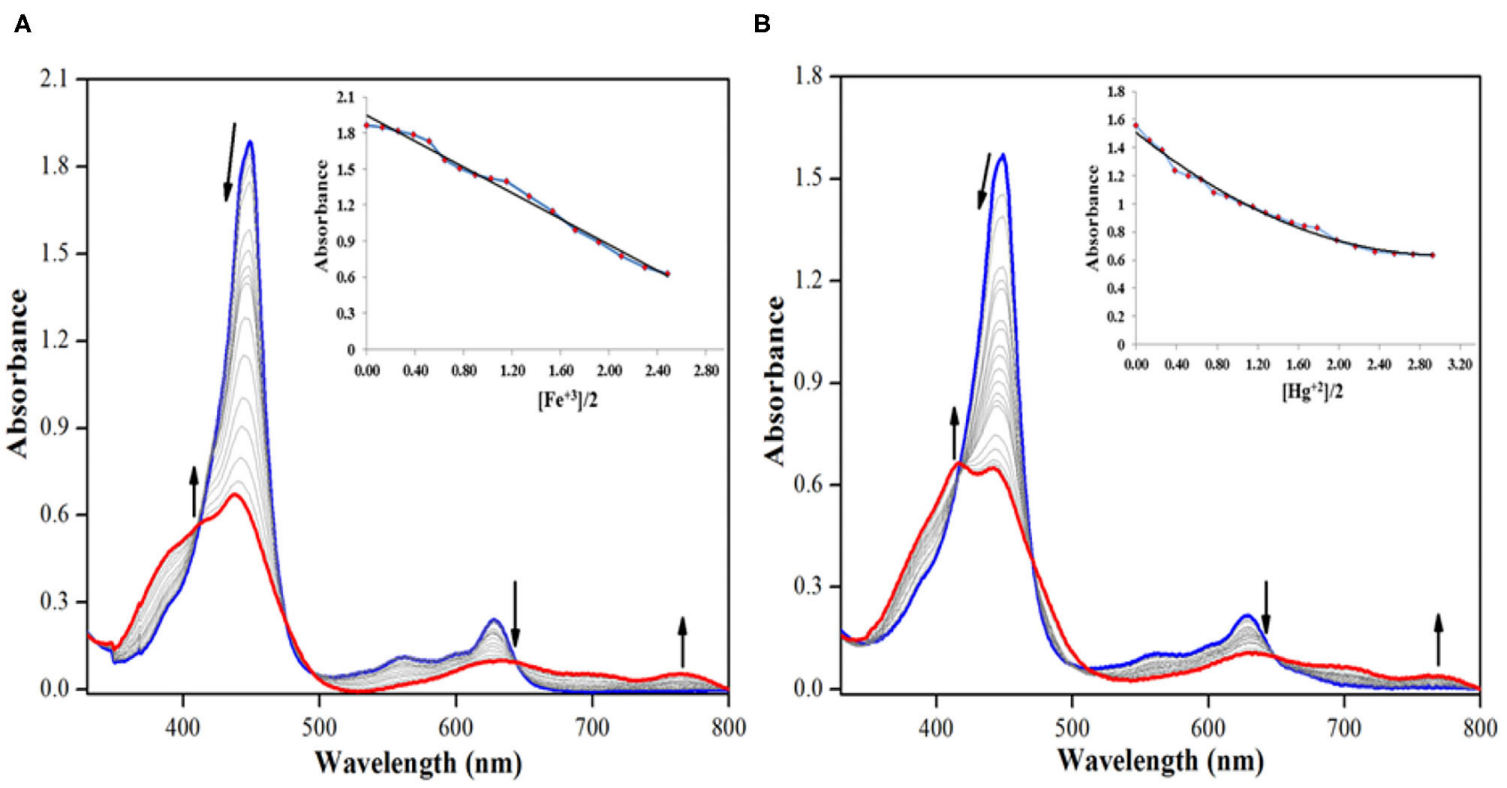
UV-visible spectral titration of **2** on adding **(A)** 0–4.55 × 10^−5^ M, 3.5 equiv. of Fe^+3^ ions and **(B)** 0–4.55 × 10^−5^ M, 3.5 equiv. of Hg^+2^ ions in distilled CHCl_3_ at 298 K. Insets show decrease in absorbance at 447 nm against [cation conc./**2** conc.].

On the other hand, addition of Cu^+2^, Fe^+3^, and Hg^+2^ ions brought significant changes in the absorption spectrum of **2**. These ions interact specifically with the oxygen atom of –CHO group v*ia* columbic interactions causing clear changes in the absorption spectrum of **2** and also inducing color changes from green to red in case of Cu^+2^ and Fe^+3^ ions (not in case of Hg^+2^ ions). On adding roughly 3.5 equiv. of these cations to the neat solution of **2**, the intensity of Soret band at 447 nm decreases accompanied by a small hypsochromic shift. When Fe^+3^ ion was added from 0 to 4.55 × 10^−5^ M (3.5 equiv.), the intensity of Soret band at 447 nm started decreasing accompanied by a minute blue shift. With further additions, the strength and position of the Soret band kept changing, until it was stabilized at 438 nm (Δλ_max_ = 9 nm), along with the creation of a weak shoulder band at 414 nm as shown in Figure 7A. The Q-bands at 557 and 628 nm almost disappeared due to Fe^+3^ addition, and a new weaker band emerged at 764 nm (Figure 7A). With further addition of Fe^+3^ ions, further changes in the spectrum were not observed, and the absorption spectral pattern remained unperturbed. Similar changes in the spectral pattern of **2** were observed on addition of Hg^+2^ ions. On adding 0–4.55 × 10^−5^ M (3.5 equiv.) of Hg^+2^ ions, the Soret band at 447 nm shifted hypsochromically (Δλ_max_ = 5 nm) to 442 nm, and a new shoulder band rose at 417 nm (Figure 7B). Similarly, the Q-bands at 557 and 628 nm almost disappeared due to addition of Hg^+2^ ions, and a new weaker band emerged at 762 nm.

The addition of Cu^+2^ ions to the solution of **2** brought almost identical changes in the UV-visible spectral pattern. On adding 0–4.55 × 10^−5^ M (3.5 equiv.) of Cu^+2^ ions, the Soret band at 447 nm shifted hypsochromically (Δλ_max_ = 9 nm) to 438 nm, and a new shoulder band rose at 415 nm (Supplementary Figure 33). Similarly, the Q-bands at 557 and 628 nm almost disappeared upon the addition of Cu^+2^ ions, and a new weaker band emerged at 762 nm. The observed changes in absorption spectrum of **2** can be attributed to the weak columbic interactions between the metal cations and the lone pair on oxygen atom of the formyl group (on sensor **2**).

Considering that absorption spectral changes were observed only in case of Cu^+2^, Fe^+3^, and Hg^+2^ ions and not in case of Ba ^+2^, Cd^+2^, Co^+2^, Fe^+2^, Mg^+2^, Mn^+2^, Ni^+2^, Ag^+^, Zn^+2^, and Na^+^ ions, hence **2** can be selectively used to detect Cu^+2^, Fe^+3^, and toxic Hg^+2^ ions from the above assortment of cations. While Cu^+2^ and Fe^+3^ ions can be detected spectrometrically as well as colorimetrically, Hg^+2^ can be detected only spectrometrically. As sensor **2** is non-fluorescent, hence fluorescence titration of **2** with different cations was not possible. The addition of different cations did not enhance the fluorescence of sensor **2**, which continued to remain non-fluorescent.

### ^1^H-NMR Titration

**Figure 8:**
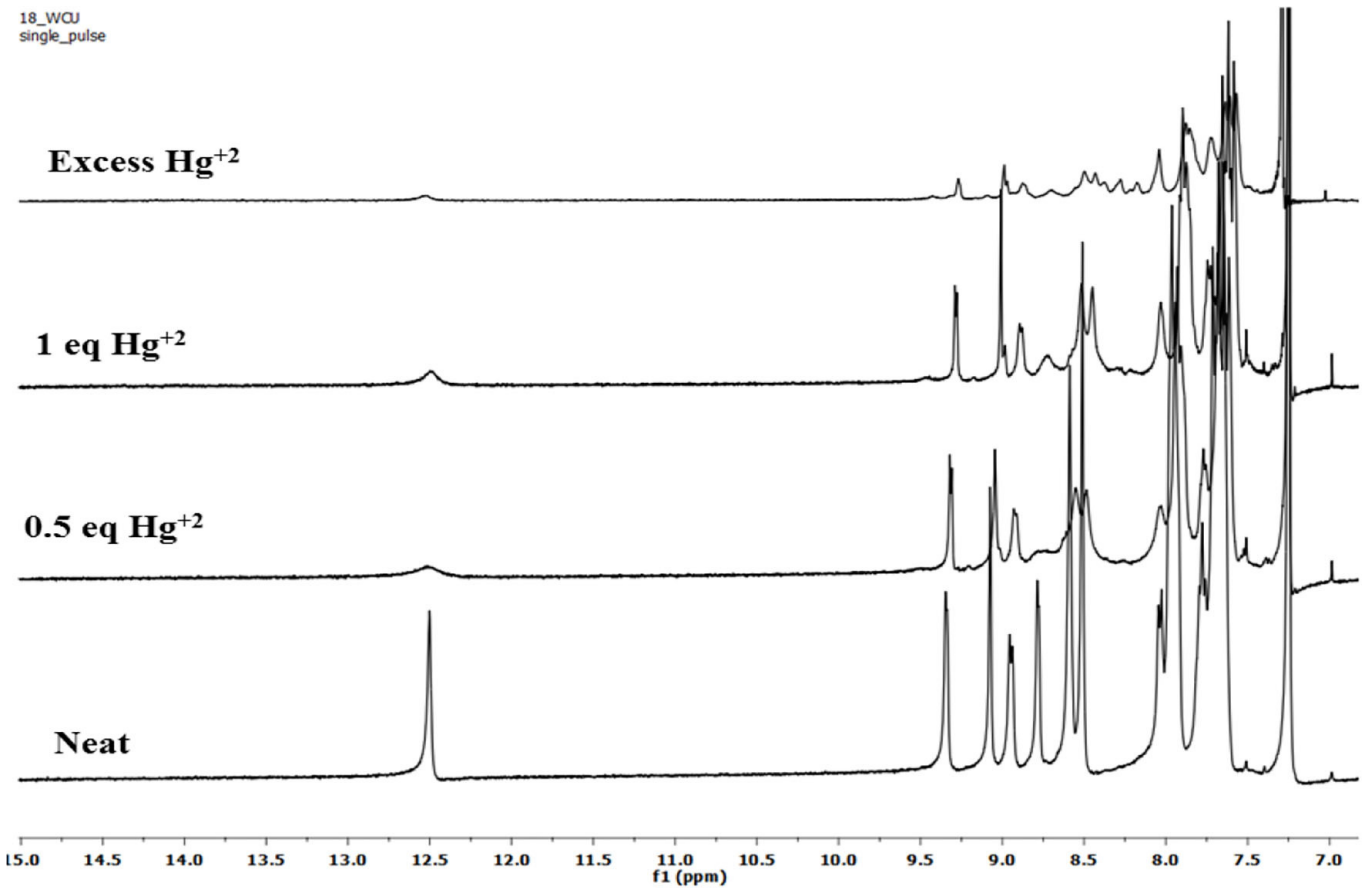
^1^H-NMR titration of **2** (in CDCl_3_) with Hg^+2^ ions (in DMSO-d_6_) at 298 K.

To account for the mechanism responsible for the affected spectral changes (due to cation addition), ^1^H-NMR titration of **2** (dissolved in CDCl_3_) with Cu^+2^, Fe^+3^, and Hg^+2^ ions (dissolved in DMSO-d_6_) was performed at 298 K. When neat, porphyrin **2** shows fused –NH protons at 12.50 ppm and –CHO protons at 9.07 ppm. Addition of 0.5 equiv. Hg^+2^ ions to **2** decreased the intensity of different peaks without changing the peak positions. When the amount of Hg^+2^ added was increased to 1 equiv., still no changes in the peak positions were observed including –NH and –CHO peaks. On adding excess amount of Hg^+2^ ions (5 equiv.), again no change in different peaks was observed other than their decreased intensity as shown in Figure 8. The unchanged ^1^H-NMR spectrum of **2** upon addition of Hg^+2^ ions indicates that no major chemical or structural change takes place on adding the cation solution to it.

^1^H-NMR titrations of **2** with Cu^+2^ and Fe^+3^ ions were not very clear because of their paramagnetic nature. However, the peak positions reflect no change upon addition of these cations to porphyrin **2** (Supplementary Figures 34, 35). These ^1^H-NMR titrations indicate that the interaction between the added cations and the porphyrin **2** is weak attraction between metal cations having high positive charge density and oxygen atom on formyl having a lone pair of electrons. The weak interactions were confirmed by water washing, which caused reversal in spectral changes, which would not have been possible in case of strong complexation.

### Colorimetric Studies and Naked-Eye Detection

**Figure 9:**
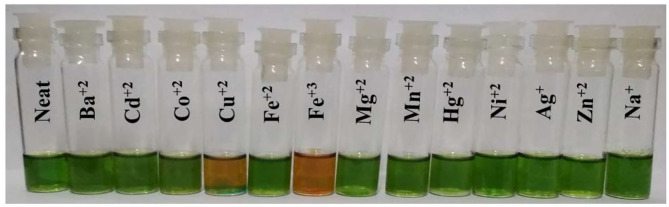
Colorimetric studies of **2** using 4 equiv. of various cations in CHCl_3_ at 298 K.

Colorimetric studies were carried out to study the effect of cation addition on the color of fused porphyrin solution **2** in chloroform. For performing the colorimetric studies, 15 μM solution of **2** was prepared in distilled CHCl_3_. Equal sized sample vials were filled with roughly 1 mL of the prepared solution. One of the solutions was kept pure, and to the remaining sample vials, 4 equivalents of different cations (prepared in CH_3_CN) were added. A vivid color change from clear green to reddish brown was exhibited by solutions to which Cu^+2^ and Fe^+3^ ions were added as shown in Figure 9. The color changes were not observed with Hg^+2^ ions probably due to *d*^10^ electronic configuration. Nonetheless, **2** can be used for detecting Hg^+2^ ions spectrometrically as the absorption spectrum of **2** exhibited a clear change due to Hg^+2^ ion addition. This cation-affected color change can be used to empirically detect Cu^+2^ and Fe^+3^ ions with naked eyes.

### Computational Studies

B3LYP functional along with LANL2DZ basis set was used to analyze the geometry and electronic structure of **2** and its interaction with Cu^+2^, Fe^+3^, and Hg^+2^ ions in CHCl_3_ (same as the solvent used for experimental studies).

**Figure 10:**
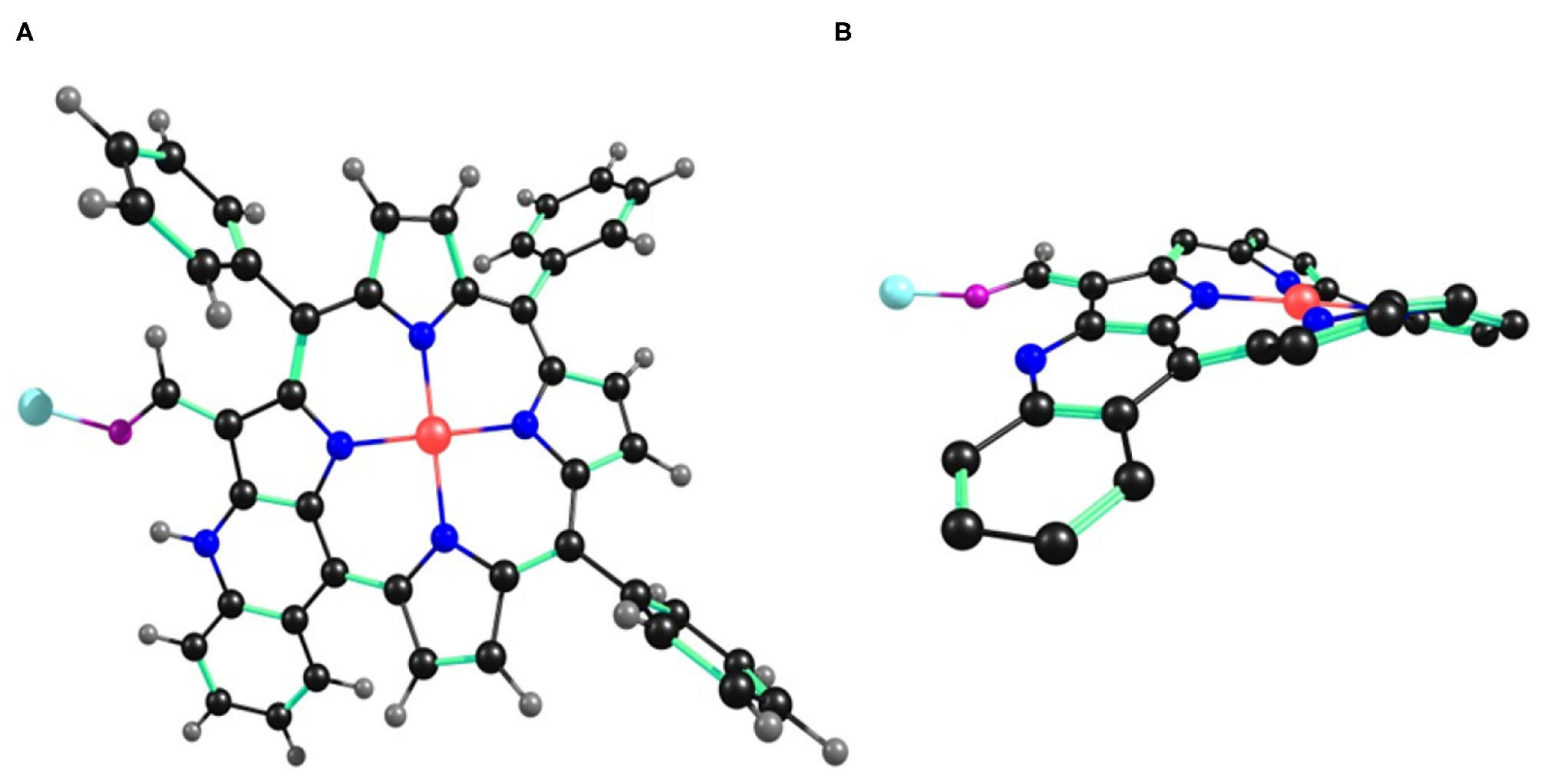
B3LYP/LANL2DZ optimized geometry of **2**•Cu^+2^ showing **(A)** top view and **(B)** side view. In the side view, all the hydrogen atoms (except –CHO) and phenyl substituents (except cyclized phenyl) have been removed for clarity.

The optimized structure of **2** reveals that the macrocyclic core is distorted from the mean plane (Δ24 = ±0.3052 Å), as shown in Supplementary Figure 36. The core distortion increases in presence of Cu^+2^ (Δ24 = ±0.3693 Å), Fe^+3^ (Δ24 = ±0.3453 Å), and Hg^+2^ (Δ24 = ±0.3542 Å) ions (Figure 10 and Supplementary Figures 37, 38). Various optimized structures reveal clear interaction between Cu^+2^, Fe^+3^, and Hg^+2^ ions and the formyl oxygen atom on **2**. The formyl group –C=O bond distance in **2** is 1.281 Å, which increases to 1.306, 1.321, and 1.292 Å in case Cu^+2^, Fe^+3^, and Hg^+2^ ions, respectively. The increasing –C=O bond length is a clear indication that Cu^+2^, Fe^+3^, and Hg^+2^ ions interact with oxygen atom of –CHO group taking away some of its electron density. ΔC_β_ = 0.2315 Å in case of **2** and increases to 0.370, 0.268, and 0.2833 Å in presence of Cu^+2^, Fe^+3^, and Hg^+2^ ions, respectively. Similarly, ΔCβ′ = 0.381 Å in case of **2** and increases to 0.606, 0.474, and 0.575 Å with Cu^+2^, Fe^+3^, and Hg^+2^ ions, respectively (β′ is the pyrrolic carbon having fused –NH). However, Δ${\text{C}}_{\beta^{{}^{\prime \prime }}}$ = 0.132 Å in case of **2** and decreases to 0.100, 0.051, and 0.022 Å with Cu^+2^, Fe^+3^, and Hg^+2^ ions, respectively (β″ is the pyrrolic carbon having –CHO) because some of the electron density moves away from the core toward the metal cations. C_β_-C_β_ bond distance is almost similar in **2** (1.374 Å), **2**•Cu^+2^ (1.373 Å), **2**•Fe^+3^ (1.368 Å), and **2**•Hg^+2^ (1.374 Å). However, ${\text{C}}_{\beta^{\prime }}$-${\text{C}}_{\beta^{{}^{\prime \prime }}}$ bond distance is highest in **2**•Hg^+2^ (1.427 Å), followed by **2**•Cu^+2^ (1.426 Å) and **2**•Fe^+3^ (1.421 Å), respectively. ${\text{C}}_{\beta^{\prime }}$-${\text{C}}_{\beta^{{}^{\prime \prime }}}$ bond distance is shortest in **2** (1.409 Å). The frontier molecular orbitals HOMO and LUMO are shown in Supplementary Figures 39, 40. Thus, DFT studies also support the weak interaction between **2** and various cations (Cu^+2^, Fe^+3^, and Hg^+2^).

### Mechanism and Detection Limit

Addition of Cu^+2^, Fe^+3^, and Hg^+2^ ions to the solution of **2** cause clear changes in the absorption spectrum of **2**. The possible explanation is the interaction between oxygen atom (having lone pair of electrons) of –CHO group on **2** and the metal cations that are electron deficient. This mechanism is supported by DFT studies, as well as ^1^H-NMR titration. Water washing reverses the interactions, which means they are weakly attractive in nature.

Detection limit was calculated from absorption spectral data on the basis of the method discussed for anions above (Supplementary Figures 41–43). On the basis of this method, **2** exhibited a detection limit of 0.930, 2.23, and 0.718 ppm for Cu^+2^, Fe^+3^, and Hg^+2^ ions, respectively.

### Stoichiometry and Binding Constants

BH plot method was used to predict the stoichiometry and binding constants of **2** with Cu^+2^, Fe^+3^, and Hg^+2^ ions from the absorption titration data obtained in distilled CHCl_3_ at 298 K. The stoichiometry in case of Cu^+2^, Fe^+3^, and Hg^+2^ ions was found to be 1 on account of linear BH plots obtained from the absorption spectral data (Table 2 and Supplementary Figures 44–46).

**Table 2:** Binding constants of **2** with Cu^+2^, Fe^+3^, and Hg^+2^ ions in CHCl_3_ at 298 K.

**Cation**	**log ***β*****	*****β*****	**n^**a**^**
Cu^+2^	4.43	2.7 × 10^4^	1
Fe^+3^	4.71	5.2 × 10^4^	1
Hg^+2^	4.68	4.8 × 10^4^	1

*n^a^refers to stoichiometry in distilled CHCl_3_ at 298 K*.

## Conclusions

Ni(II) porphyrins having fused –NH group (**1** and **2**) have been synthesized and characterized by various spectroscopic techniques. The synthesized Ni(II) porphyrins **1** and **2** were utilized to detect species of opposite polarity. **1** was used to sense toxic anions *viz*. cyanide and fluoride whereas **2** was utilized for detecting some selective metal ions including toxic Hg(II) ions. **1** possesses acidic –NH protons and detects anions *via* hydrogen bonding interaction followed by anion-induced deprotonation; **2**, on the other hand, senses the metal species *via* weak charge transfer interactions between oxygen atom of formyl group and the metal atoms. Considering that both the sensors have only one reactive site through which they can interact with their respective analytes, hence saturation point in various titrations was achieved with almost similar concentrations of analytes (~4 equivalents for both **1** and **2**). The stoichiometry of complexation was found to be 1. The detection limit in case of **1** was found to be 2.13 ppm for cyanide and 3.15 ppm for fluoride ions, respectively. Similarly, the detection limits were found to be 0.930, 2.231, and 0.718 ppm for Cu(II), Fe(III), and Hg(II) ions, respectively for probe **2**. The sensors are recoverable and reusable for numerous cycles.

## Data Availability Statement

The original contributions presented in the study are included in the article/Supplementary Materials, further inquiries can be directed to the corresponding author.

## Author Contributions

TD performed the synthesis, characterization and purification of the molecular sensors, performed other experiments regarding sensing and various spectrometric titrations, and wrote the first draft of the manuscript. MS made significant contributions to data analysis and made valuable suggestions regarding editing of the final manuscript. Both authors contributed to the article and approved the submitted version.

## Conflict of Interest

The authors declare that the research was conducted in the absence of any commercial or financial relationships that could be construed as a potential conflict of interest.
