# Supplementary material for: Fused Nickel(II) Porphyrins—Sensing of Toxic Anions and Selected Metal Ions Through Supramolecular Interactions
Source: Front Chem. 2020 Nov 17;8:595177. doi: 10.3389/fchem.2020.595177 (PMC7705245; doi:10.3389/fchem.2020.595177)
Supplement: Supplementary file 1 [file Table_1.DOC]

Supplementary Material

| **Table of contents** | **Page No.** |
| --- | --- |
| **Supplementary Figure S1.** ^1^H NMR spectrum of fused –NH Porphyrin **1** in CDCl_3_ at 298 K. | 5 |
| **Supplementary Figure S2.** ^1^H NMR spectrum of fused formyl Porphyrin **2** in CDCl_3_ at 298 K. | 5 |
| **Supplementary Figure S3.** UV-Visible spectra of **1** and **2** in CH_2_Cl_2_ at 298 K. | 6 |
| **Supplementary Figure S4.** UV-Visible spectral titration of fused –NH porphyrin **1** with CH_3_COO^‒^ in CH_2_Cl_2_ at 298 K (adding 0-1.11×10 ̶ 3 M, 50 equiv. CH_3_COO^‒^). | 7 |
| **Supplementary Figure S5.** UV-Visible spectral titration of fused –NH porphyrin **1** with HSO_4_^‒^ in CH_2_Cl_2_ at 298 K (adding 0-1.11×10 ̶ 3 M, 50 equiv. HSO_4_^‒^). | 7 |
| **Supplementary Figure S6**. UV-Visible spectral titration of fused –NH porphyrin **1** with Br^‒^ in CH_2_Cl_2_ at 298 K (adding 0-1.11×10 ̶ 3 M, 50 equiv. Br^‒^). | 8 |
| **Supplementary Figure S7.** UV-Visible spectral titration of fused –NH porphyrin **1** with Cl^‒^ in CH_2_Cl_2_ at 298 K (adding 0-1.11 × 10 ̶ 3 M, 50 equiv. Cl^‒^). | 8 |
| **Supplementary Figure S8.** UV-Visible spectral titration of fused –NH porphyrin **1** with I^‒^ in CH_2_Cl_2_ at 298 K (adding 0-1.11×10 ̶ 3 M, 50 equiv. I^‒^). | 9 |
| **Supplementary Figure S9.** UV-Visible spectral titration of fused –NH porphyrin **1** with ClO_4_^‒^ in CH_2_Cl_2_ at 298 K (adding 0-1.11×10 ̶ 3 M, 50 equiv. ClO_4_^‒^). | 9 |
| **Supplementary Figure S10.** UV-Visible spectral titration of fused –NH porphyrin **1** with PF_6_^‒^ in CH_2_Cl_2_ at 298 K (adding 0-1.11×10 ̶ 3 M, 50 equiv. PF_6_^‒^). | 10 |
| **Supplementary Figure S11.** UV-Visible spectral titration of fused –NH porphyrin **1** with H_2_PO_4_^‒^ in CH_2_Cl_2_ at 298 K (adding 0-1.11×10 ̶ 3 M, 50 equiv. H_2_PO_4_^‒^). | 10 |
| **Supplementary Figure S12.** UV-Visible spectral titration of **1** on adding 0-7.80×10^‒5^ M, 3.5 equiv. equiv. OH^−^ ions in distilled CH_2_Cl_2_ at 298 K. Inset shows decrease in absorbance at 424 nm and the concomitant increase in absorbance at 493 nm against [OH^−^/**1** conc.] | 11 |
| **Supplementary Figure S13.**^1^H NMR titration of **1** with F^‒^ ions in CDCl_3_ at 298 K. | 11 |
| **Supplementary Figure S14.**^1^H NMR titration of **1** with OH^‒^ ions in CDCl_3_ at 298 K. | 12 |
| **Supplementary Figure S15.** Reversible nature of **1** after adding F^‒^ ions and water washing in terms of (a) color changes (b) UV-Visible spectral changes using CH_2_Cl_2_ and distilled water at 298 K. | 12 |
| **Supplementary Figure S16**. B3LYP/LANL2DZ set optimized geometry of **1** showing (a) top view and (b) side view. In the side view all the hydrogen atoms (except fused -NH) and phenyl substituents (except cyclized phenyl) have been removed for clarity. | 13 |
| **Supplementary Figure S17**. B3LYP/LANL2DZ set optimized geometry of **1**•F^−^ showing (a) top view and (b) side view. In the side view all the hydrogen atoms (except fused -NH) and phenyl substituents (except cyclized phenyl) have been removed for clarity. | 13 |
| **Supplementary Figure S18.** DFT optimized geometries showing HOMO and LUMO orbitals in **1**, **1**•CN^−^ and **1**•F^−^. | 14 |
| **Supplementary Figure S19.** Absorbance of **1** in CH_2_Cl_2_ normalized between maximum absorbance value at zero CN^−^ conc. and minimum absorbance value at 7 equiv. (1.56×10^‒4^ M) of CN^−^ ions (for calculating detection limit). | 14 |
| **Supplementary Figure S20.** Absorbance of **1** in CH_2_Cl_2_ normalized between maximum absorbance value at zero F^−^ conc. and minimum absorbance value at 7 equiv. (1.56×10^‒4^ M) of F^−^ ions (for calculating detection limit). | 15 |
| **Supplementary Figure S21.** BH Plots obtained from UV-Visible spectral titration of **1** with (a) CN^−^ and (b) F^−^ ions in distilled CH_2_Cl_2_ at 298 K indicating 1:1 stoichiometry between **1** and the added anions. | 15 |
| **Supplementary Figure S22.** Job’s plot for (a) CN^−^ ─ **1** (b) F^−^ ─ **1** interactions in distilled CH_2_Cl_2_ at 298 K indicating 1:1 stoichiometry. Total concentration of ([anion] + [**1**]) = 2.23×10^–5^ M. | 16 |
| **Supplementary Figure S23.** UV-Visible spectral titration of fused –CHO porphyrin **2** with Ba^+2^ in CHCl_3_ at 298 K (adding 0-1.3×10 ̶ 4 M, 10 equiv. Ba^+2^). | 16 |
| **Supplementary Figure S24.** UV-Visible spectral titration of fused –CHO porphyrin **2** with Cd^+2^ in CHCl_3_ at 298 K (adding 0-1.3×10 ̶ 4 M, 10 equiv. Cd^+2^). | 17 |
| **Supplementary Figure S25.** UV-Visible spectral titration of fused –CHO porphyrin **2** with Co^+2^ in CHCl_3_ at 298 K (adding 0-1.3×10 ̶ 4 M, 10 equiv. Co^+2^). | 17 |
| **Supplementary Figure S26.** UV-Visible spectral titration of fused –CHO porphyrin **2** with Fe^+2^ in CHCl_3_ at 298 K (adding 0-1.3×10 ̶ 4 M, 10 equiv. Fe^+2^). | 18 |
| **Supplementary Figure S27.** UV-Visible spectral titration of fused –CHO porphyrin **2** with Mg^+2^ in CHCl_3_ at 298 K (adding 0-1.3×10 ̶ 4 M, 10 equiv. Mg^+2^). | 18 |
| **Supplementary Figure S28.** UV-Visible spectral titration of fused –CHO porphyrin **2** with Mn^+2^ in CHCl_3_ at 298 K (adding 0-1.3 × 10 ̶ 4 M, 10 equiv. Mn^+2^). | 19 |
| **Supplementary Figure S29.** UV-Visible spectral titration of fused –CHO porphyrin **2** with Ni^+2^ in CHCl_3_ at 298 K (adding 0-1.3×10 ̶ 4 M, 10 equiv. Ni^+2^). | 19 |
| **Supplementary Figure S30.** UV-Visible spectral titration of fused –CHO porphyrin **2** with Ag^+^ in CHCl_3_ at 298 K (adding 0-1.3×10 ̶ 4 M, 10 equiv. Ag^+^). | 20 |
| **Supplementary Figure S31.** UV-Visible spectral titration of fused –CHO porphyrin **2** with Zn^+2^ in CHCl_3_ at 298 K (adding 0-1.3 × 10 ̶ 4 M, 10 equiv. Zn^+2^). | 20 |
| **Supplementary Figure S32.** UV-Visible spectral titration of fused –CHO porphyrin **2** with Na^+^ in CHCl_3_ at 298 K (adding 0-1.3×10 ̶ 4 M, 10 equiv. Na^+^). | 21 |
| **Supplementary Figure S33.** UV-Visible spectral titration of **2** on adding (a) 0-4.55×10^-5^ M, 3.5 equiv. of Cu^+2^ ions in distilled CHCl_3_ at 298 K. Insets show decrease in absorbance at 447 nm against [cation conc.]/[**2** conc.]. | 21 |
| **Supplementary Figure S34.**^1^H NMR titration of **2** (in CDCl_3_) with Cu^+2^ ions (in DMSO-d_6_) at 298 K. | 22 |
| **Supplementary Figure S35.**^1^H NMR titration of **2** (in CDCl_3_) with Fe^+3^ ions (in DMSO-d^6^) at 298 K. | 22 |
| **Supplementary Figure S36.** B3LYP/LANL2DZ set optimized geometry of **2** showing (a) top view and (b) side view. In the side view all the hydrogen atoms (except -CHO) and phenyl substituents (except cyclized phenyl) have been removed for clarity. | 23 |
| **Supplementary Figure S37.** B3LYP/LANL2DZ set optimized geometry of **2**•Fe^+3^ showing (a) top view and (b) side view. In the side view all the hydrogen atoms (except -CHO) and phenyl substituents (except cyclized phenyl) have been removed for clarity. | 23 |
| **Supplementary Figure S38.** B3LYP/LANL2DZ set optimized geometry of **2**•Hg^+2^ showing (a) top view and (b) side view. In the side view all the hydrogen atoms (except -CHO) and phenyl substituents (except cyclized phenyl) have been removed for clarity. | 24 |
| **Supplementary Figure S39.** DFT optimized geometries showing HOMO and LUMO orbitals in **2**. | 24 |
| **Supplementary Figure S40**. DFT optimized geometries showing HOMO and LUMO orbitals in **2**•Cu^+2^, **2**•Fe^+3^ and **2**•Hg^+2^ respectively. | 25 |
| **Supplementary Figure S41.** Absorbance of **2** in CHCl_3_ normalized between maximum absorbance value at zero Cu^+2^ conc. and minimum absorbance value at 3.5 equiv. (4.55×10^-5^M)of Cu^+2^ ions (for calculating detection limit). | 25 |
| **Supplementary Figure S42.** Absorbance of **2** in CHCl_3_ normalized between maximum absorbance value at zero Fe^+3^ conc. and minimum absorbance value at 3.5 equiv. (4.55×10^-5^M)of Fe^+3^ ions (for calculating detection limit). | 26 |
| **Supplementary Figure S43.** Absorbance of **2** in CHCl_3_ normalized between maximum absorbance value at zero Hg^+2^ conc. and minimum absorbance value at 3.5 equiv. (4.55×10^-5^M)of Hg^+2^ ions (for calculating detection limit). | 26 |
| **Supplementary Figure S44.** BH Plot obtained from UV-Visible spectral titration of **2** with Cu^+2^ ions in distilled CHCl_3_ at 298 K indicating 1:1 stoichiometry. | 27 |
| **Supplementary Figure S45.** BH Plot obtained from UV-Visible spectral titration of **2** with Fe^+3^ ions in distilled CHCl_3_ at 298 K indicating 1:1 stoichiometry. | 27 |
| **Supplementary Figure S46.** BH Plot obtained from UV-Visible spectral titration of **2** with Hg^+2^ ions in distilled CHCl_3_ at 298 K indicating 1:1 stoichiometry. | 28 |
| **Supplementary Table S1.** UV-Visible spectral data of **1** and **2** in CH_2_Cl_2_ at 298 K. | 6 |


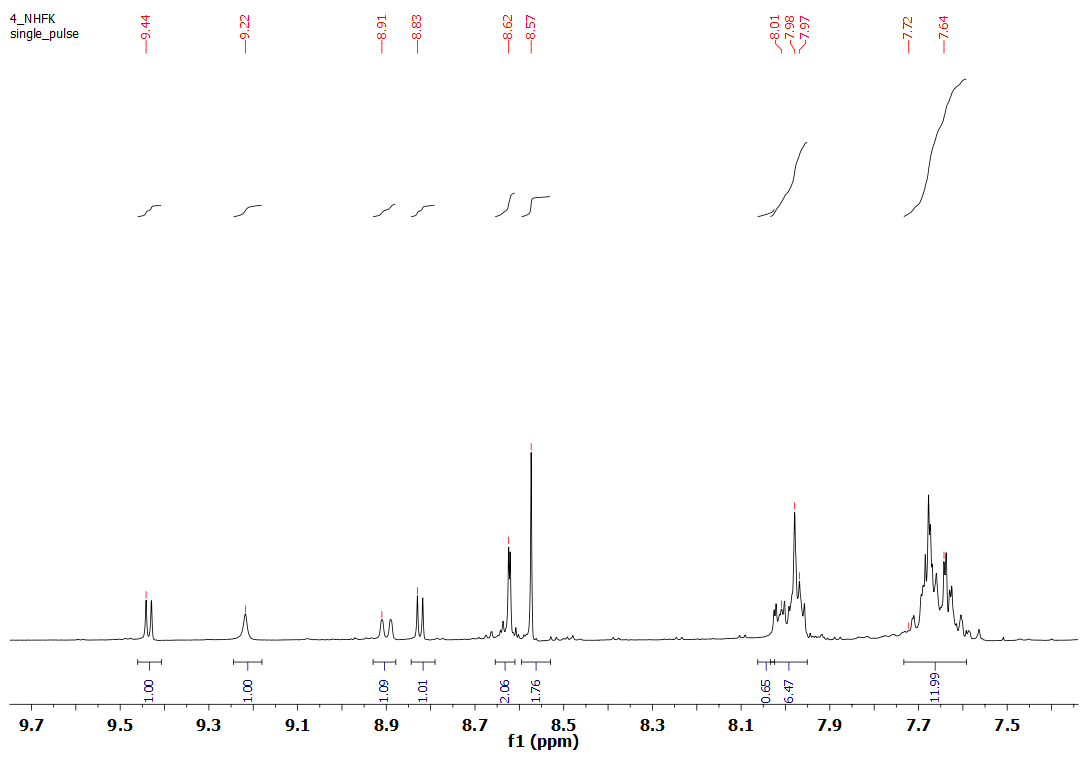


**Supplementary Figure S1:** ^1^H NMR spectrum of fused –NH Porphyrin **1** in CDCl_3_ at 298 K.


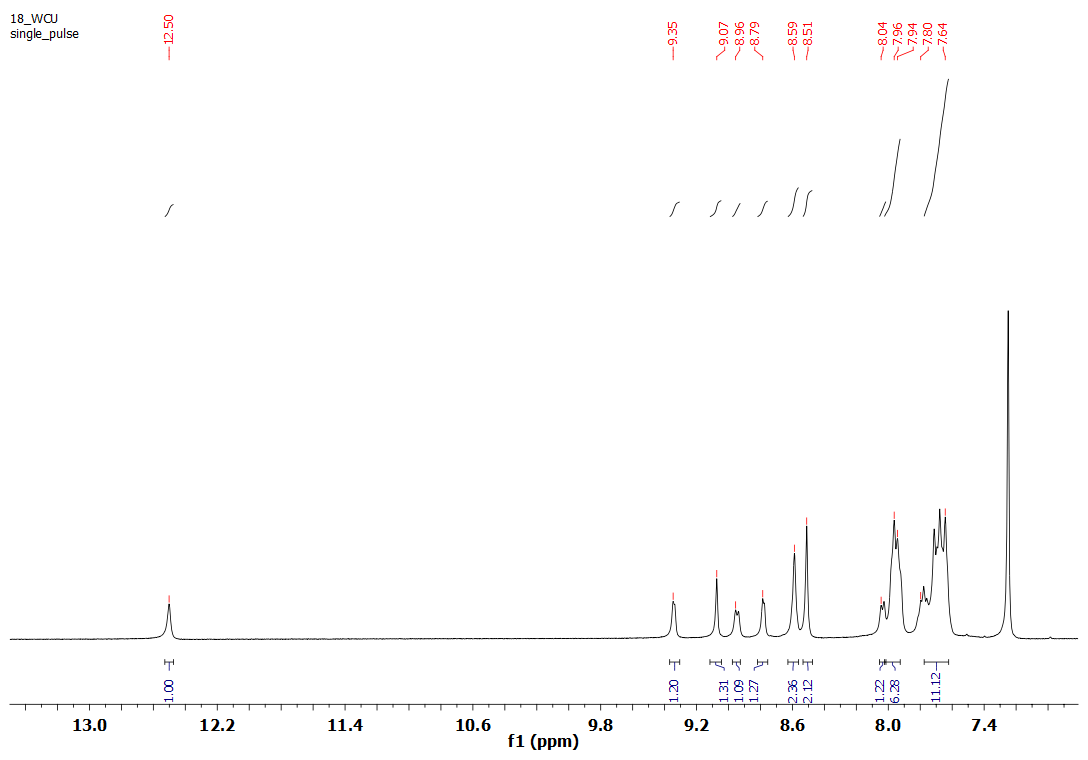


**Supplementary Figure S2:** ^1^H NMR spectrum of fused formyl Porphyrin **2** in CDCl_3_ at 298 K.

**Supplementary Table S1:**  UV-Visible spectral data of **1** and **2** in CH_2_Cl_2_ at 298 K.

| **Compound** | **B Band(s),nm** | **Q band(s), nm** |
| --- | --- | --- |
| Fused –NH porphyrin (1) | 424 | 552, 596, 628. |
| Fused –CHO porphyrin (2) | 447 | 557,628. |


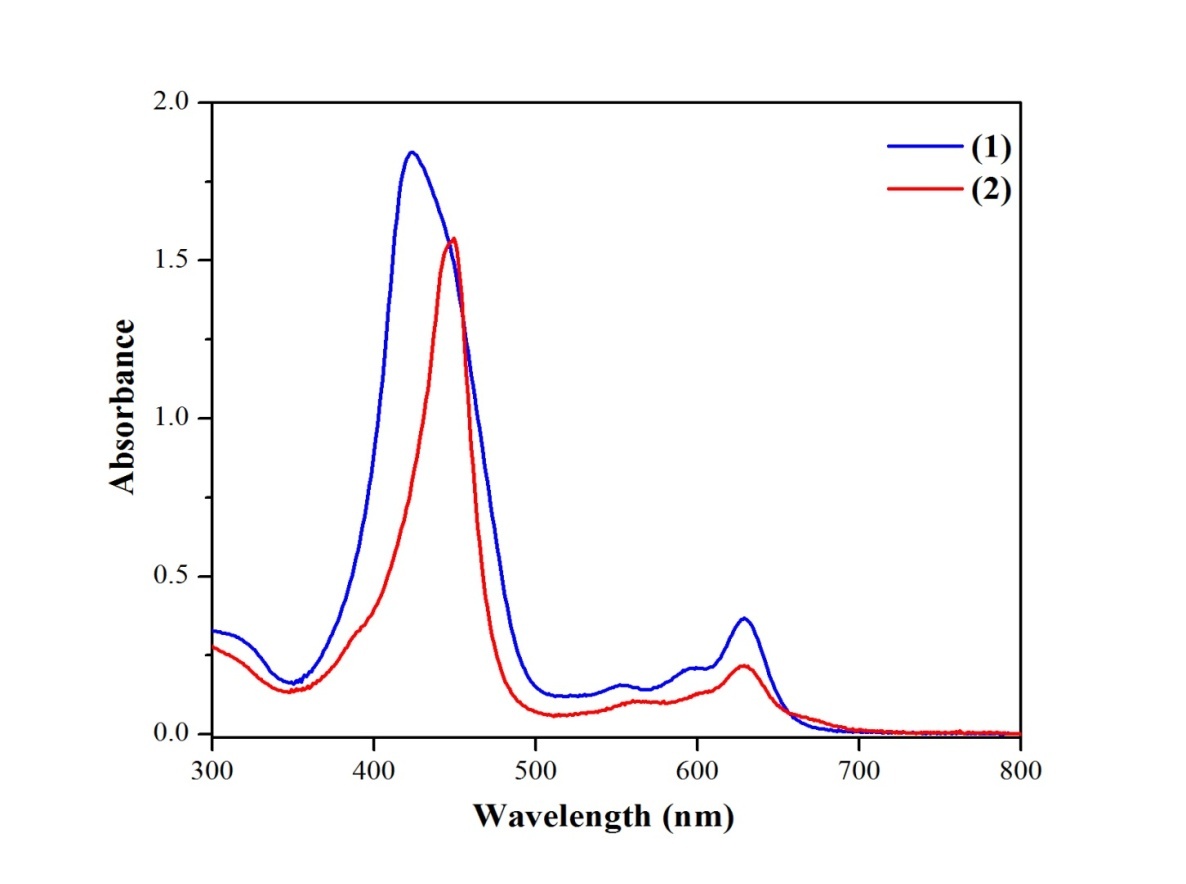


**Supplementary Figure S3:** UV-Visible spectra of **1** and **2** in CH_2_Cl_2_ at 298 K.


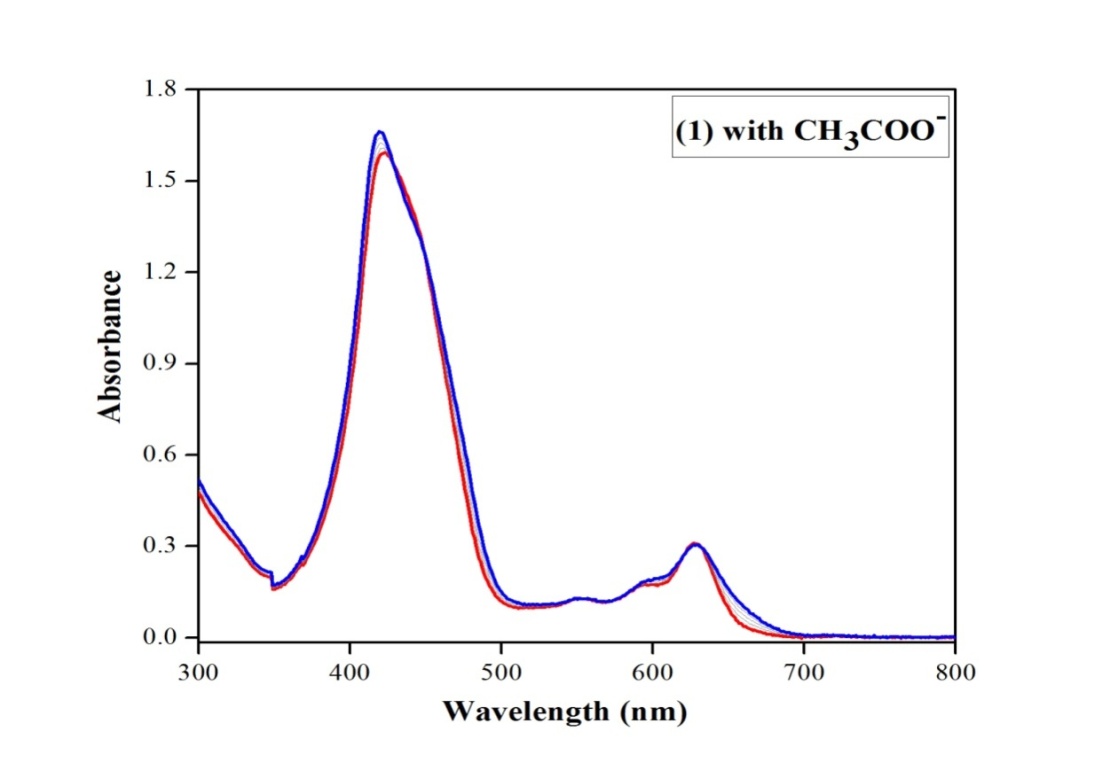


**Supplementary Figure S4:** UV-Visible spectral titration of fused –NH porphyrin (**1**) with CH_3_COO^‒^ in CH_2_Cl_2_ at 298 K (adding 0-1.11×10 ̶ 3 M, 50 equiv. CH_3_COO^‒^).


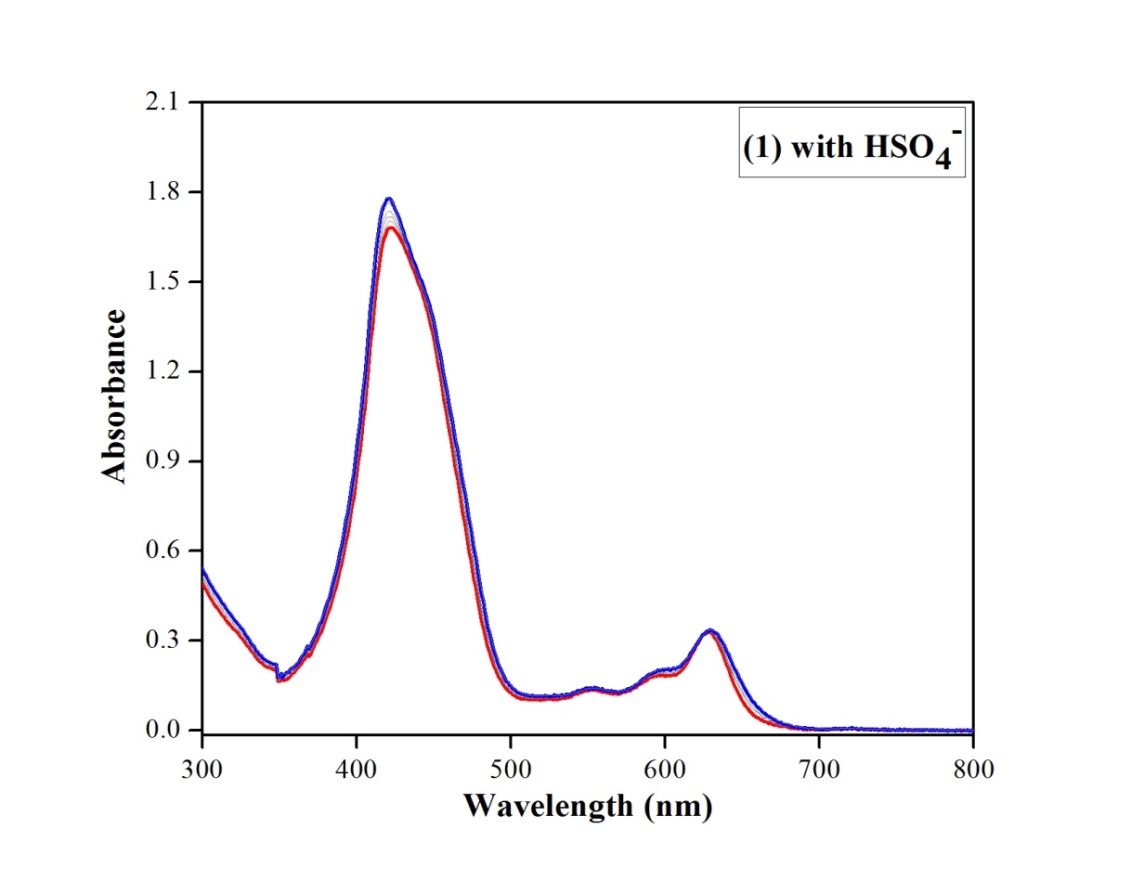


**Supplementary Figure S5:** UV-Visible spectral titration of fused –NH porphyrin (**1**) with HSO_4_^‒^ in CH_2_Cl_2_ at 298 K (adding 0-1.11×10 ̶ 3 M, 50 equiv. HSO_4_^‒^).


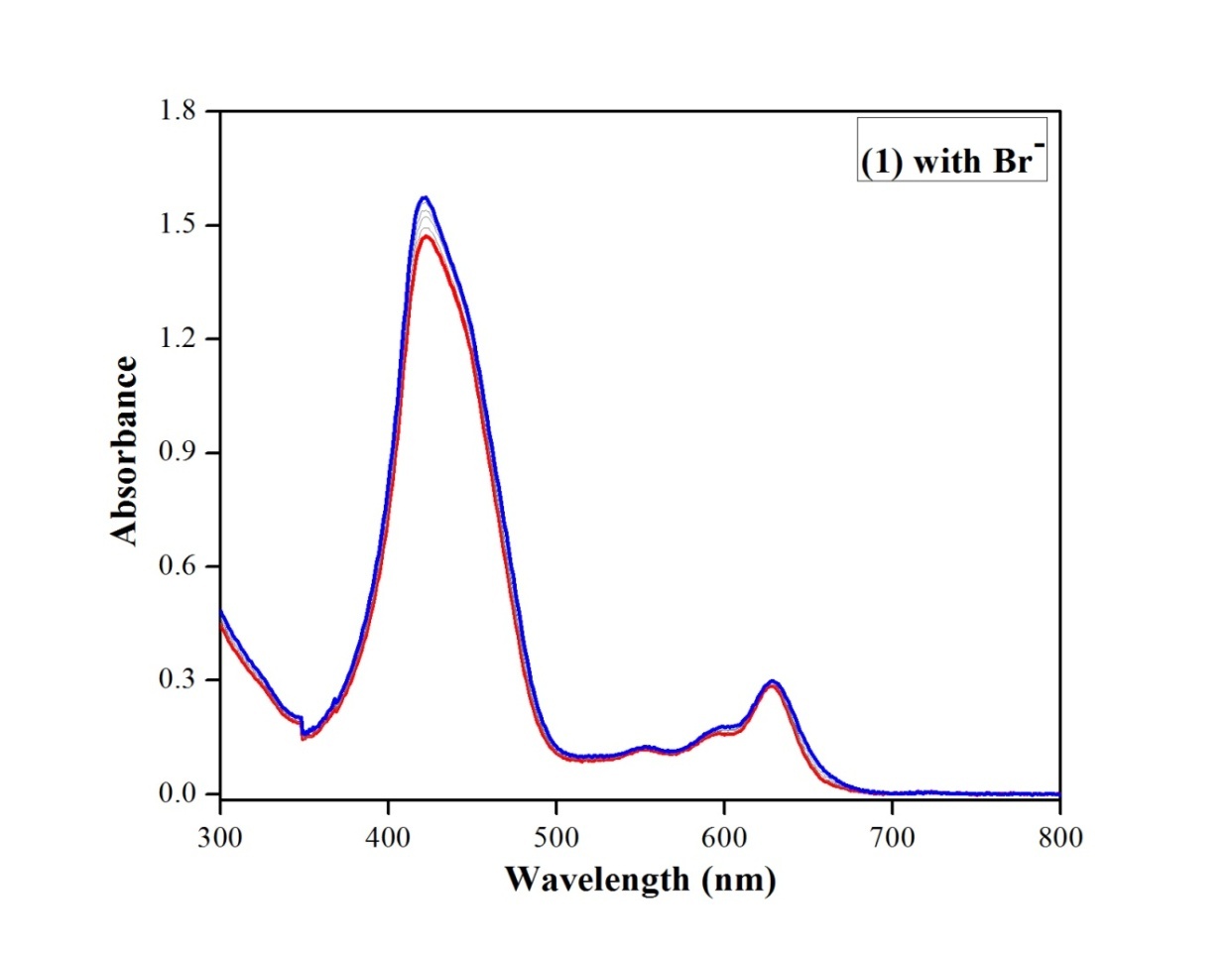


**Supplementary Figure S6:** UV-Visible spectral titration of fused –NH porphyrin (**1**) with Br^‒^ in CH_2_Cl_2_ at 298 K (adding 0-1.11×10 ̶ 3 M, 50 equiv. Br^‒^).


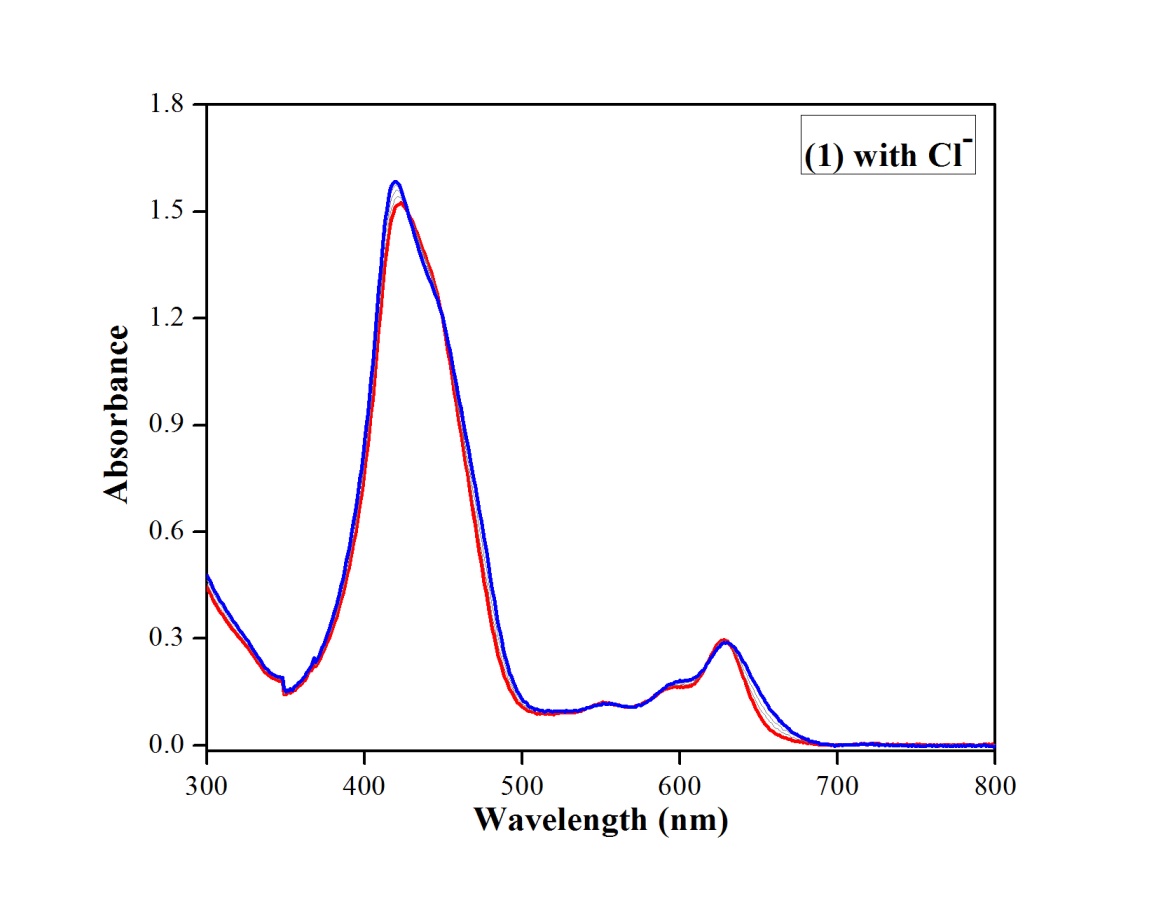


**Supplementary Figure S7:** UV-Visible spectral titration of fused –NH porphyrin (**1**) with Cl^‒^ in CH_2_Cl_2_ at 298 K (adding 0-1.11×10 ̶ 3 M, 50 equiv. Cl^‒^).


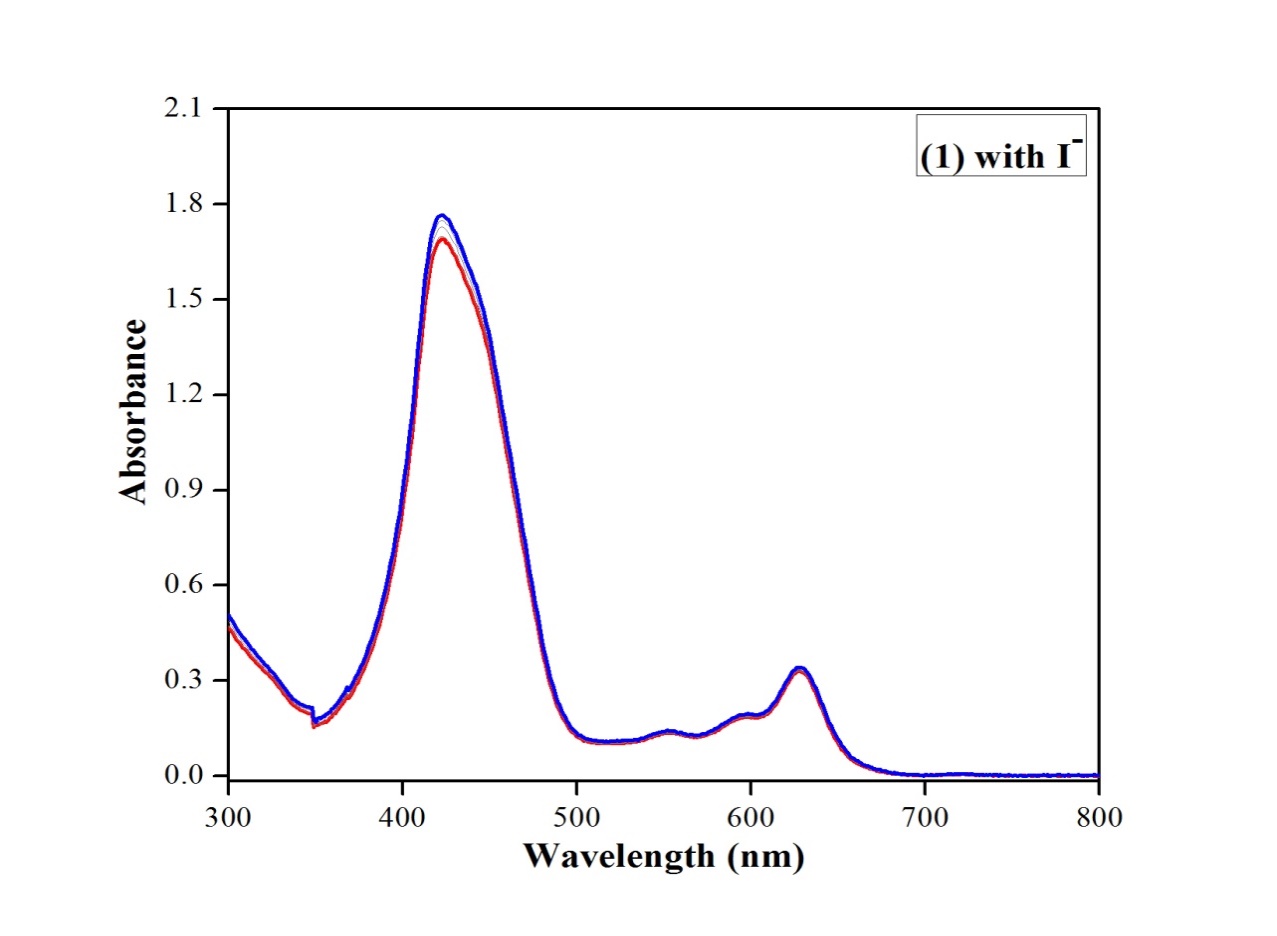


**Supplementary Figure S8:** UV-Visible spectral titration of fused –NH porphyrin (**1**) with I^‒^ in CH_2_Cl_2_ at 298 K (adding 0-1.11×10 ̶ 3 M, 50 equiv. I^‒^).


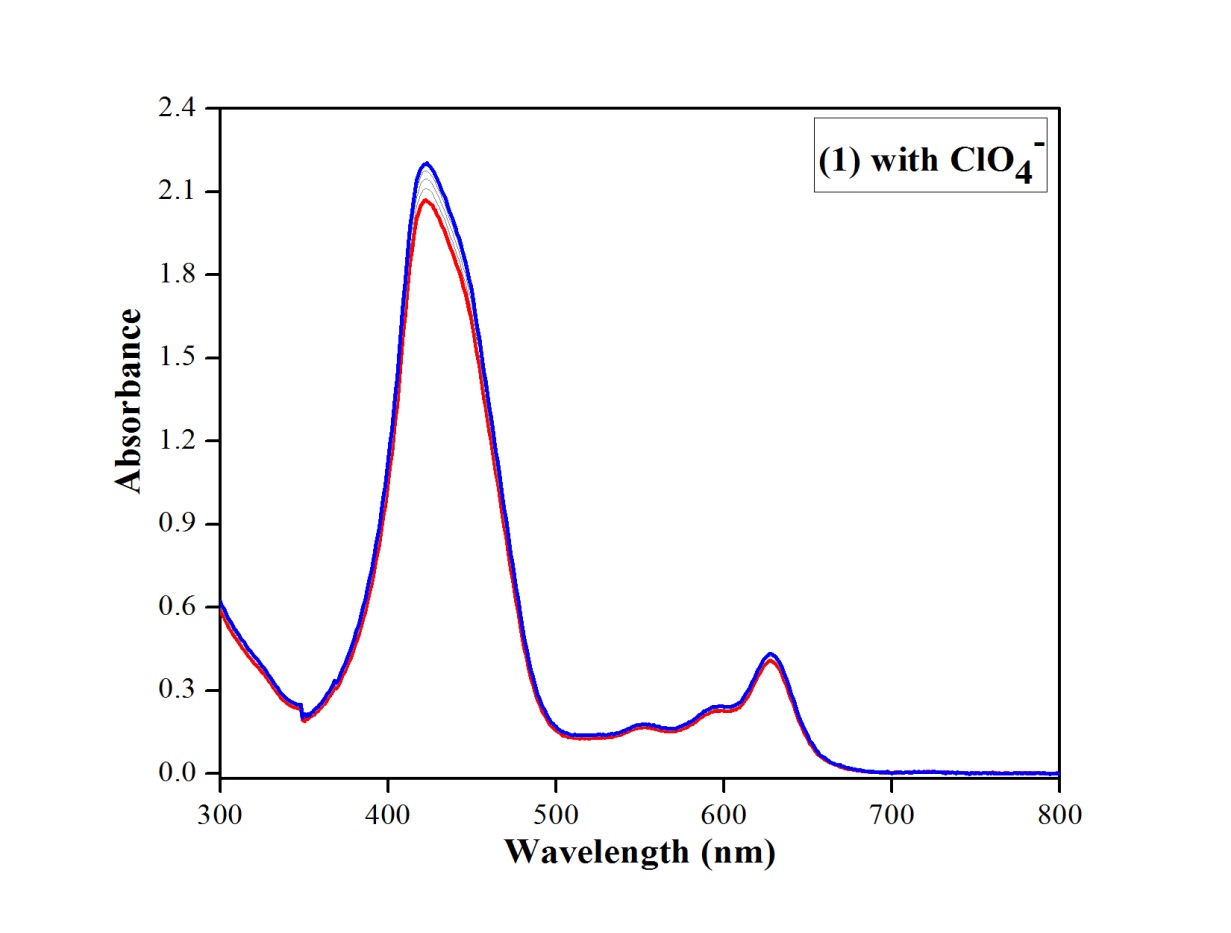


**Supplementary Figure S9:** UV-Visible spectral titration of fused –NH porphyrin (**1**) with ClO_4_^‒^ in CH_2_Cl_2_ at 298 K (adding 0-1.11×10 ̶ 3 M, 50 equiv. ClO_4_^‒^).


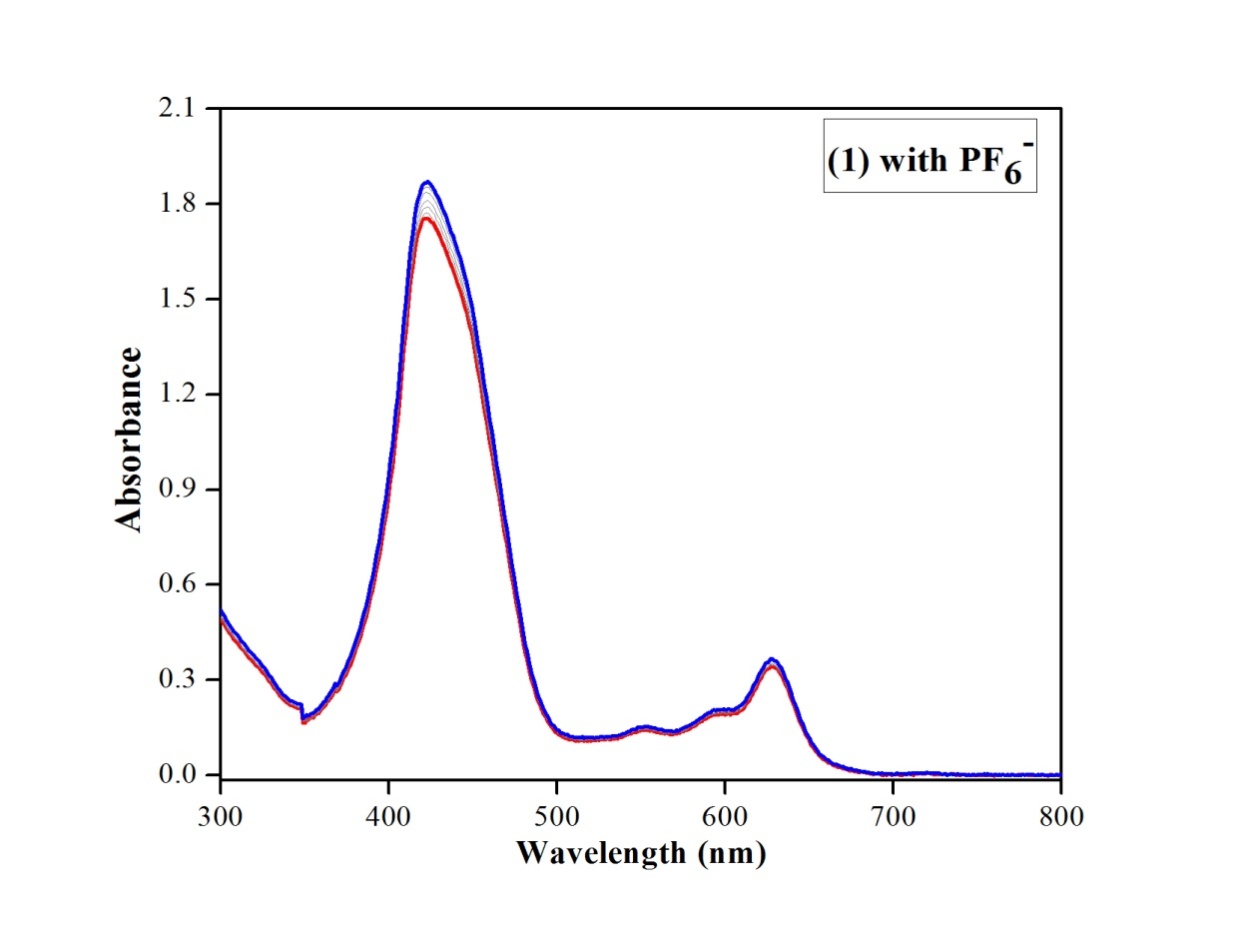


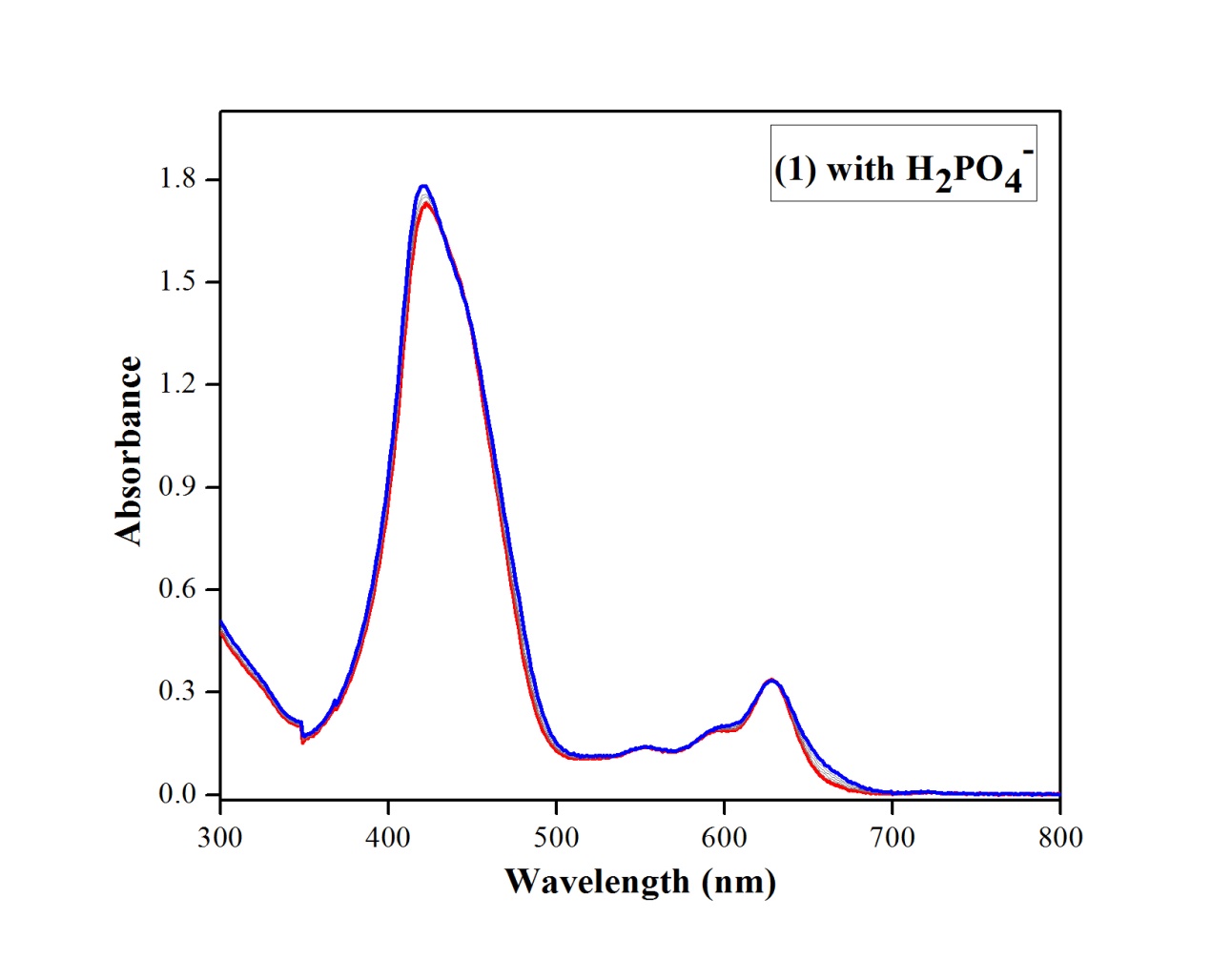
**Supplementary Figure S10:** UV-Visible spectral titration of fused –NH porphyrin (**1**) with PF_6_^‒^ in CH_2_Cl_2_ at 298 K (adding 0-1.11×10 ̶ 3 M, 50 equiv. PF_6_^‒^).

**Supplementary Figure S11:** UV-Visible spectral titration of fused –NH porphyrin (**1**) with H_2_PO_4_^‒^ in CH_2_Cl_2_ at 298 K (adding 0-1.11×10 ̶ 3 M, 50 equiv. H_2_PO_4_^‒^).


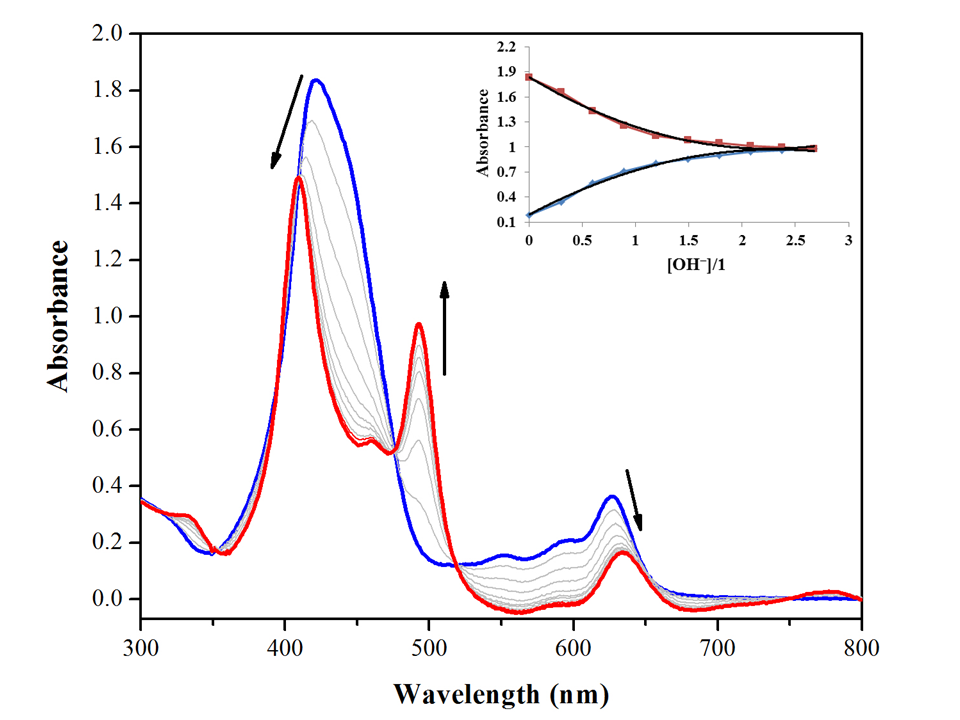


**Supplementary Figure S12:** UV-Visible spectral titration of **1** on adding 0-7.80×10^‒5^ M, 3.5 equiv. equiv. OH^−^ ions in distilled CH_2_Cl_2_ at 298 K. Inset shows decrease in absorbance at 424 nm and the concomitant increase in absorbance at 493 nm against [OH^−^]/[**1** conc.]


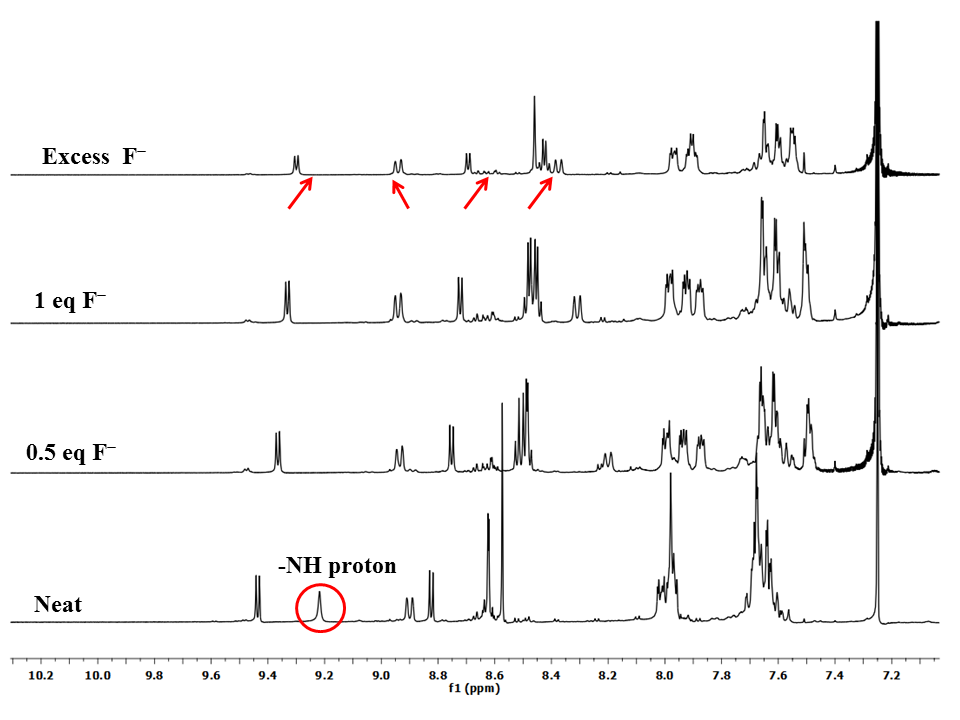


**Supplementary Figure S13:**^1^H NMR titration of **1** with F^‒^ ions in CDCl_3_ at 298 K.


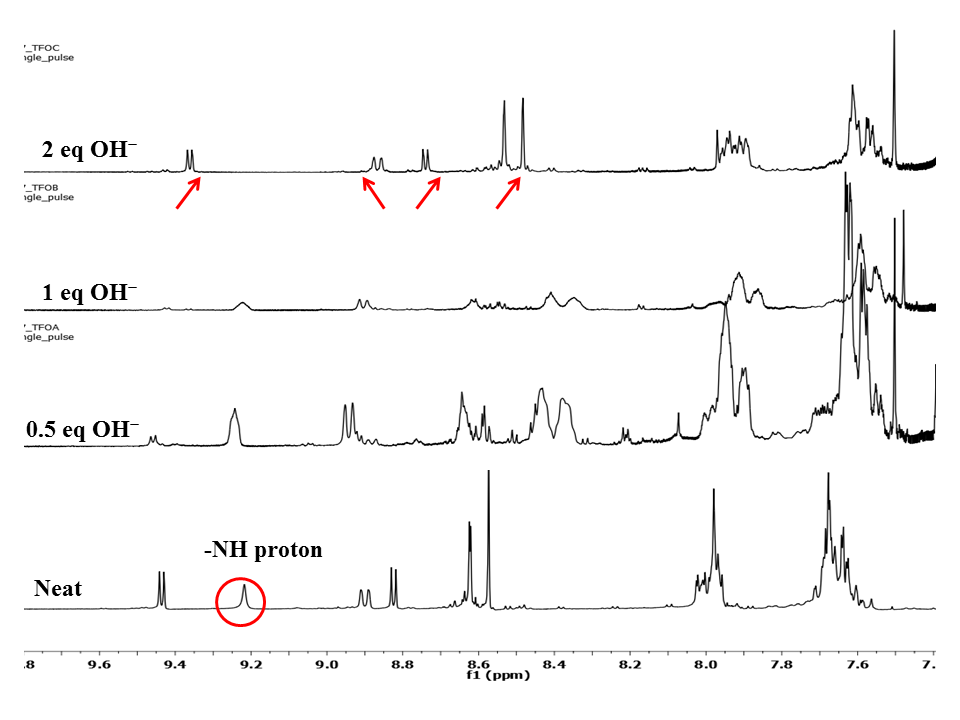


**Supplementary Figure S14:**^1^H NMR titration of **1** with OH^‒^ ions in CDCl_3_ at 298 K.


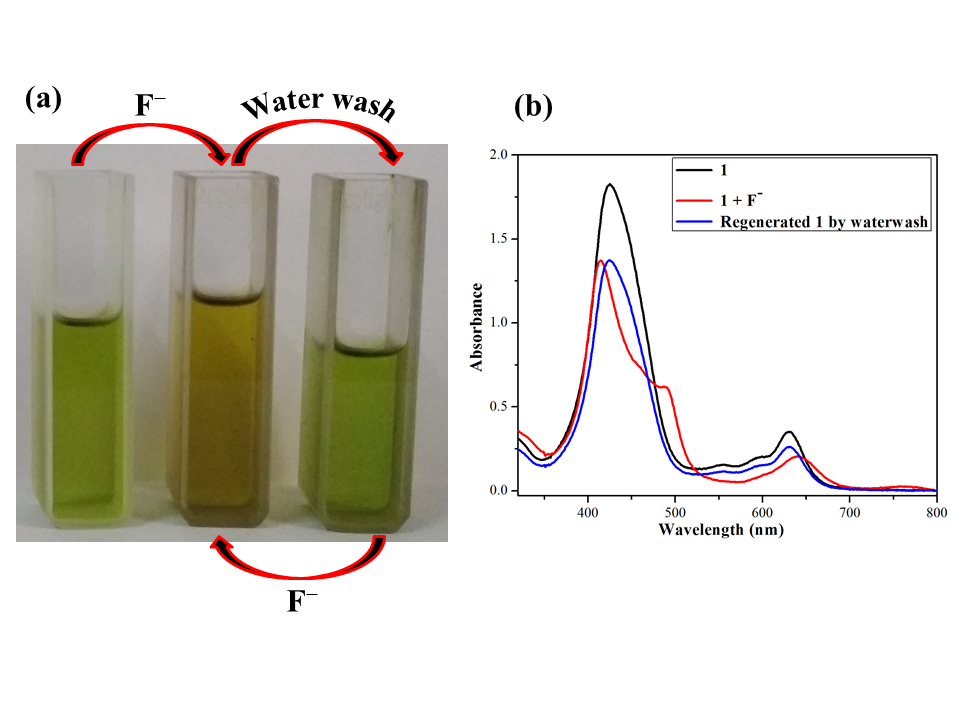


**Supplementary Figure S15:** Reversible nature of **1** after adding F^‒^ ions and water washing in terms of (a) color changes (b) UV-Visible spectral changes using CH_2_Cl_2_ and distilled water at 298 K.


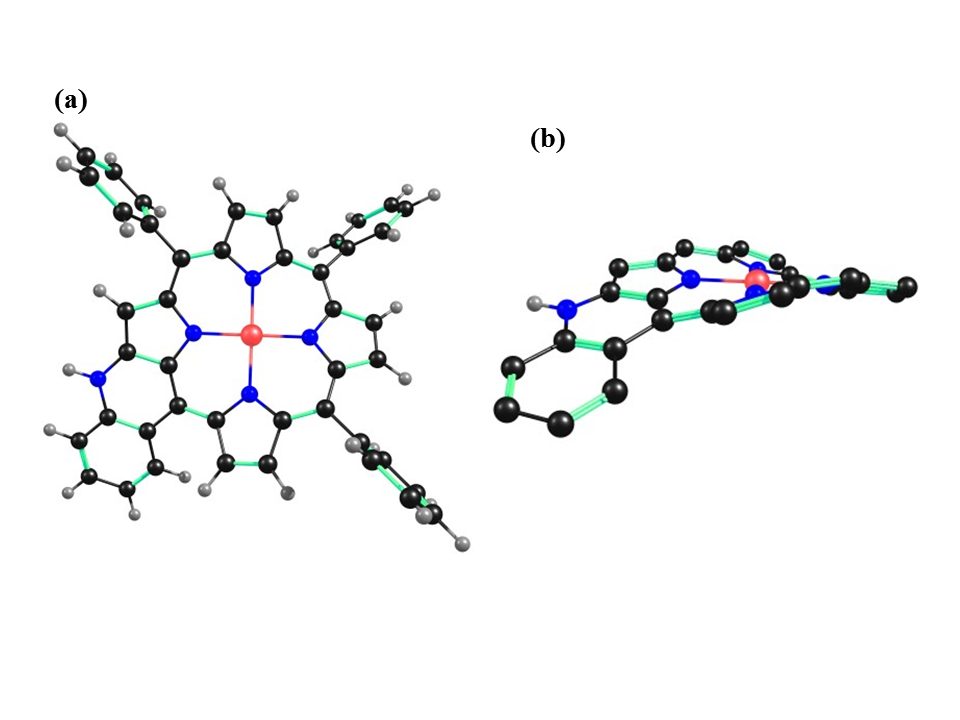


**Supplementary Figure S16:** B3LYP/LANL2DZ set optimized geometry of **1** showing (a) top view and (b) side view. In the side view all the hydrogen atoms (except fused -NH) and phenyl substituents (except cyclized phenyl) have been removed for clarity.


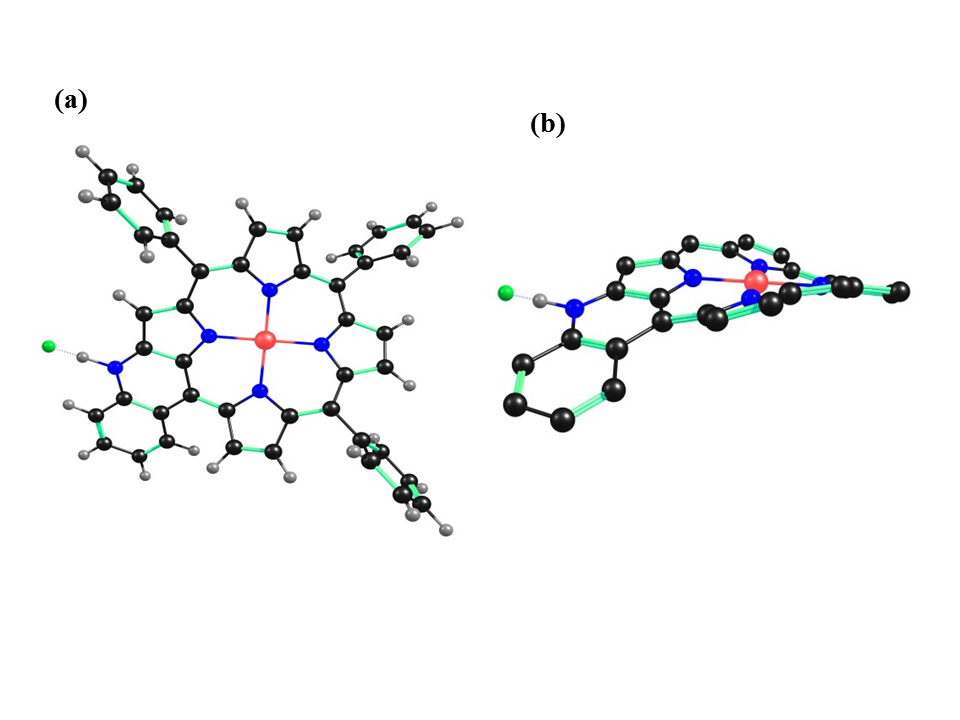


**Supplementary Figure S17:** B3LYP/LANL2DZ set optimized geometry of 1•F^−^ showing (a) top view and (b) side view. In the side view all the hydrogen atoms (except fused -NH) and phenyl substituents (except cyclized phenyl) have been removed for clarity.


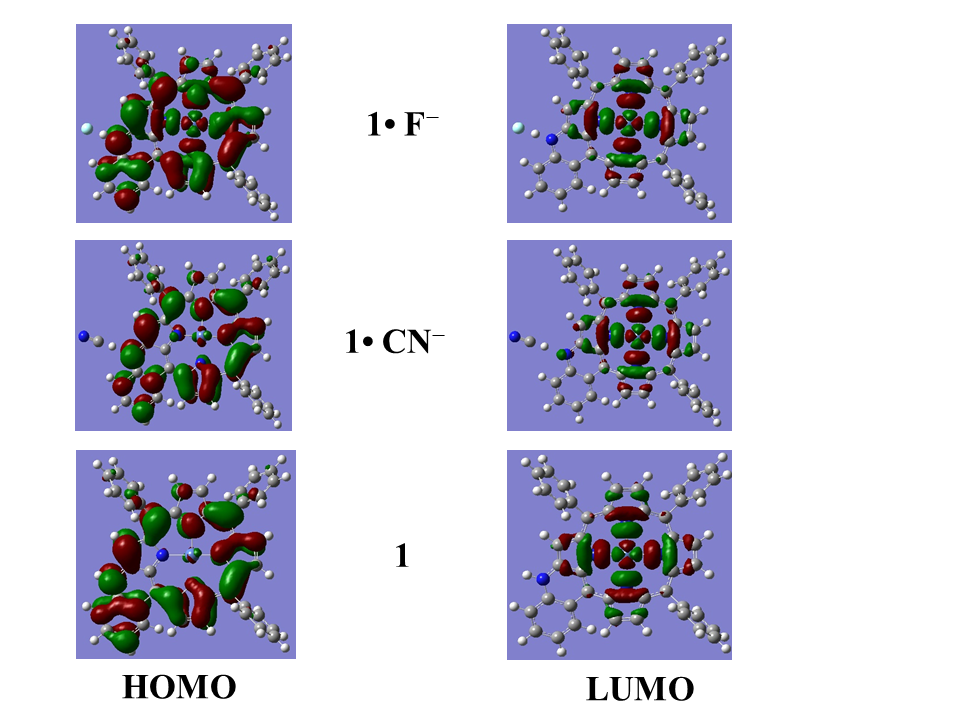


**Supplementary Figure S18:** DFT optimized geometries showing HOMO and LUMO orbitals in **1, 1**•CN^−^ and **1**•F­^−^


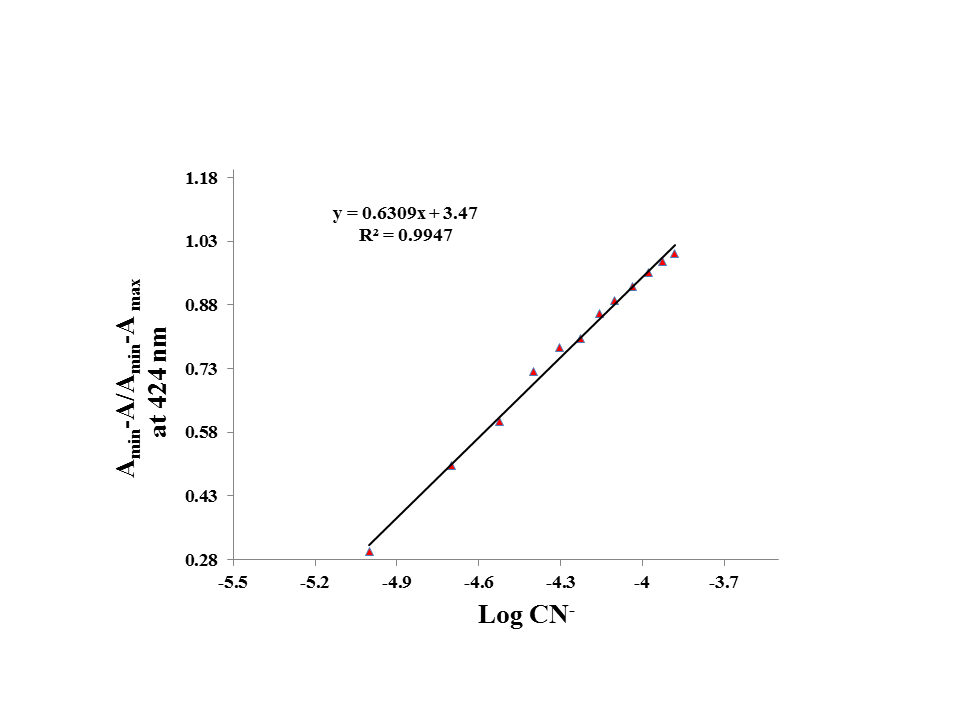


**Supplementary Figure S19:** Absorbance of **1** in CH_2_Cl_2_ normalized between maximum absorbance value at zero CN^−^ conc. and minimum absorbance value at 7 equiv. (1.56×10**^‒^**^4^ M) of CN^−^ions (for calculating detection limit).


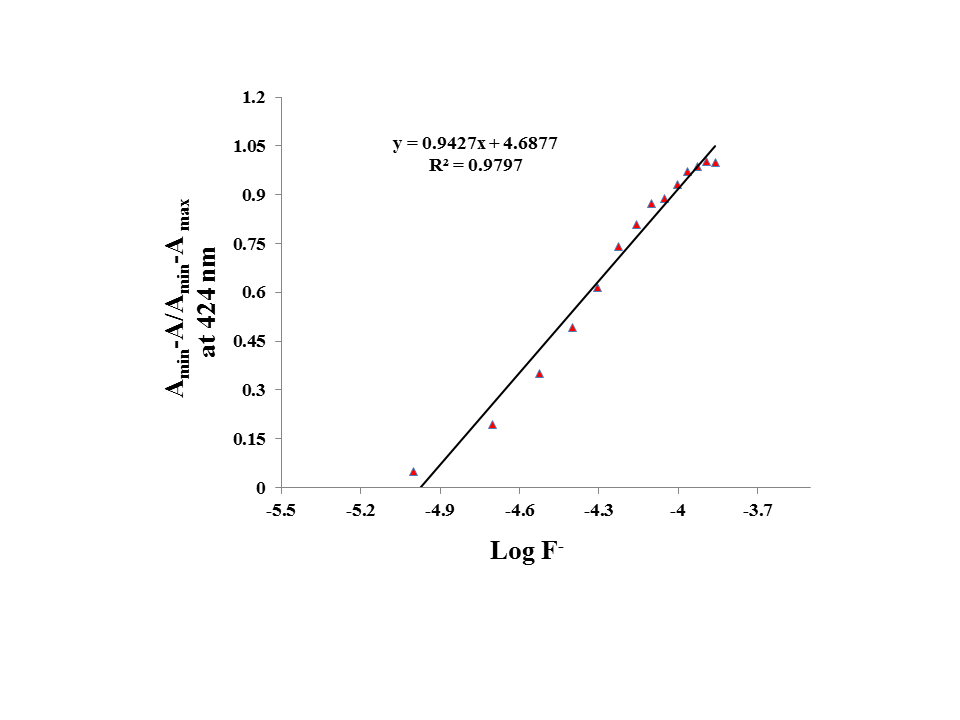


**Supplementary Figure S20:** Absorbance of **1** in CH_2_Cl_2_ normalized between maximum absorbance value at zero F^−^ conc. and minimum absorbance value at 7 equiv. (1.56×10**^‒^**^4^ M) of F^−^ ions (for calculating detection limit).


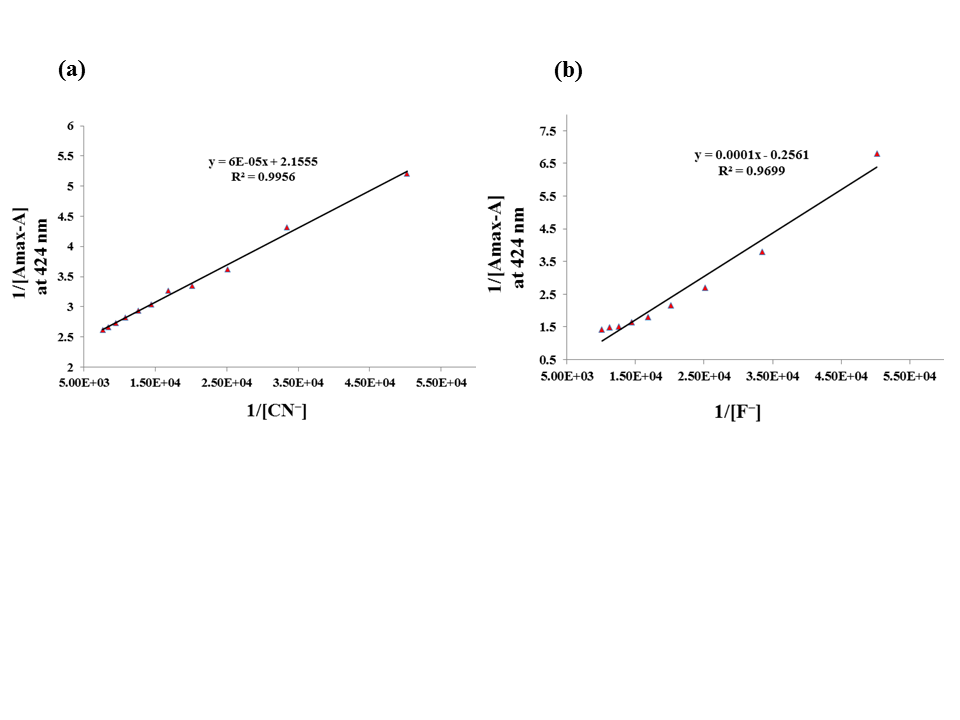


**Supplementary Figure S21:** BH Plots obtained from UV-Visible spectral titration of **1** with (a) CN^−^ and (b) F^−^ ions in distilled CH_2_Cl_2_ at 298 K indicating 1:1 stoichiometry between **1** and the added anions.


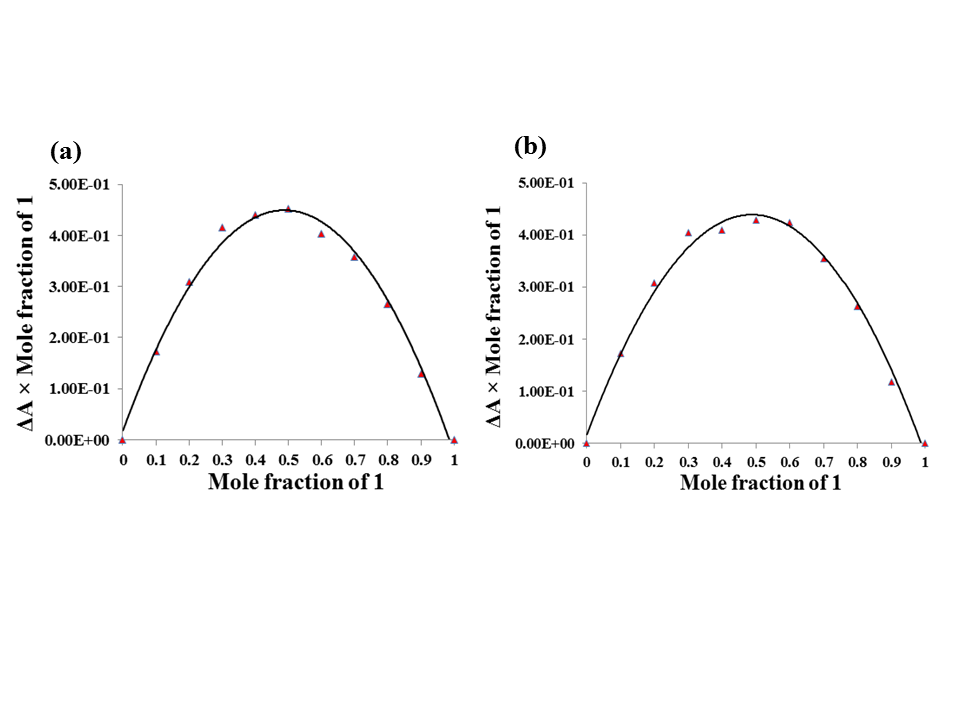


**Supplementary Figure S22:** Job’s plot for (a) CN^−^─ **1** (b) F^−^─ **1** interactions in distilled CH_2_Cl_2_ at 298 K indicating 1:1 stoichiometry. Total concentration of ([anion] + [**1**]) = 2.23×10^–5^ M.


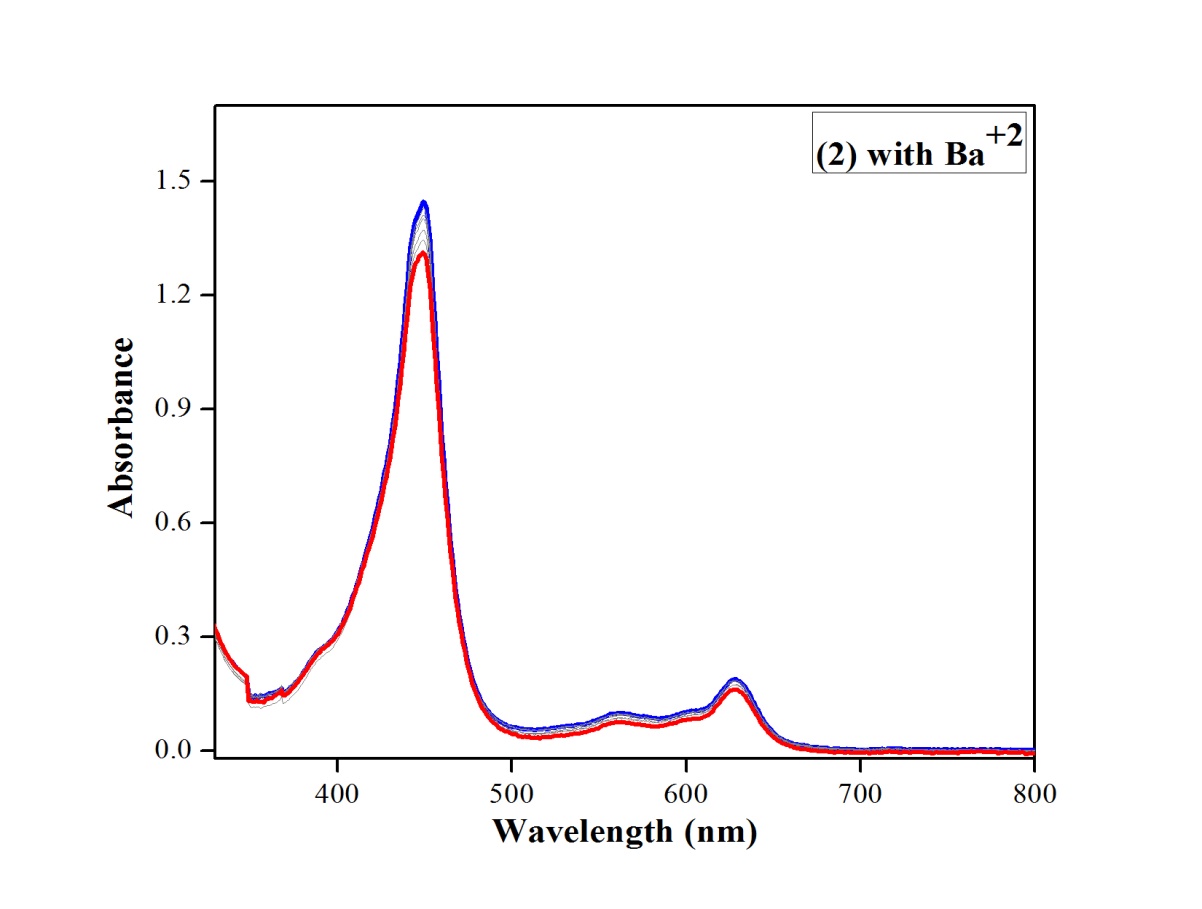


**Supplementary Figure S23:** UV-Visible spectral titration of fused –CHO porphyrin (**2**) with Ba^+2^ in CHCl_3_ at 298 K (adding 0-1.3×10 ̶ 4 M, 10 equiv. Ba^+2^).


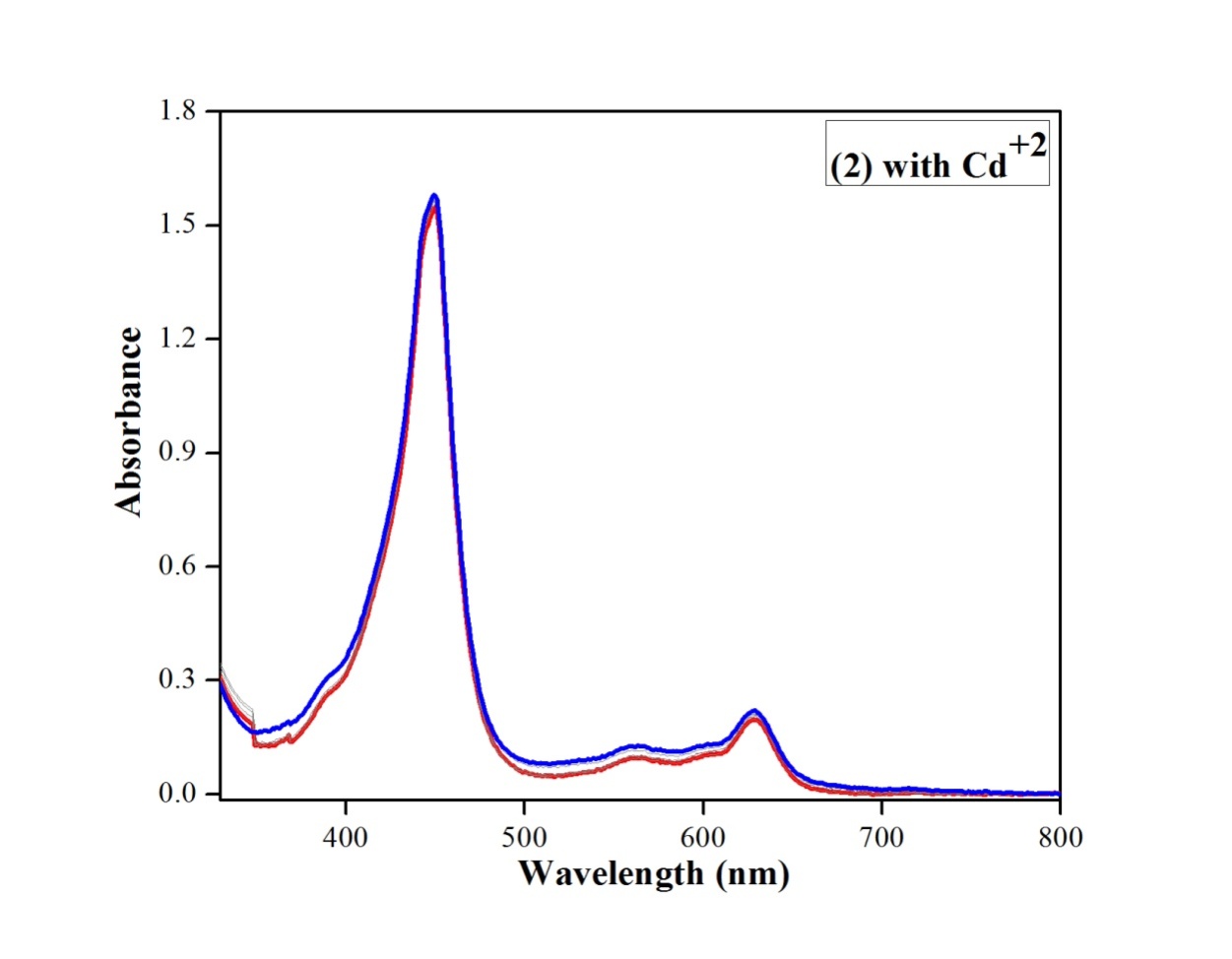


**Supplementary Figure S24:** UV-Visible spectral titration of fused –CHO porphyrin (**2**) with Cd^+2^ in CHCl_3_ at 298 K (adding 0-1.3×10 ̶ 4 M, 10 equiv. Cd^+2^).


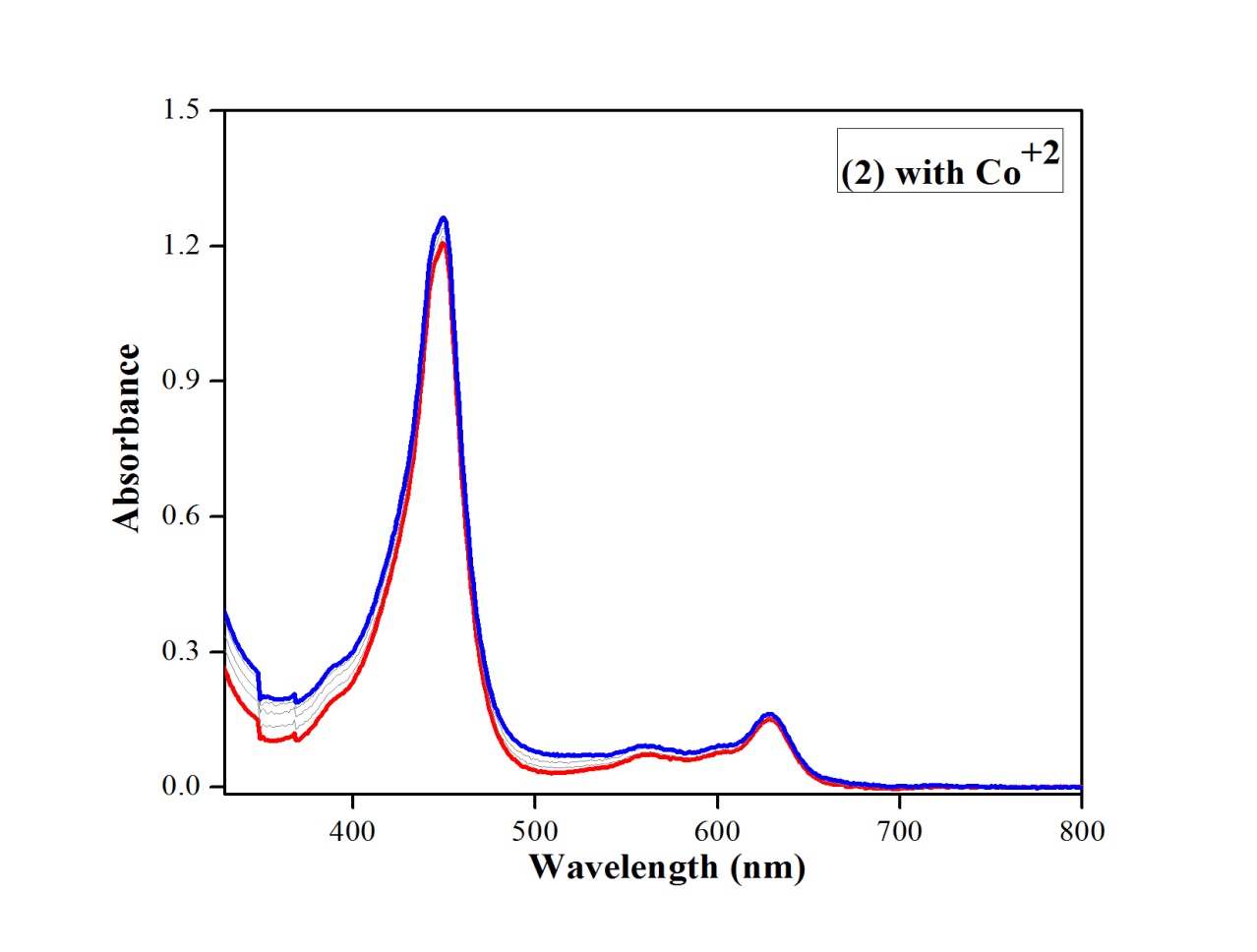


**Supplementary Figure S25:** UV-Visible spectral titration of fused –CHO porphyrin (**2**) with Co^+2^ in CHCl_3_ at 298 K (adding 0-1.3×10 ̶ 4 M, 10 equiv. Co^+2^).


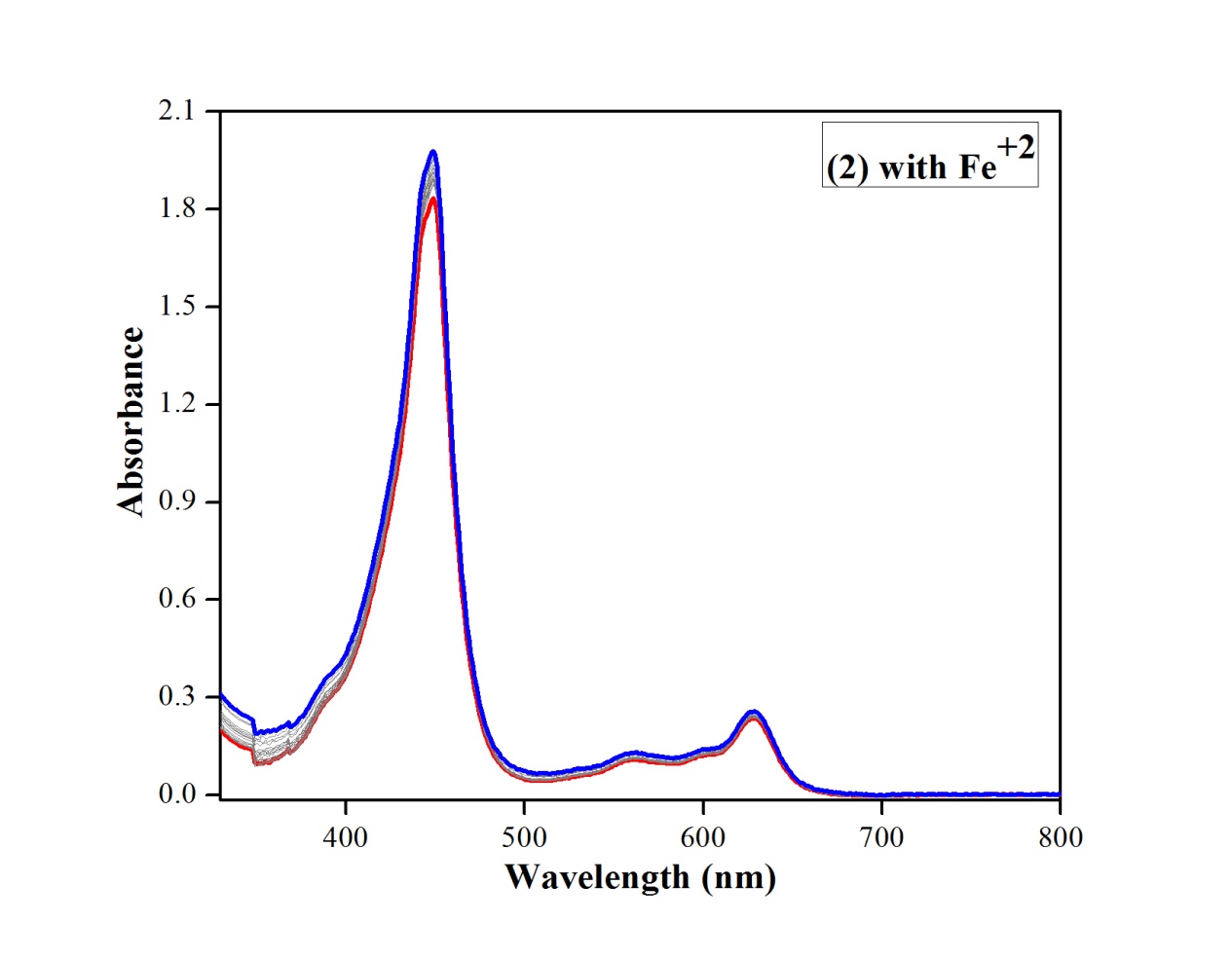


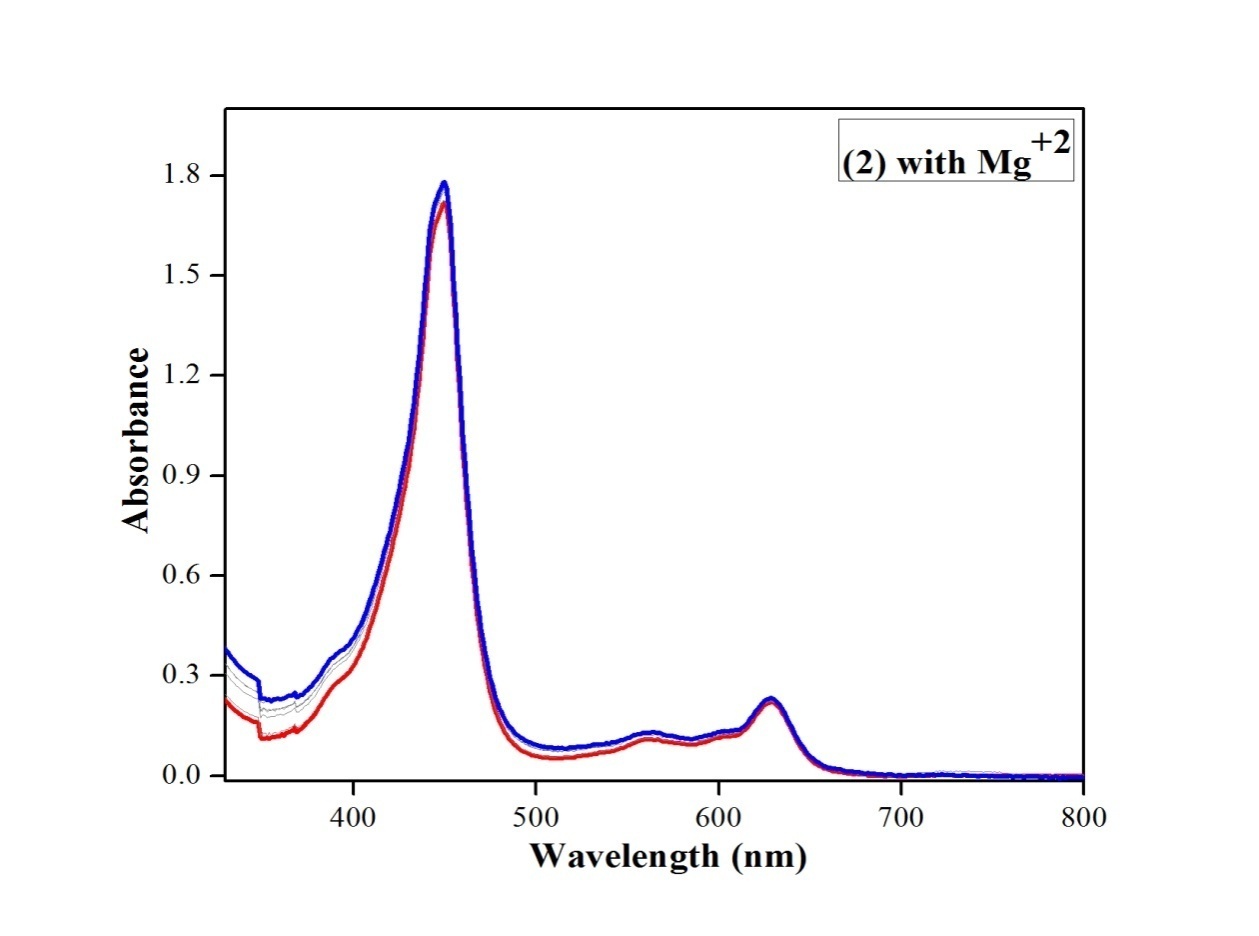
**Supplementary Figure S26:** UV-Visible spectral titration of fused –CHO porphyrin (**2**) with Fe^+2^ in CHCl_3_ at 298 K (adding 0-1.3×10 ̶ 4 M, 10 equiv. Fe^+2^).

**Supplementary Figure S27:** UV-Visible spectral titration of fused –CHO porphyrin (**2**) with Mg^+2^ in CHCl_3_ at 298 K (adding 0-1.3×10 ̶ 4 M, 10 equiv. Mg^+2^).


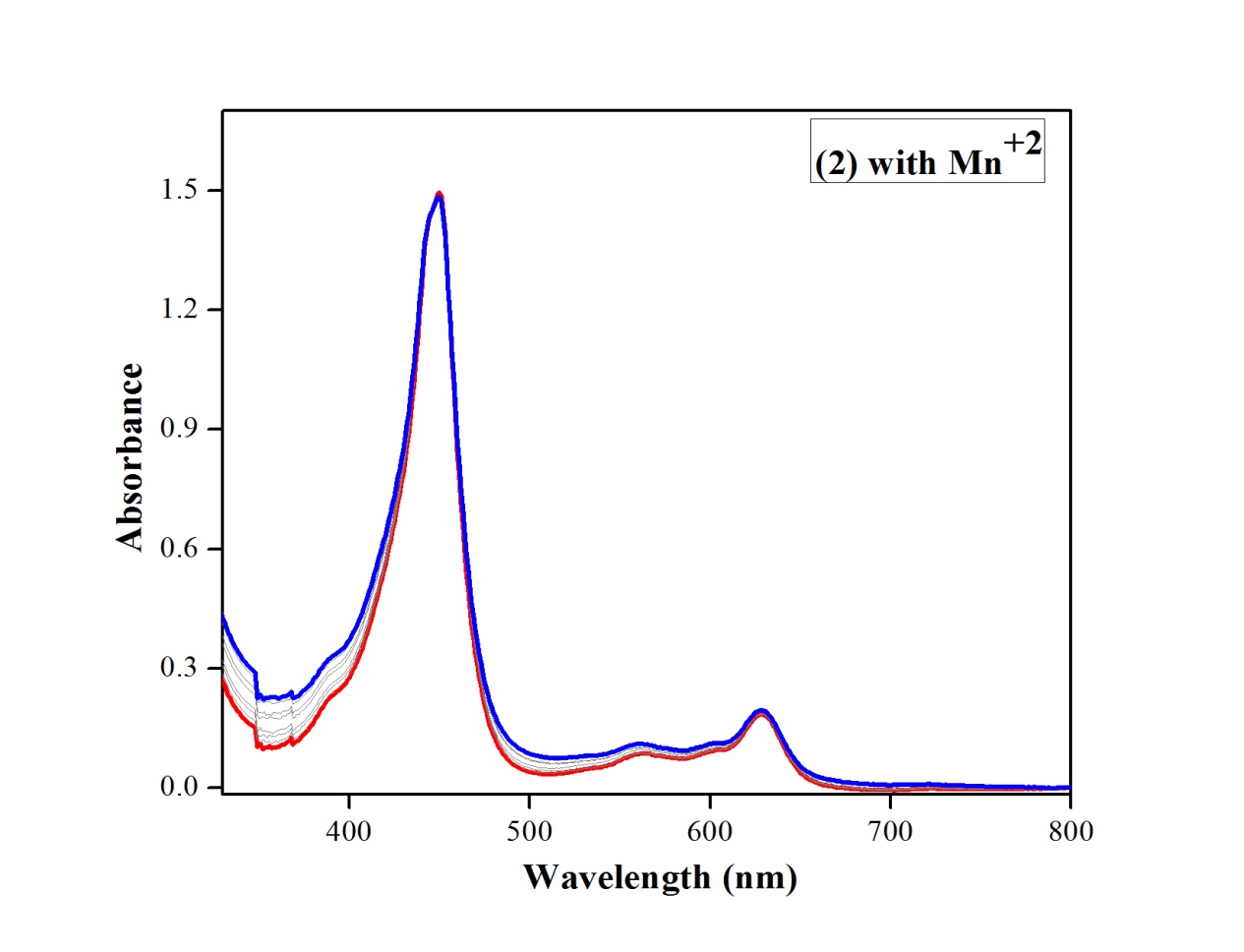


**Supplementary Figure S28:** UV-Visible spectral titration of fused –CHO porphyrin (**2**) with Mn^+2^ in CHCl_3_ at 298 K (adding 0-1.3×10 ̶ 4 M, 10 equiv. Mn^+2^).


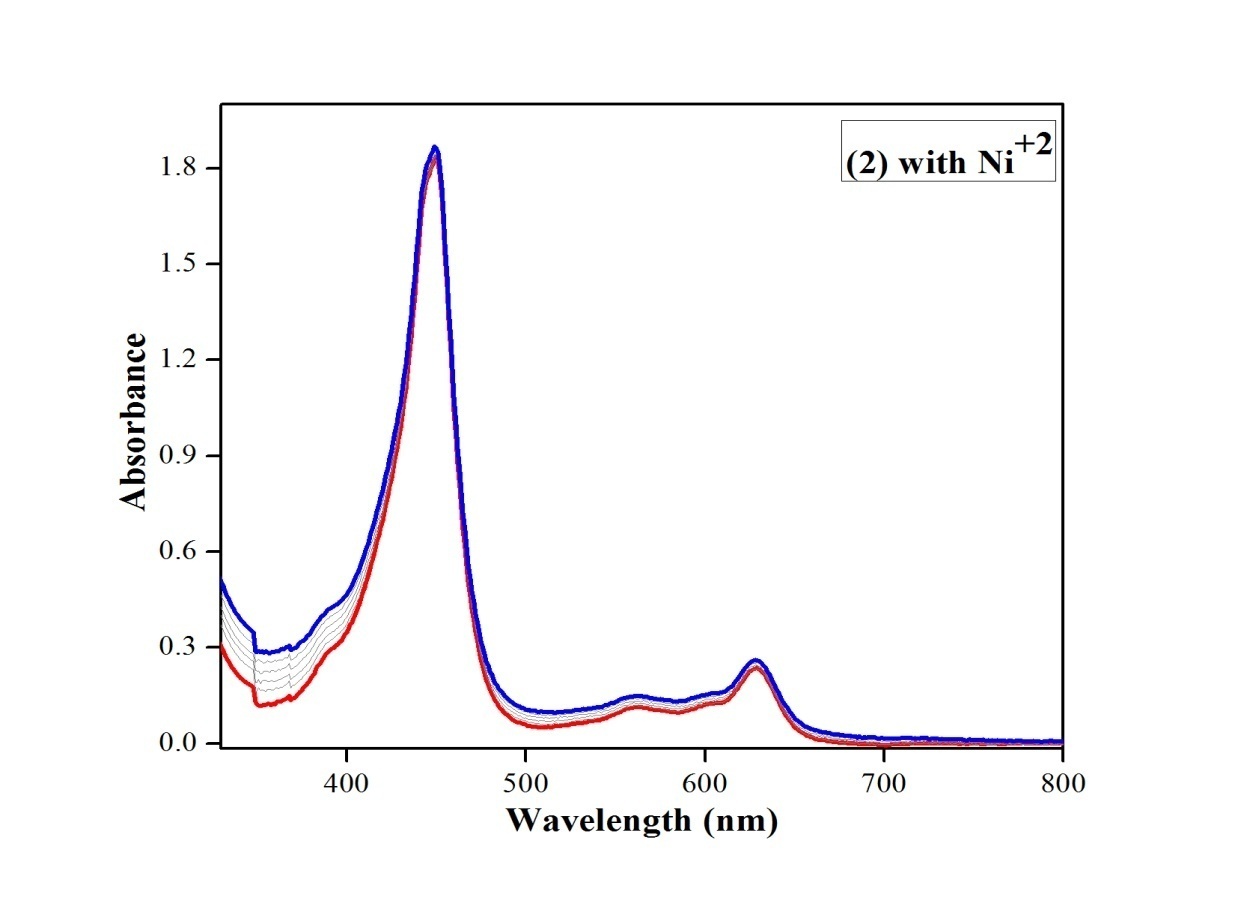


**Supplementary Figure S29:** UV-Visible spectral titration of fused –CHO porphyrin (**2**) with Ni^+2^ in CHCl_3_ at 298 K (adding 0-1.3×10 ̶ 4 M, 10 equiv. Ni^+2^).


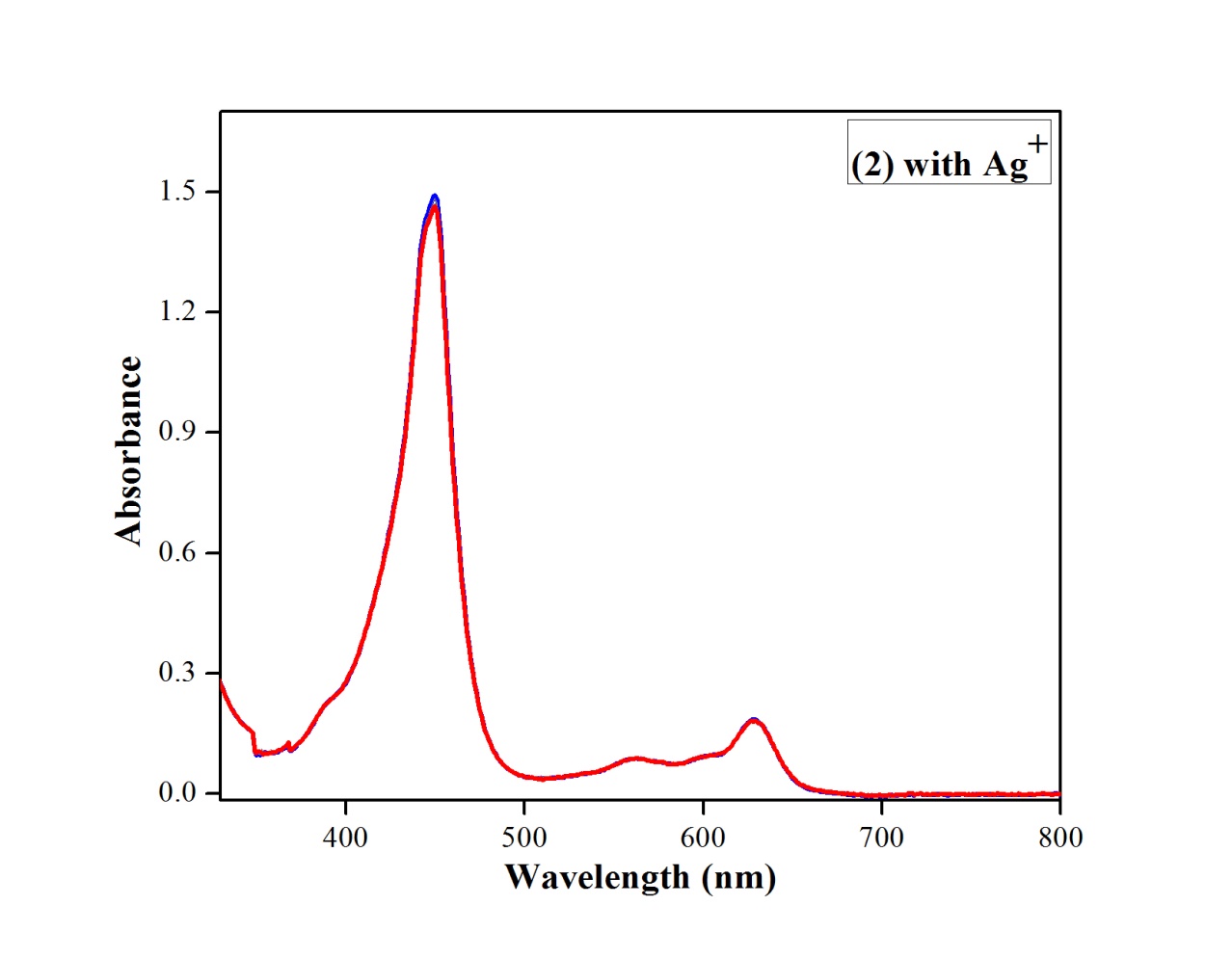


**Supplementary Figure S30:** UV-Visible spectral titration of fused –CHO porphyrin (**2**) with Ag^+^ in CHCl_3_ at 298 K (adding 0-1.3×10 ̶ 4 M, 10 equiv. Ag^+^).


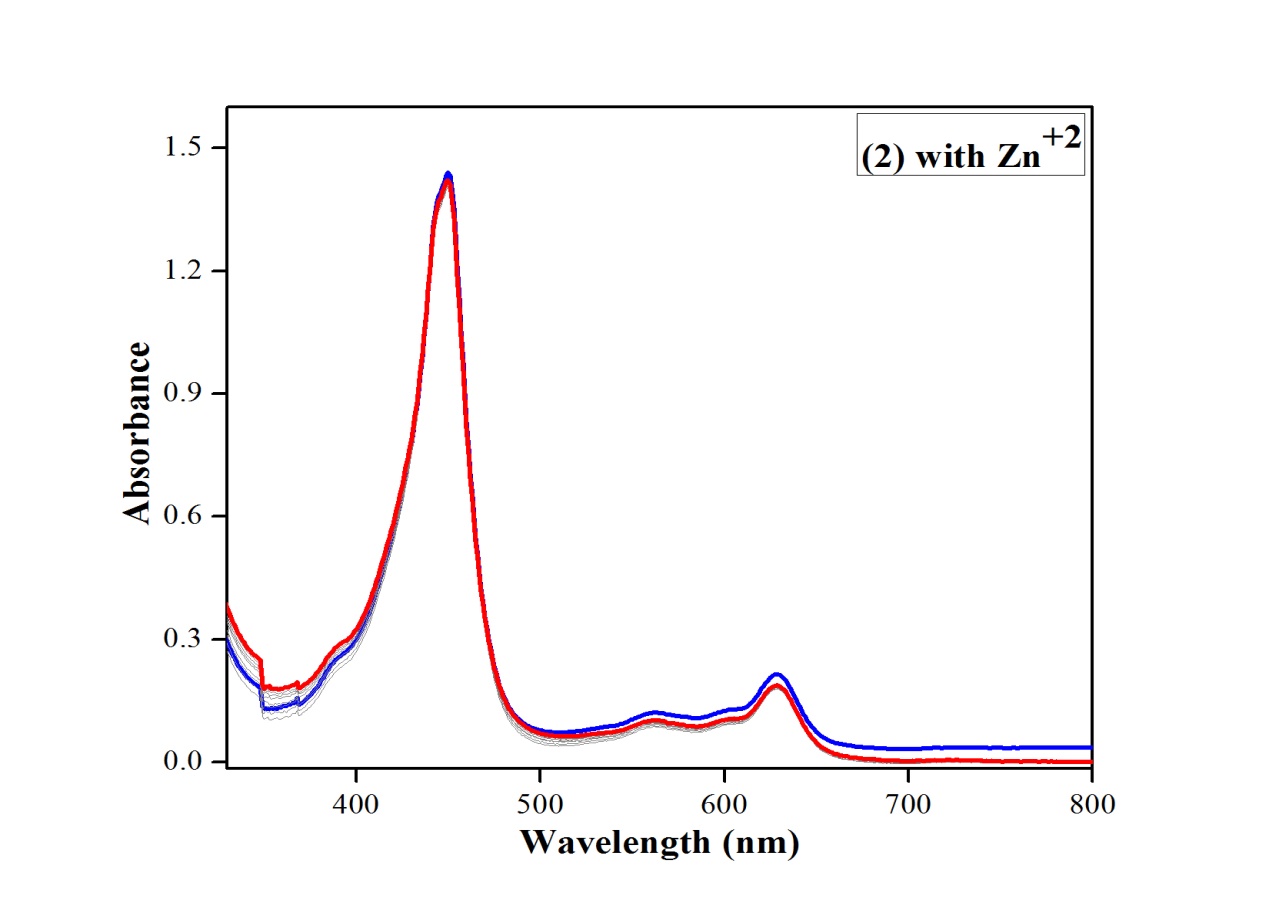


**Supplementary Figure S31:** UV-Visible spectral titration of fused –CHO porphyrin (**2**) with Zn^+2^ in CHCl_3_ at 298 K (adding 0-1.3×10 ̶ 4 M, 10 equiv. Zn^+2^).


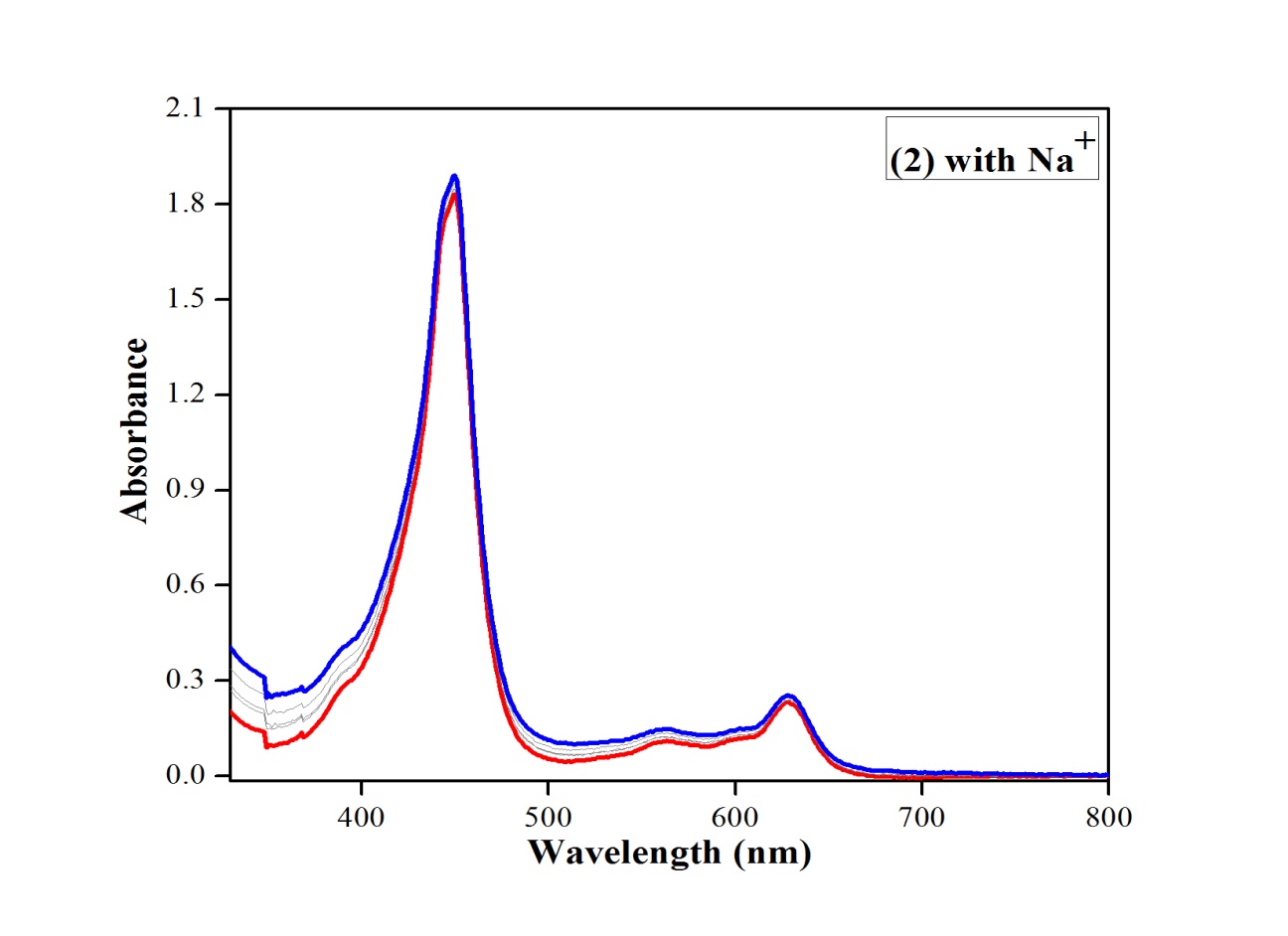


**Supplementary Figure S32:** UV-Visible spectral titration of fused –CHO porphyrin (**2**) with Na^+^ in CHCl_3_ at 298 K (adding 0-1.3×10 ̶ 4 M, 10 equiv. Na^+^).


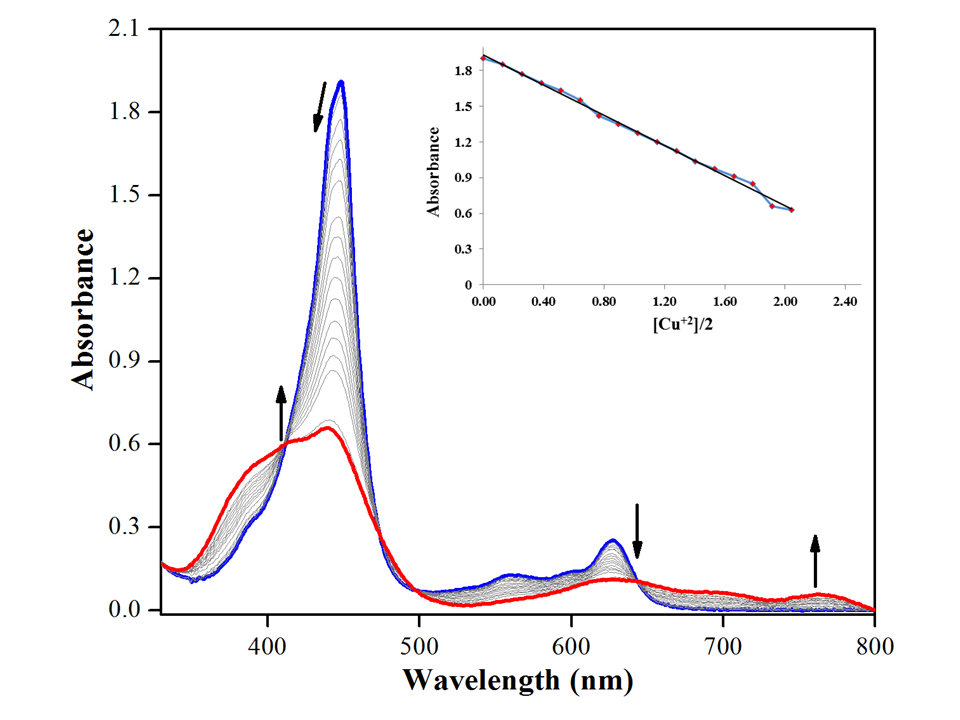


**Supplementary Figure S33:** UV-Visible spectral titration of **2** on adding (a) 0-4.55×10^-5^ M, 3.5 equiv. of Cu^+2^ ions in distilled CHCl_3_ at 298 K. Insets show decrease in absorbance at 447 nm against [cation conc.]/[**2** conc.]


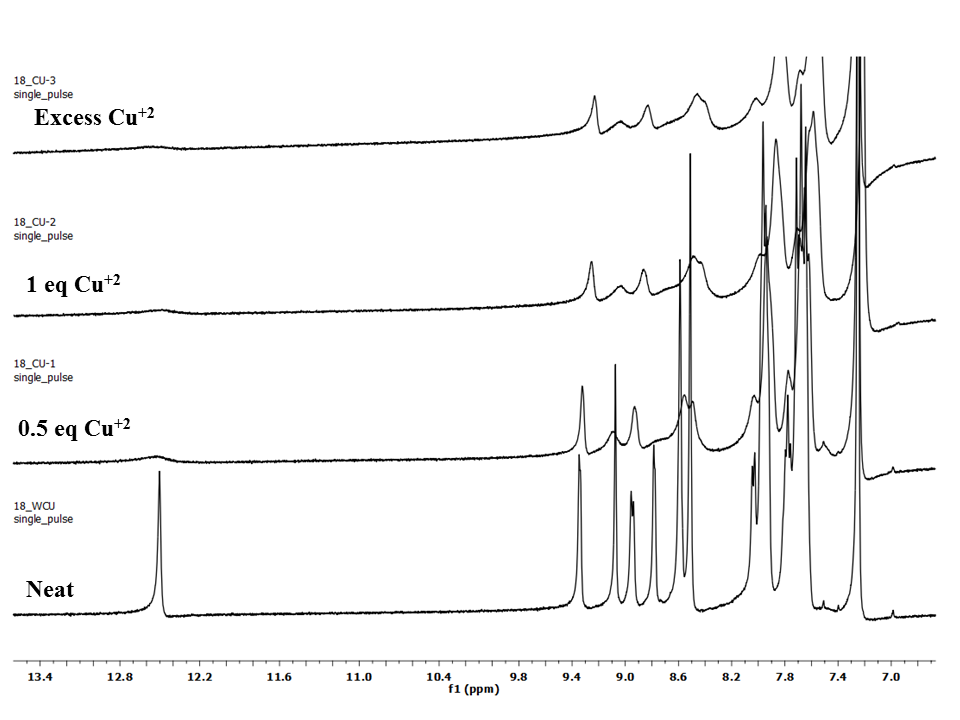


**Supplementary Figure S34:**^1^H NMR titration of **2 (**in CDCl_3_**)** with Cu^+2^ ions (in DMSO-d^6^) at 298 K.


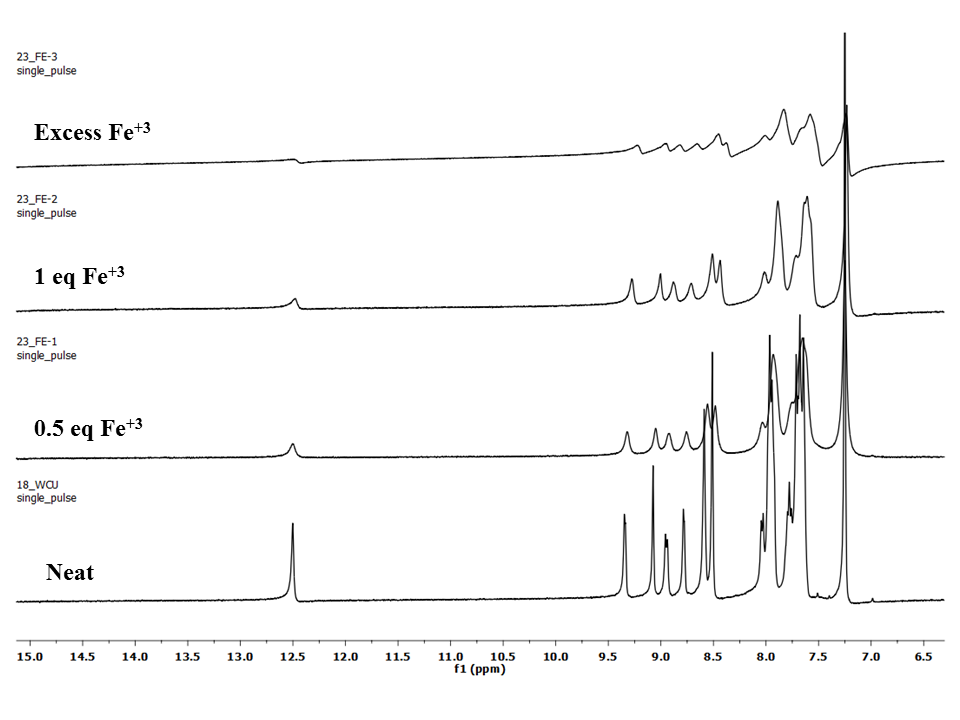


**Supplementary Figure S35:**^1^H NMR titration of **2 (**in CDCl_3_**)** with Fe^+3^ ions (in DMSO-d^6^) at 298 K.


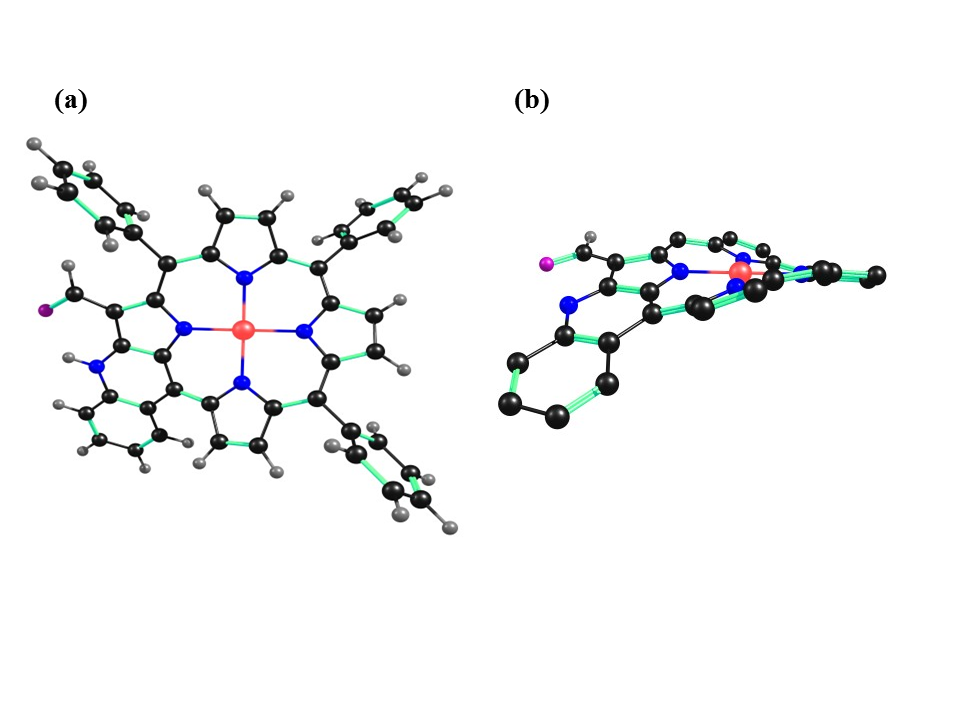


**Supplementary Figure S36:** B3LYP/LANL2DZ set optimized geometry of **2** showing (a) top view and (b) side view. In the side view all the hydrogen atoms (except -CHO) and phenyl substituents (except cyclized phenyl) have been removed for clarity.


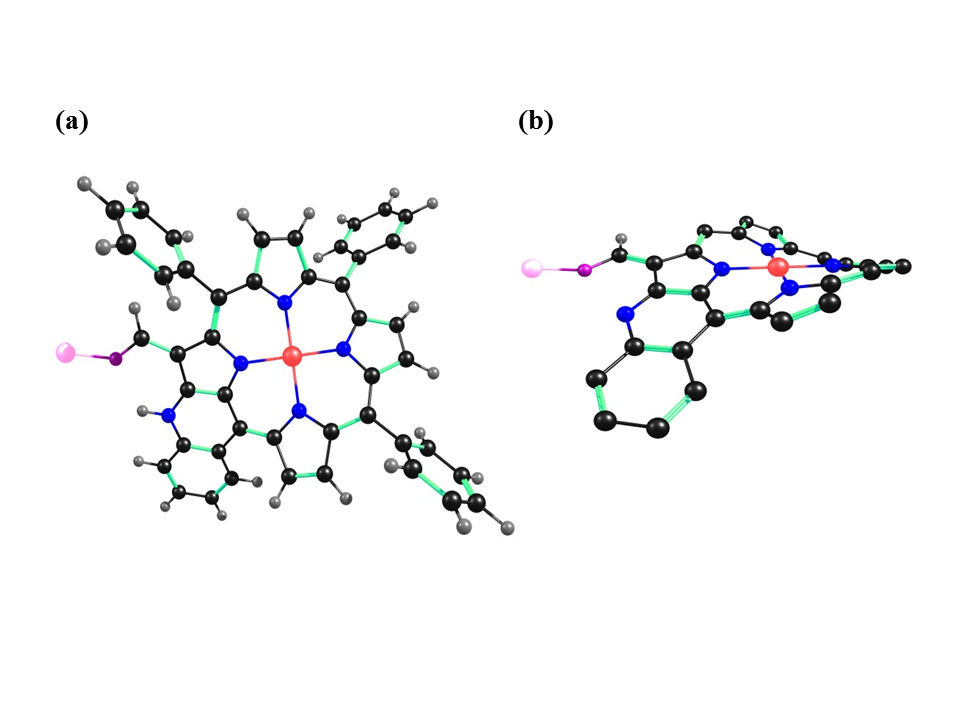


**Supplementary Figure S37:** B3LYP/LANL2DZ set optimized geometry of **2**•Fe^+3^ showing (a) top view and (b) side view. In the side view all the hydrogen atoms (except -CHO) and phenyl substituents (except cyclized phenyl) have been removed for clarity.


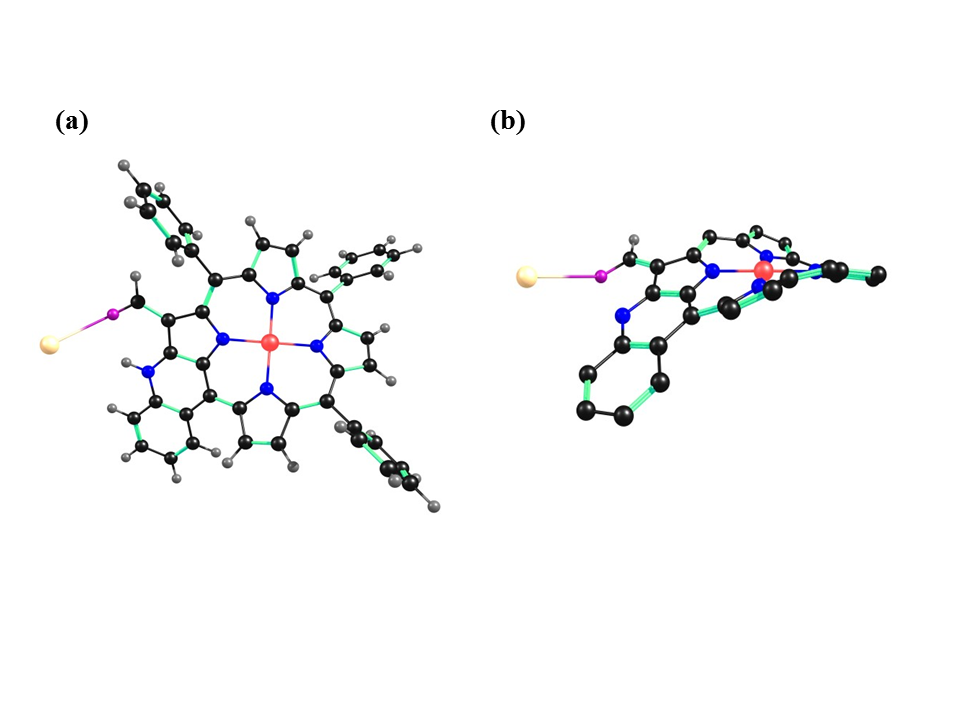


**Supplementary Figure S38:** B3LYP/LANL2DZ set optimized geometry of **2**•Hg^+2^ showing (a) top view and (b) side view. In the side view all the hydrogen atoms (except -CHO) and phenyl substituents (except cyclized phenyl) have been removed for clarity.


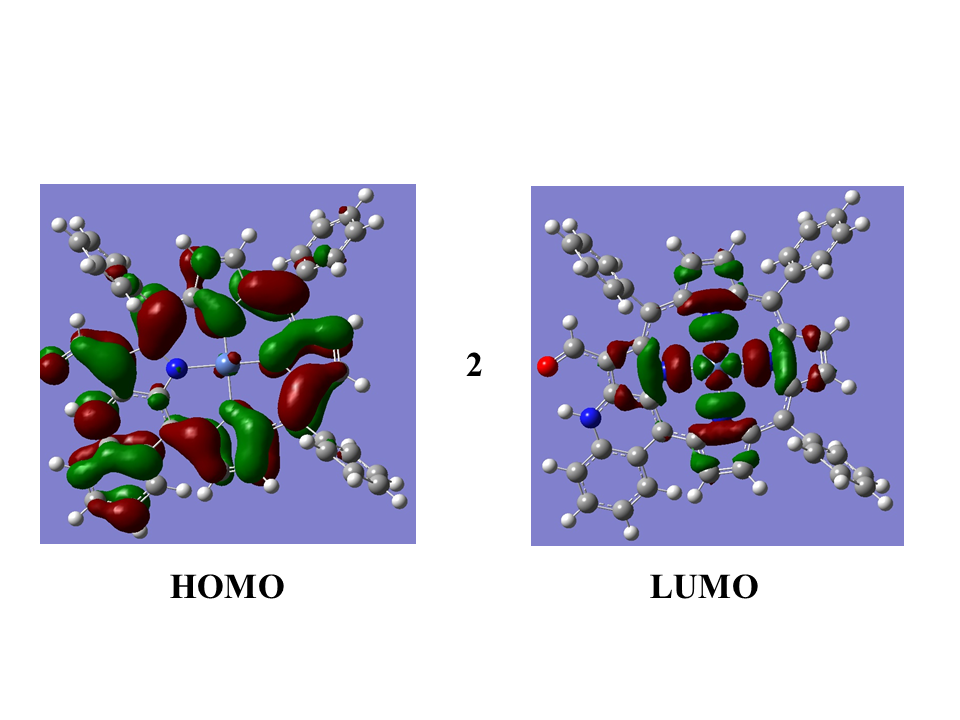


**Supplementary Figure S39:** DFT optimized geometries showing HOMO and LUMO orbitals in **2**.


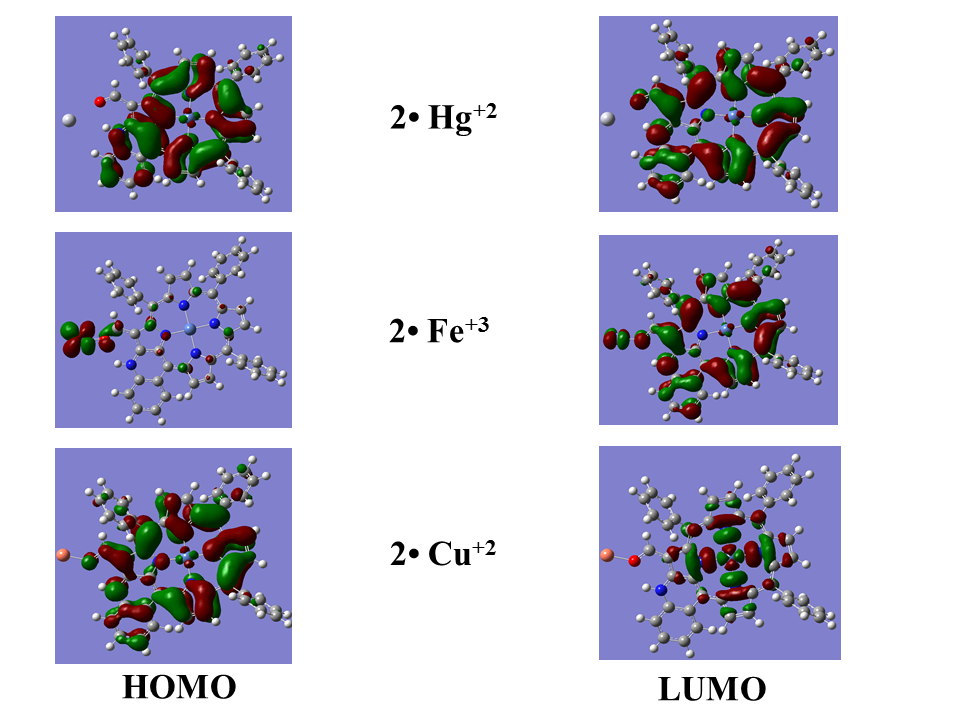


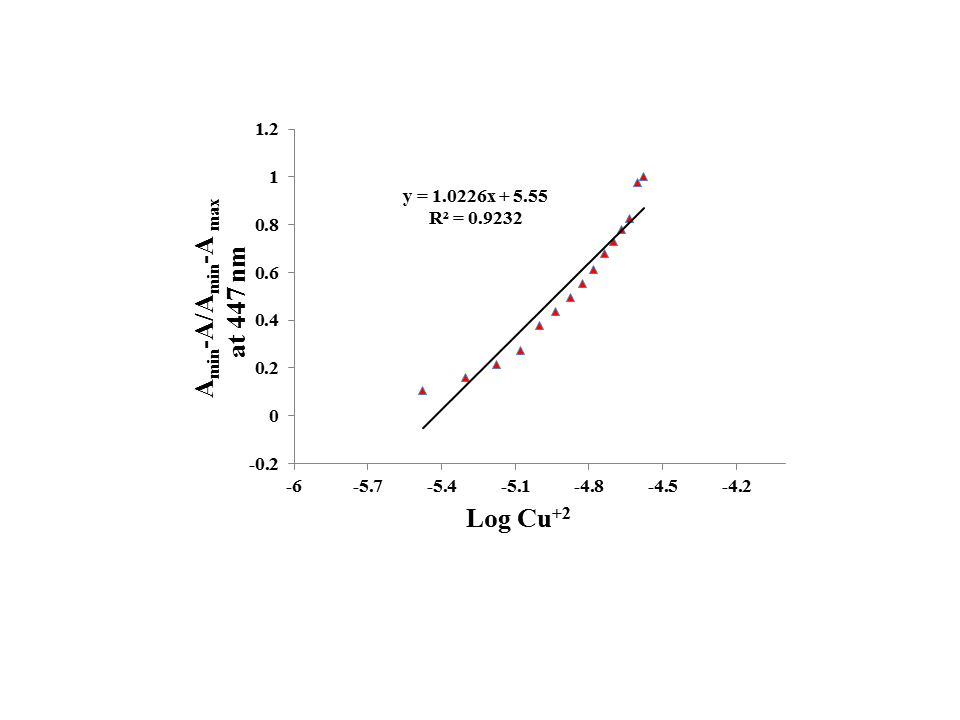
**Supplementary Figure S40:** DFT optimized geometries showing HOMO and LUMO orbitals in **2**•Cu^+2^, **2**•Fe^+3^ and **2**•Hg^+2^ respectively.

**Supplementary Figure S41:** Absorbance of **2** in CHCl_3_ normalized between maximum absorbance value at zero Cu^+2^ conc. and minimum absorbance value at 3.5 equiv. (4.55×10^-5^M) of Cu^+2^ ions (for calculating detection limit).


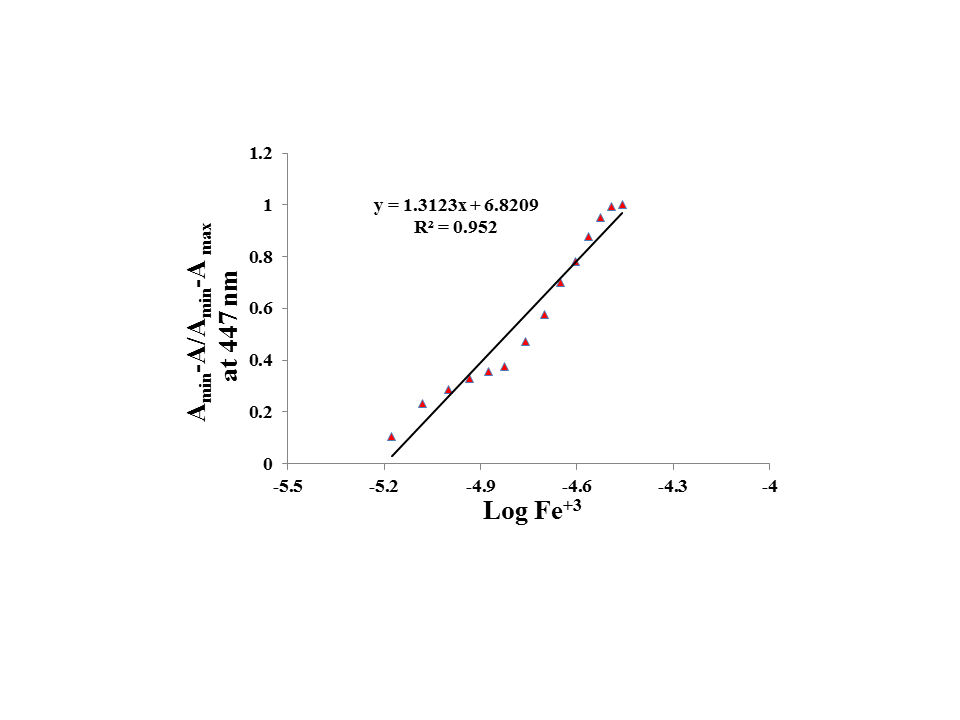


**Supplementary Figure S42:** Absorbance of **2** in CHCl_3_ normalized between maximum absorbance value at zero Fe^+3^ conc. and minimum absorbance value at 3.5 equiv. (4.55×10^-5^M) of Fe^+3^ ions (for calculating detection limit).


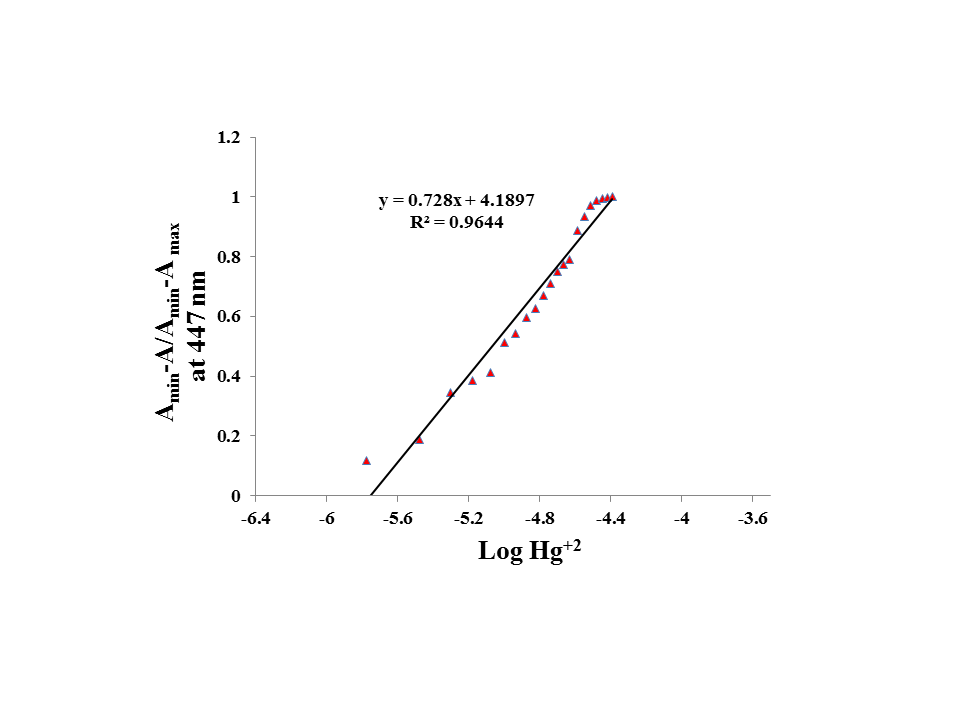


**Supplementary Figure S43:** Absorbance of **2** in CHCl_3_ normalized between maximum absorbance value at zero Hg^+2^ conc. and minimum absorbance value at 3.5 equiv. (4.55×10^-5^M) of Hg^+2^ ions (for calculating detection limit).


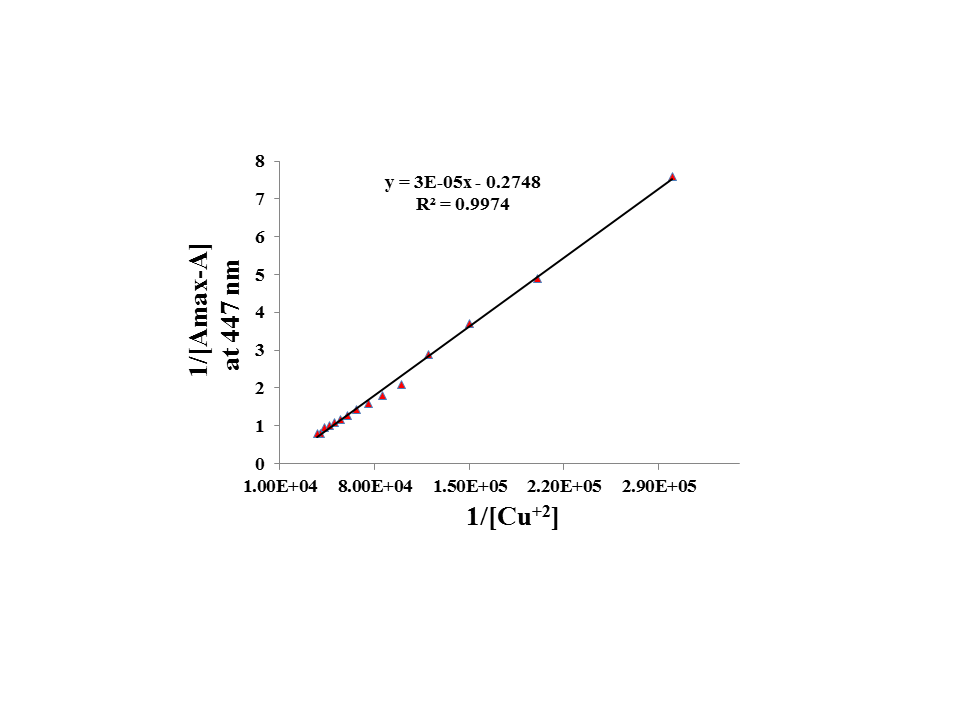


**Supplementary Figure S44:** BH Plot obtained from UV-Visible spectral titration of **2** with Cu^+2^ ions in distilled CHCl_3_ at 298 K indicating 1:1 stoichiometry.


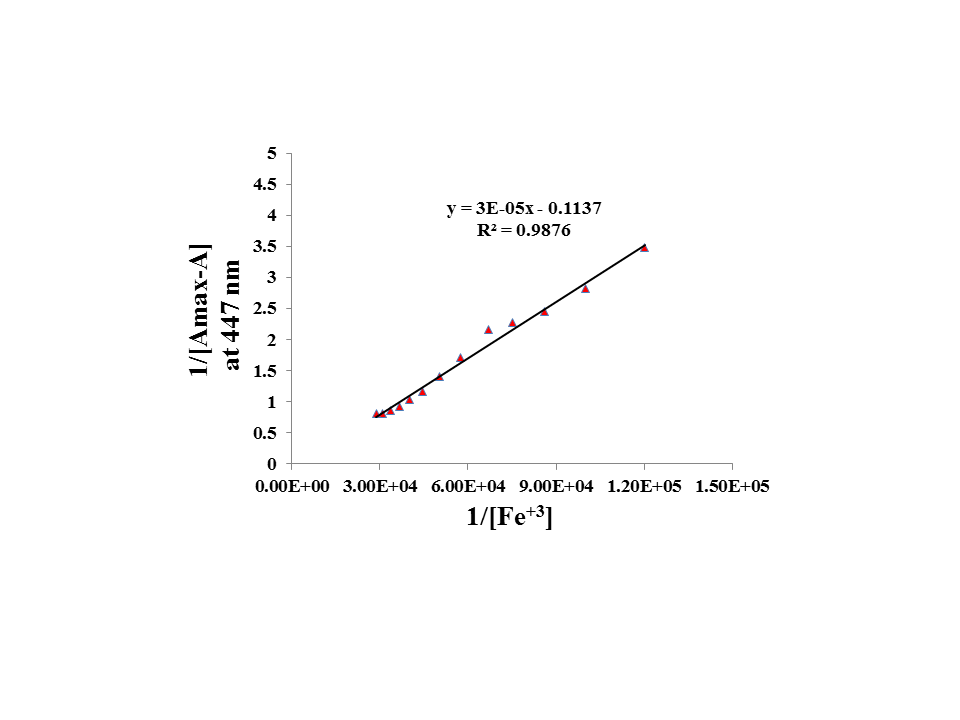


**Supplementary Figure S45:** BH Plot obtained from UV-Visible spectral titration of **2** with Fe^+3^ ions in distilled CHCl_3_ at 298 K indicating 1:1 stoichiometry.


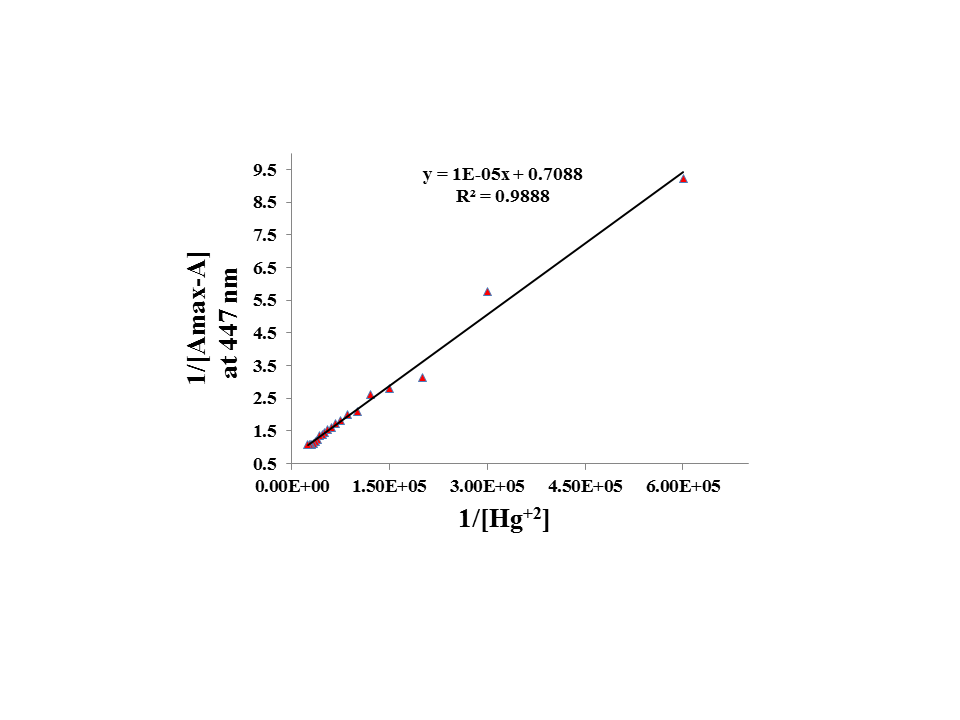


**Supplementary Figure S46:** BH Plot obtained from UV-Visible spectral titration of **2** with Hg^2+^ ions in distilled CHCl_3_ at 298 K indicating 1:1 stoichiometry.
